# Supplementary material for: Who pays and how much? A cross-sectional study of out-of-pocket payment for modern contraception in Kenya
Source: BMJ Open. 2019 Feb 20;9(2):e022414. doi: 10.1136/bmjopen-2018-022414 (PMC6398787; doi:10.1136/bmjopen-2018-022414)
Supplement: Supplementary data [file bmjopen-2018-022414supp001.pdf]

Supplementary Table 1

Summary of out-of-pocket payment (in USD) for injectable and implant users among users with non-zero expenditure, by most recent provider of the contraceptive method, with different methods for dealing with outliers.

a. Observations greater than two standard deviations from the mean recoded as equal to the mean.

	Govt hospital	Govt health centre	Govt dispensary	TOTAL PUBLIC	Private facility	NGO/faith-based facility	Pharmacy/ chemist	Other[1]	TOTAL
Injectable									
n	247	225	490	962	821	28	148	17	1,976
Mean cost in USD	$0.83	$0.78	$0.74	$0.75	$1.07	$1.01	$1.08	$1.06	$0.91
SD	0.412	0.399	0.385	0.343	0.277	0.560	0.278	0.280	0.349
25th percentile	$0.57	$0.57	$0.57	$0.57	$0.91	$0.57	$0.91	$1.14	$0.57
50th percentile (median)	$0.57	$0.57	$0.57	$0.57	$1.14	$0.80	$1.14	$1.14	$1.14
75th percentile	$1.14	$1.14	$1.14	$1.14	$1.14	$1.14	$1.14	$1.14	$1.14
Implant									
n	136	94	102	332	130	11	-	3	477
Mean cost in USD	$3.48	$2.90	$2.37	$2.97	$7.47	$6.43		$6.20	$4.31
SD	3.363	2.525	1.629	2.724	5.034	4.430		6.090	4.096
25th percentile	$2.28	$1.14	$1.14	$1.14	$3.42	$2.28		$1.14	$2.28
50th percentile (median)	$2.28	$2.28	$2.28	$2.28	$5.74	$9.12		$1.14	$2.28
75th percentile	$3.42	$3.42	$2.28	$3.42	$11.40	$9.12		$1.14	$5.70

b. Observations greater than two standard deviations from the mean recoded to missing.

	Govt hospital	Govt health centre	Govt dispensary	TOTAL PUBLIC	Private facility	NGO/faith-based facility	Pharmacy/ chemist	Other[1]	TOTAL
Injectable									
n	237	216	476	929	794	21	144	17	1,905
Mean cost in USD	$0.81	$0.74	$0.71	$0.74	$1.07	$0.82	$1.09	$1.06	$0.91
SD	0.387	0.327	0.330	0.346	0.282	0.338	0.282	0.280	0.355
25th percentile	$0.57	$0.57	$0.57	$0.57	$0.91	$0.57	$0.91	$1.14	$0.57
50th percentile (median)	$0.57	$0.57	$0.57	$0.57	$1.14	$0.80	$1.14	$1.14	$1.14
75th percentile	$1.14	$1.14	$1.14	$1.14	$1.14	$1.14	$1.14	$1.14	$1.14
Implant									
n	135	94	102	331	109	11	-	3	454
Mean cost in USD	$3.47	$2.90	$2.37	$2.97	$7.81	$6.43	-	$6.20	$4.24
SD	3.367	2.525	1.629	2.724	5.454	4.430	-	6.090	4.183
25th percentile	$2.28	$1.14	$1.14	$1.14	$2.85	$2.28	-	$1.14	$2.28
50th percentile (median)	$2.28	$2.28	$2.28	$2.28	$5.70	$9.12	-	$1.14	$2.28
75th percentile	$3.42	$3.42	$2.28	$3.42	$11.40	$9.12	-	$11.40	$5.70

c. Observations greater than two standard deviations from the mean recoded as equal to the value two standard deviations from the me

	Govt hospital	Govt health centre	Govt dispensary	TOTAL PUBLIC	Private facility	NGO/faith-based facility	Pharmacy/ chemist	Other[1]	TOTAL
Injectable									
n	247	225	490	962	821	28	148	17	1,976
Mean cost in USD	$0.86	$0.80	$0.75	$0.79	$1.11	$1.13	$1.11	$1.06	$0.95
SD	0.454	0.419	0.396	0.419	0.331	0.628	0.319	0.280	0.412
25th percentile	$0.57	$0.57	$0.57	$0.57	$0.91	$0.57	$0.91	$1.14	$0.57
50th percentile (median)	$0.57	$0.57	$0.57	$0.57	$1.14	$0.80	$1.14	$1.14	$1.14
75th percentile	$1.14	$1.14	$1.14	$1.14	$1.14	$1.71	$1.14	$1.14	$1.14
Implant									
n	136	94	102	332	130	11	-	3	477
Mean cost in USD	$3.54	$2.90	$2.37	$3.00	$10.16	$6.43	-	$6.20	$5.06
SD	3.559	2.525	1.629	2.829	7.236	4.430	-	6.090	5.528
25th percentile	$2.28	$1.14	$1.14	$1.14	$3.42	$2.28	-	$1.14	$2.28
50th percentile (median)	$2.28	$2.28	$2.28	$2.28	$6.84	$9.12	-	$1.14	$2.28
75th percentile	$3.42	$3.42	$2.28	$3.42	$17.10	$9.12	-	$11.40	$5.70

d. Observations greater than 1.5 times the interquartile range recoded to missing.

	Govt hospital	Govt health centre	Govt dispensary	TOTAL PUBLIC	Private facility	NGO/faith-based facility	Pharmacy/ chemist	Other[1]	TOTAL
Injectable									
n	237	216	476	929	794	21	144	17	1,905
Mean cost in USD	$0.81	$0.74	$0.71	$0.74	$1.07	$0.82	$1.09	$1.06	$0.91
SD	0.387	0.327	0.330	0.346	0.282	0.338	0.282	0.280	0.355
25th percentile	$0.57	0.57	0.57	$0.57	0.912	0.57	0.912	1.14	$0.57
50th percentile (median)	$0.57	0.57	0.57	$0.57	1.14	0.798	1.14	1.14	$1.14
75th percentile	$1.14	1.14	1.14	$1.14	1.14	1.14	1.14	1.14	$1.14
Implant									
n	128	89	102	319	72	9	-	2	401
Mean cost in USD	$2.80	$2.43	$2.37	$2.56	$4.33	$5.12	-	$1.14	$2.93
SD	1.738	1.565	1.629	1.663	2.060	3.647	-	0.000	1.948
25th percentile	$2.28	$1.14	$1.14	$1.14	$2.28	$2.28	-	$1.14	$1.71
50th percentile (median)	$2.28	$2.28	$2.28	$2.28	$3.42	$2.28	-	$1.14	$2.28
75th percentile	$3.42	$2.85	$2.28	$3.42	$5.70	$9.12	-	$1.14	$3.42

e. Observations greater than 1.5 times the interquartile range recoded as equal to the value 1.5 times the interquartile range.

	Govt hospital	Govt health centre	Govt dispensary	TOTAL PUBLIC	Private facility	NGO/faith-based facility	Pharmacy/ chemist	Other[1]	TOTAL
Injectable									
n	247	225	490	962	821	28	148	17	1,976
Mean cost in USD	$0.86	$0.80	$0.75	$0.79	$1.10	$1.11	$1.11	$1.06	$0.95
SD	0.445	0.408	0.388	0.410	0.323	0.595	0.313	0.280	0.403
25th percentile	$0.57	$0.57	$0.57	$0.57	$0.91	$0.57	$0.91	$1.14	$0.57
50th percentile (median)	$0.57	$0.57	$0.57	$0.57	$1.14	$0.80	$1.14	$1.14	$1.14
75th percentile	$1.14	$1.14	$1.14	$1.14	$1.14	$1.71	$1.14	$1.14	$1.14
Implant									
n	136	94	102	332	130	11	-	3	477
Mean cost in USD	$3.25	$2.87	$2.37	$2.87	$7.27	$6.14	-	$5.92	$4.18
SD	2.515	2.424	1.629	2.274	3.583	3.994	-	5.751	3.394
25th percentile	$2.28	$1.14	$1.14	$1.14	$3.42	$2.28	-	$1.14	$2.28
50th percentile (median)	$2.28	$2.28	$2.28	$2.28	$6.84	$9.12	-	$1.14	$2.28
75th percentile	$3.42	$3.42	$2.28	$3.42	$10.83	$9.12	-	$10.83	$5.70

[1] Includes DHS response options: mobile clinic and other private medical

SD: Standard deviation
