# Supplementary material for: Who pays and how much? A cross-sectional study of out-of-pocket payment for modern contraception in Kenya
Source: BMJ Open. 2019 Feb 20;9(2):e022414. doi: 10.1136/bmjopen-2018-022414 (PMC6398787; doi:10.1136/bmjopen-2018-022414)
Supplement: Supplementary data [file bmjopen-2018-022414supp003.pdf]

Supplementary Table 3

Among users with non-missing source of the method, percentage of modern contraceptive method by provider type.

	Govt hospital	Govt health center	Govt dispensary	Private facility	NGO/faith–based facility	Pharmacy/ chemist	Other[1]
**Method**							
Injectable	36.2%	49.4%	62.0%	65.6%	29.4%	24.6%	5.8%
Implant	27.4%	29.4%	20.9%	14.7%	17.8%	–†	10.7%
Condom	4.1%	3.1%	3.1%	1.6%	0.4%	13.2%	73.7%
Pill	7.3%	8.0%	9.2%	8.1%	4.6%	61.6%	7.8%
IUD	9.4%	5.6%	2.6%	7.3%	18.2%	–†	0.7%
Other modern methods[2]	15.7%	4.5%	2.2%	2.8%	29.7%	0.5%	1.3%
TOTAL	100%	100%	100%	100%	100%	100%	100%
Total number of users (weighted n)	*1,106*	*863*	*1,304*	*1,313*	*129*	*622*	*379*

[1] Includes DHS response options: mobile clinic and other private medical

[2] Includes other modern FP methods such as female and male sterilisation and female condoms.

† No respondents reported this provider and method.
