# Supplementary material for: Who pays and how much? A cross-sectional study of out-of-pocket payment for modern contraception in Kenya
Source: BMJ Open. 2019 Feb 20;9(2):e022414. doi: 10.1136/bmjopen-2018-022414 (PMC6398787; doi:10.1136/bmjopen-2018-022414)
Supplement: Supplementary data [file bmjopen-2018-022414supp004.pdf]

Supplementary Table 4

Proportion reporting free family planning by provider among users initiating their long–acting contraceptive method before or after June 2013 abolition of fees.

	Govt hospital % (95% CI)	Govt health centre % (95% CI)	Govt dispensary % (95% CI)	TOTAL PUBLIC % (95% CI)
Initiated before June 2013				
IUD (n=898)	49.9 (36.6–63.2)	78.8 (59.5–90.4)	76.9 (56.4–89.6)	61.2 (51.5–70.1)
Implant (n=469)	51.3 (40.6–61.9)	62.7 (51.5–72.7)	57.8 (48.3–66.7)	56.6 (50.4–62.7)
Initiated from June 2013 onward				
IUD (n=48)	48.5 (26.4–71.2)	69.2 (41.6–87.6)	64.6 (32.1–87.6)	59.2 (42.8–73.7)
Implant (n=357)	61.2 (50.5–70.9)	63.2 (50.0–74.7)	65.6 (56.0–74.0)	63.4 (56.9–69.5)
