# Supplementary material for: Who pays and how much? A cross-sectional study of out-of-pocket payment for modern contraception in Kenya
Source: BMJ Open. 2019 Feb 20;9(2):e022414. doi: 10.1136/bmjopen-2018-022414 (PMC6398787; doi:10.1136/bmjopen-2018-022414)
Supplement: Supplementary data [file bmjopen-2018-022414supp002.pdf]

Supplementary Table 2

Out-of-pocket payment (in USD) for injectable and implant across all provider types among users with non-zero expenditure by socio–demographic characteristics.

	Injectable				*Implant*			
	n	Median	Mean (95% CI)		n	Median	Mean (95% CI)	
Wealth quintile								
Poorest	209	$0.80	$0.84 (0.77-0.91)		29	$2.28	$3.35 (1.88-4.81)	
Poor	417	$0.80	$0.82 (0.78-0.86)		89	$2.28	$2.78 (2.43-3.13)	
Middle	459	$0.80	$0.89 (0.84-0.93)	p<0.001	81	$2.28	$3.03 (2.54-3.53)	p<0.001
Wealthy	519	$1.14	$0.97 (0.93-1.01)		101	$2.28	$4.06 (2.83-5.30)	
Wealthiest	379	$1.14	$1.14 (1.08-1.21)		177	$5.70	$5.95 (4.74-7.17)	
Residence								
Urban	792	$1.14	$1.07 (1.03-1.11)	p<0.001	230	$3.42	$5.18 (4.15-6.21)	p=0.005
Rural	1191	$0.80	$0.85 (0.82-0.88)		246	$2.28	$3.49 (2.94-4.04)	
Educational attainment								
Less than primary	615	$0.91	$0.87 (0.84-0.91)		115	$2.28	$3.88 (2.90-4.85)	
Less than secondary	915	$1.14	$0.94 (0.90-0.98)	p<0.001	202	$2.28	$3.36 (2.78-3.94)	p=0.004
Secondary+	453	$1.14	$1.03 (0.98-1.08)		160	$3.42	$5.81 (4.49-7.14)	
Age group								
<20 years	77	$1.14	$0.92 (0.85-1.00)		9	$4.56	$3.50 (1.95-5.04)	
20–29 years	1032	$1.14	$0.94 (0.90-0.98)	p=0.897	226	$2.28	$4.20 (3.47-4.93)	p=0.594
30+ years	874	$1.14	$0.94 (0.90-0.97)		242	$2.28	$4.44 (3.50-5.38)	
Region†								
Central	207	$1.14	$1.07 (1.01-1.14)		87	$3.42	$4.51 (3.47-5.56)	
Coast	125	$1.14	$0.99 (0.87-1.11)		11	$2.28	$4.32 (1.35-7.29)	
Eastern	425	$0.91	$0.91 (0.85-0.96)		67	$3.42	$4.72 (3.79-5.64)	
Nairobi	183	$1.14	$1.18 (1.05-1.30)	p<0.001	51	$5.74	$8.03 (4.27-11.78)	p<0.001
Nyanza	315	$0.57	$0.84 (0.79-0.90)		66	$2.28	$2.90 (2.11-3.70)	
Rift Valley	495	$1.14	$0.93 (0.89-0.98)		129	$2.28	$4.08 (3.13-5.02)	
Western	232	$0.80	$0.81 (0.75-0.87)		64	$2.28	$2.58 (2.08-3.07)	

†Due to the very low modern contraceptive prevalence (<5%), results for the North Eastern region are not presented.
