# Supplementary material for: Who pays and how much? A cross-sectional study of out-of-pocket payment for modern contraception in Kenya
Source: BMJ Open. 2019 Feb 20;9(2):e022414. doi: 10.1136/bmjopen-2018-022414 (PMC6398787; doi:10.1136/bmjopen-2018-022414)
Supplement: Author's manuscript [file bmjopen-2018-022414.draft_revisions.pdf]

BMJ Open

BMJ Open is committed to open peer review. As part of this commitment we make the peer review history of every article we publish publicly available.

When an article is published we post the peer reviewers' comments and the authors' responses online. We also post the versions of the paper that were used during peer review. These are the versions that the peer review comments apply to.

The versions of the paper that follow are the versions that were submitted during the peer review process. They are not the versions of record or the final published versions. They should not be cited or distributed as the published version of this manuscript.

BMJ Open is an open access journal and the full, final, typeset and author-corrected version of record of the manuscript is available on our site with no access controls, subscription charges or pay-per-view fees (http://bmjopen.bmj.com).

If you have any questions on BMJ Open's open peer review process please email info.bmjopen@bmj.com

BMJ Open

Who pays and how much? A cross-sectional study of out-of-pocket payment for modern contraception in Kenya

Journal:	*BMJ Open*
Manuscript ID	bmjopen-2018-022414
Article Type:	Research
Date Submitted by the Author:	16-Feb-2018
Complete List of Authors:	Radovich, Emma; London School of Hygiene and Tropical Medicine, Faculty of Epidemiology & Population Health<br>Dennis, Mardieh; London School of Hygiene and Tropical Medicine, Faculty of Epidemiology & Population Health<br>Barasa, Edwine; KEMRI-Wellcome Trust Research Programme, Health Economics Research Unit<br>Cavallaro, Francesca; London School of Hygiene and Tropical Medicine, Faculty of Epidemiology & Population Health<br>Wong, Kerry; London School of Hygiene and Tropical Medicine, Faculty of Epidemiology & Population Health<br>Borghi, Josephine; London School of Hygiene and Tropical Medicine, Faculty of Public Health & Policy<br>Lynch, Caroline; London School of Hygiene and Tropical Medicine, Faculty of Epidemiology & Population Health<br>Lyons-Amos, Mark; London School of Hygiene and Tropical Medicine, Faculty of Epidemiology & Population Health<br>Abuya, Timothy; Population Council Kenya<br>Benova, Lenka; London School of Hygiene and Tropical Medicine, Faculty of Epidemiology & Population Health
Keywords:	Kenya, family planning, user fees, Demographic & Health Survey, private sector, government provision

SCHOLARONE™
Manuscripts

Who pays and how much? A cross-sectional study of out-of-pocket payment for modern contraception in Kenya

Authors: Emma Radovich[1], Mardieh L. Dennis[1], Edwine Barasa[3], Francesca Cavallaro[1], Kerry L.M. Wong[1], Josephine Borghi[2], Caroline A. Lynch[1], Mark Lyons-Amos[1], Timothy Abuya[4], and Lenka Benova[1]

[1] Faculty of Epidemiology and Population Health, London School of Hygiene & Tropical Medicine, London, UK
[2] Faculty of Public Health and Policy, London School of Hygiene & Tropical Medicine, London, UK
[3] Health Economics Research Unit, KEMRI-Wellcome Trust Research Programme, Nairobi, Kenya
[4] Population Council, Nairobi, Kenya

Corresponding author:
Emma Radovich, London School of Hygiene & Tropical Medicine
Keppel Street
London WC1E 7HT
United Kingdom
+44(0)20 7612 7922
emma.radovich@lshtm.ac.uk

Word count: 4,123

Keywords: Kenya; family planning; user fees; Demographic & Health Survey; private sector; government provision

For peer review only

ABSTRACT (300 words)

Objectives: Out-of-pocket (OOP) payment for modern contraception is an under-studied component of healthcare financing in countries like Kenya, where wealth gradients in met need have prompted efforts to expand access to free contraception. This study aims to examine whether, among public sector providers, the poor are more likely to receive free contraception and to compare how OOP payment for injectables and implants—two popular methods—differs by public/private provider type and user's socio-demographic characteristics.

Design, setting and participants: Secondary analyses of nationally representative, cross-sectional household data from the 2014 Kenya Demographic and Health Survey. Respondents were women of reproductive age (15-49 years). The sample comprised 5,717 current modern contraception users, including 2,691 injectable and 1,073 implant users with non-missing expenditure values.

Main outcome: Respondent's self-reported source and payment to obtain their current modern contraceptive method.

Methods: We used multivariable logistic regression to examine predictors of free public sector contraception and compared average expenditure for injectable and implant. Quintile ratios examined progressivity of non-zero expenditure by wealth.

Results: Half of public sector users reported free contraception; this varied considerably by method and region. Users of implants, condoms, pills and IUDs were all more likely to report receiving their method for free ($p<0.001$) compared to injectable users. The poorest were as likely to pay for contraception as the wealthiest users at public providers (OR: 1.10, 95%CI: 0.64-1.91). Across all providers, among users with non-zero expenditure, injectable and implant users reported a mean OOP payment of KES 80 (US $0.91), 95%CI: KES 78-82 and KES 378 (US $4.31), 95%CI: KES 327-429, respectively. In the public sector, expenditure was pro-poor for injectable users yet weakly pro-rich for implant users.

Conclusions: More attention is needed to targeting subsidies to the poorest and ensuring government facilities are equipped to cope with lost user fee revenue.

ARTICLE SUMMARY

Strengths and limitations of this study

- A major strength of the study is that it is the first to our knowledge to use nationally representative data from a low-income country to examine out-of-pocket payment for modern contraception.
- Another strength is the transparency in the classification of family planning providers, handling of outliers and appropriate adjustments for complex survey design.
- One limitation of the study is the reliance on self-reported cost data from current users of modern contraception and the inability to compare this with costs to women who discontinued or eschewed use of modern contraception.

For peer review only - http://bmjopen.bmj.com/site/about/guidelines.xhtml

BACKGROUND

Sub-Saharan Africa has some of the highest rates of unmet need for family planning, and there are steep gradients favoring the rich in both met need for modern contraception and use of the private sector[1], where patients often pay out-of-pocket for services. This is mirrored in Kenya where unmet need is highest among the poor, and there is an 8-14 percentage point increase in modern contraception use with each increase in household wealth quintile[2]. Achieving Universal Health Coverage—including for family planning (FP) services—demands attention to financial protection and whether the ability to pay restricts individuals from accessing needed healthcare. Consideration of user fees is particularly important in countries like Kenya, where out-of-pocket (OOP) payments form a substantial proportion of healthcare financing[3,4]. Kenya has experimented with introducing and then removing or reducing user fees for various health services, often to account for shortages or changed priorities in donor funding[3,5], and this has important implications for equity in service access[2,6]. A study in Kenya and India found that poor households spend a significantly higher proportion of their income on reproductive health care (including FP), with the poorest households in Kenya spending 10 times the proportion spent by the least poor[7]. Many government financial protection policies focus on inpatient events where healthcare expenditure is likely to be catastrophic, yet the greater frequency of outpatient expenses—including for contraceptive services, which affect women in particular—can also push households into poverty[8] or reduce care-seeking among the poor[2,9].

A recent systematic review on the relationship between user fees and FP use in low- and middle-income countries (LMICs) was inconclusive, though some included studies suggested that young people and the poor were more sensitive to price increases than wealthier or less marginalised groups[10]. Cost is rarely cited as the reason for non-use of modern contraception among women in need (those wishing to delay or avoid pregnancy) in Demographic and Health Surveys (DHS) [11,12]. Yet, focus groups in Nyanza Province, Kenya found that the poor identify high cost of services as a barrier to FP care, both in opportunity costs associated with seeking care and direct fees for services[2], suggesting that for some individuals, cost can impact FP access.

Kenya has used various financing mechanisms to try and increase access to FP and reproductive health services, including cost-sharing, fee waivers at primary care facilities for low-income patients, social insurance (not focused on FP) and a pilot voucher program for long-acting contraception in the private sector and government facilities[2,13]. In the 1970s and 1980s, many LMICs instituted free or highly subsidised FP care in the public sector, regardless of users' ability to pay[14]. In 1965, Kenya removed user fees at public facilities. Fees were reintroduced in 1989 at all levels of public care—a decision reversed in 1990. However, due to budgetary constraints, user fees were subsequently reintroduced in 1992[15]. In response to declines in health service utilisation, a 2004 policy, commonly known as the "10/20 policy", abolished user fees in primary care facilities; instead government dispensaries and health centres were allowed to charge a registration fee of 10 or 20 Kenyan shillings (KES) (approximately US $0.11 and $0.23), with the poor exempted from payment[16,17]. Public hospitals were allowed to continue charging fees to users. Yet fee waiver implementation and identification of eligible individuals was left to the discretion of actors at the community and facility-level. Despite the 10/20 policy, many FP clients in government facilities reported paying additional 'hidden fees' for the consultation, medical tests or equipment, and the contraceptive commodity[2]. A 2010 health facility survey found that approximately 70% of government facilities providing FP charged user fees for services and nearly 60% did not post the fee schedule for clients to see[18]. A 2009 study found low community knowledge of the health fee policy and qualifying exemptions[16]. However, as of June 2013, all fees at government dispensaries and health centres, including those under the 10/20 policy, were eliminated[17]. Kenya's national reproductive health strategy enshrines access to modern contraception as a right, and FP services are intended to be provided for free at all public facilities[19,20].

For peer review only - http://bmjopen.bmj.com/site/about/guidelines.xhtml

Efforts to achieve universal coverage for reproductive health have led to increasing calls by donors and others for a "total market approach" in considering the different contributions of public and private providers. In this approach, government- or otherwise-subsidised services are targeted to meet the needs of the poor while individuals with the ability to pay are indirectly encouraged to seek FP services from commercial or unsubsidised private providers[2,8,14,21]. Kenya's changing fee policies within the public sector and the country's growing private sector, which now owns half of all health facilities[17], raise questions about how differing financing mechanisms may influence where individuals, especially the poor, seek FP and what this means for their OOP payment for modern contraception. Little is known about OOP payment to obtain modern contraception in sub-Saharan Africa, and in Kenya in particular, and how this varies by provider type. In the context of limited resources to expand FP access[22], it is important to understand the burden of user fees—who pays and how much—and the degree to which vulnerable groups are served by current efforts to provide affordable care in order to identify areas and groups in need of greater intervention.

This paper aims to address these knowledge gaps by describing FP sources by user's wealth in Kenya, examining whether, among public sector providers, the poor are more likely to receive free FP services, and comparing how payment for injectables and implants—the two most commonly used methods—differs by FP provider type and the user's socio-demographic characteristics.

METHODS

Data source

We used data from the most recent Kenya DHS (2014), a nationally representative, cross-sectional household survey of women age 15-49 with a multi-level cluster sampling design. Women in half of the households in the Kenya 2014 DHS were administered a short Woman's Questionnaire[23], which did not ask respondents for the amount paid for their current contraceptive method, and were excluded from our analysis.

Study populations

We examined data from three populations of women: 1) current users of modern contraception[24]; 2) users of IUD, implant, injectable, pill and male condom as these users were asked to self-report the amount paid to obtain their method (including both cost of the commodity and any consultation fees) during their most recent (re-)supply visit; and 3) users of injectable and implant, where estimates of OOP payment refer to a single quantity of the contraceptive, as users can receive only one "dose" during insertion or re-supply. Respondents with missing or "don't know" expenditure values accounted for 4.4% of all users in group two, and less than 1% of injectable and implant users, and were excluded from analysis.

Definitions

We classified women's self-reported most recent source of modern FP into seven provider categories: 1) government hospital; 2) government health centre; 3) government dispensary; 4) private facility, a constructed category comprising DHS response options of private hospital/clinic and private nursing/maternity home; 5) NGO/faith-based facility; 6) pharmacy/chemist; and 7) other, a constructed category of the response options: shop, mobile clinic, friend/relative, other, community health worker, community-based distributor and other private medical. We defined the public sector to be government-provided services (categories 1-3) and non-public providers to be categories 4-7. Less than 1% of all current modern contraceptive users were missing the source of their method and were excluded from analysis.

We examined three measures of the respondents' socio-economic status: household wealth quintiles derived by the DHS from household assets[25], urban/rural residence, and three levels of educational attainment: less than primary school (respondents with no education and those who started but did not

For peer review only - http://bmjopen.bmj.com/site/about/guidelines.xhtml

complete primary school), less than secondary school (respondents with complete primary or incomplete secondary school) and secondary+ (respondents with complete secondary or some higher education). We used DHS categories for respondent's current marital status (never, currently or formerly in union) and grouped respondents by their current age: less than 20, 20-29 and 30+ years. Kenya is administratively divided into 47 counties; however the variable for OOP payment for contraception on the 2014 Kenya DHS was intended to provide representative estimates for the national level, for urban and rural areas, and for the eight regions (former provinces)[23].

Analysis of free or 'registration fee only' FP in the public sector

We limited analysis of free FP to users whose most recent source of the method was a public sector provider. Adjusted Wald tests were performed to compare proportions. Bivariable and multivariable logistic regression was used to examine predictors, such as wealth quintile, provider category and region, of receiving free FP from public primary care facilities under the 2013 policy.

Users of long-acting methods like IUD and implant could report OOP payment based on FP consultations before the June 2013 abolishment of fees. Thus, estimates of free FP for IUD and implant were further disaggregated by whether the method was obtained before or after June 2013 based on the respondent's self-reported month and year of initiating use of the method.

Facility-level implementation of the June 2013 policy abolishing all fees at public primary care facilities may not have been immediate. As such, we additionally examined the proportion of users who reported paying up to 10 KES or 20 KES at a government dispensary or health centre, respectively, referring to these users as paying 'registration fees only' consistent with the former 10/20 policy, though respondents did not indicate the reason for the charge.

Analysis of OOP payment for injectable and implant

Prior to analysis, we assessed the data for improbable values and recoded observations to missing if reported expenditure was greater than 10 times the 95th percentile (six observations). Among injectable and implant users reporting non-zero cost, we described the patterns of OOP expenditure, reporting mean and median values. We conducted sensitivity analyses to ensure the robustness of our results, comparing results from multiple methods for dealing with outliers[26]; results did not differ substantially (Supplementary Table 1). For this analysis, observations greater than two standard deviations (SD) from the mean (2.7% and 2.1% of injectable and implant users, respectively) were recoded to be equal to the mean. Simple linear regression and marginal effects were used to compare means between providers and user characteristics. We additionally present estimates of OOP payment converted from KES to US dollars (USD) based on 1 KES to 0.0114 USD conversion rate for the midpoint of fieldwork in July 2014[27] in Supplementary Tables 1-2.

Equity of OOP payment for injectable and implant

Quintile ratios were used to measure the progressiveness of OOP payments for injectables and implants overall and within the public sector. This measure of equity in expenditure assumes that individuals in the lowest wealth quintile have less capacity to pay and thus if they spend the same or more as those in the highest quintile, this represents a greater proportion of income and constitutes regressive spending[28]. Quintile ratios were calculated by comparing mean expenditure in the wealthiest and poorest wealth quintiles and testing for differences in using an adjusted Wald-type test of nonlinear hypotheses based on the delta method, attributing significance at a 95% confidence level[7,28,29]. We define expenditure as weakly pro-rich if there was no significant difference in mean payment between the poorest and wealthiest users and strongly pro-rich if the poorest users paid significantly more than the wealthiest users (quintile ratio <1)[7,28].

For peer review only - http://bmjopen.bmj.com/site/about/guidelines.xhtml

All analysis used women's individual sampling weights and standard error adjustment to account for complex survey design. Analyses were conducted in Stata/SE v14 (StataCorp, College Station, TX, USA).

RESULTS

A total of 5,717 modern contraceptive users with non-missing provider data were included in our analysis sample.

Methods and sources of family planning

Among all current FP users, the wealthiest quintile had the broadest mix of methods, with no single method accounting for more than a third of current modern FP users (Figure 1a). In contrast, method mix among users in the three poorer quintiles was dominated by injectables, which accounted for more than half of methods used. While injectables and implants were the two most popular methods for all users, this was particularly true for the poorest users, where these two methods accounted for nearly 80% of all modern methods used.

The wealthiest contraceptive users also reported a broader mix of providers (Figure 1b). Among the poorest users, 80.0% reported a public sector source. Public provider use decreased steadily and use of private facilities and pharmacies increased with increasing wealth quintile. The wealthiest users reported the largest use of private facilities (30.5%) and pharmacies (18.7%). Among injectable users, public sector providers were the most-used source for the three poorest quintiles, with a clear decline in government dispensary use with increasing wealth (Figure 1c). The vast majority of implants in the four poorer wealth quintiles were sourced from public providers, and there was a dramatic increase in use of private facilities for implants in the fifth, wealthiest quintile.

Free family planning

Overall, 51.1% of public sector users reported obtaining their modern FP method for free at their most recent visit (Table 1). This varied by method used: more than 90% of condom users compared to 40.7% of injectable users reported free FP. Across the three levels of facilities, 50.1% of government hospital, 56.2% of government health centre, and 48.5% of government dispensary users reported free FP, with weak evidence of a difference by facility level (p=0.048). The percentage of women obtaining free FP in public facilities differed only slightly by respondent's wealth quintile, urban/rural residence, education level, or age group. The proportion of users reporting free FP varied considerably by region, with 39.4% of Rift Valley residents compared to 76.6% of Nairobi residents reporting free contraception. Additionally, 1.3% (95% CI: 0.9-2.1%) of users of government health centres and dispensaries reported paying a 'registration fee only' amount under the former 10/20 policy (results not shown). There was no difference by user's wealth quintile.

For peer review only - http://bmjopen.bmj.com/site/about/guidelines.xhtml

Table 1: Among public sector providers, proportion reporting free family planning by modern method users' socio–demographic characteristics

	Govt hospital (n=929)	Govt health centre (n=815)	Govt dispensary (n=1,267)	TOTAL PUBLIC (n=3,011)
Overall (95% CI)	50.1 (45.9–54.3)	56.2 (50.9–61.4)	48.5 (45.0–52.1)	51.1 (48.5–53.7)
Method				
Injectable	38.4 (32.4–44.8)	46.0 (39.3–52.9)	39.2 (34.8–43.7)	40.7 (37.5–44.1)
Implant	55 (47.1–62.6)	63.0 (53.9–71.1)	61.5 (54.6–67.9)	59.6 (54.8–64.2)
Pill	68.7 (53.8–80.6)	66.0 (50.6–78.6)	61.1 (50.0–71.3)	64.7 (57.1–71.6)
Condom	90.8 (71.0–97.5)	92.8 (77.4–98.0)	97.4 (83.7–99.6)	93.6 (85.0–97.3)
IUD	49.6 (38.6–60.7)	75.4 (57.8–87.3)	73.4 (57.1–85.1)	60.7 (52.8–68.1)
Wealth quintile				
Poorest	61.7 (48.7–73.1)	54.1 (41.3–66.5)	46.1 (39.2–53.1)	50.2 (44.4–55.9)
Poor	51.2 (41.0–61.4)	51.7 (42.2–61.1)	44.3 (38.4–50.4)	47.6 (43.0–52.3)
Middle	43.2 (35.5–51.2)	52.4 (42.6–62.0)	48.9 (41.3–56.5)	48.6 (43.6–53.7)
Wealthy	51.8 (43.6–60.0)	57.1 (47.3–66.3)	54.4 (45.9–62.7)	54.2 (48.6–59.7)
Wealthiest	49.6 (42.0–57.2)	66.9 (55.0–76.9)	53.9 (39.8–67.4)	54.9 (48.7–61.0)
Residence				
Urban	49.1 (43.6–54.7)	66.0 (56.8–74.2)	56.1 (47.6–64.3)	55.2 (50.6–59.7)
Rural	51.6 (45.1–58.0)	50.9 (44.7–57.0)	46.8 (42.9–50.7)	48.8 (45.8–51.9)
Educational attainment				
Less than primary	56.7 (48.3–64.7)	55.1 (47.0–62.9)	47.6 (42.5–52.6)	51.7 (47.8–55.6)
Less than secondary	46.0 (40.0–52.0)	56.6 (49.3–63.6)	48.9 (43.8–53.9)	49.9 (46.4–53.5)
Secondary+	51.3 (43.0–59.4)	57.1 (46.9–66.6)	49.8 (40.9–58.7)	52.5 (47.0–58.0)
Age group				
<20 years	41.3 (19.4–67.4)	61.3 (40.2–78.9)	60.4 (42.8–75.7)	55.4 (43.4–66.9)
20–29 years	45.8 (40.0–51.7)	55.3 (48.1–62.4)	42.9 (37.9–48.1)	47.0 (43.5–50.6)
30+ years	54.2 (48.0–60.3)	56.5 (49.8–63.0)	52.7 (47.8–57.5)	54.2 (50.7–57.7)
*Region**				
Central	53.1 (43.5–62.4)	64.0 (50.7–75.4)	60.2 (47.8–71.4)	58.6 (51.3–65.5)
Coast	70.3 (58.9–79.7)	81.2 (69.6–89.0)	62.4 (52.4–71.4)	69.1 (62.1–75.2)
Eastern	35.2 (26.3–45.2)	40.4 (29.5–52.3)	44.5 (36.9–52.4)	41.5 (36.0–47.2)
Nairobi	70.4 (54.1–82.7)	76.0 (55.3–89.0)	–†	76.6 (63.4–86.0)
Nyanza	59.4 (49.8–68.3)	55.6 (44.2–66.4)	37.0 (30.0–44.7)	49.0 (43.1–55.0)
Rift Valley	30.9 (24.2–38.5)	48 (37.3–58.9)	42.8 (36.7–49.1)	39.4 (35.1–43.8)
Western	60.1 (43.6–74.5)	46.6 (34.2–59.4)	50 (39.8–60.3)	50.6 (43.5–57.7)

*Due to the very low modern contraceptive prevalence (<5%), results for the North Eastern region are not presented.

†No respondents reported this provider.

Among non-public sector providers (results not shown), 10.9% of private facility users and less than 1% of pharmacy users reported free family planning. Of the limited number of users of NGO/faith-based facilities (n=91), 30.9% reported obtaining their contraceptive method for free.

Supplementary Table 3 shows the proportion of IUD and implant users receiving free FP from public sector providers among users initiating the method before and after the June 2013 fee abolishment. Among implant users, the proportion receiving free FP from government health centres was similar between the two initiation periods and increased in the later period among users of government hospitals and dispensaries, though confidence intervals overlap. Among IUD users, the proportion receiving free care was slightly lower across all three public provider categories in the later initiation period, but differences were not statistically significant.

For peer review only - http://bmjopen.bmj.com/site/about/guidelines.xhtml

For peer review only

Table 2 shows the results of bivariable and multivariable analysis of receiving free modern FP among public primary care facility users. There were no differences by wealth quintile in the odds of obtaining free contraception after adjusting for method, provider type and user characteristics. Users of implants, condoms, pills and IUDs were all more likely to report receiving their method for free (p<0.001) compared to injectable users, and this relationship remained after adjusting for provider and user characteristics. Users in all regions had lower odds of free contraception compared to Nairobi, except Coast region (where it was not significantly different).

Table 2: Unadjusted and adjusted odds ratios from logistic regression analysis of reporting free family planning services from government primary care providers among modern method users

Variables	*Modern method users (n=2,079)*			
	Unadjusted		Adjusted	
	OR	(95% CI)	OR	(95% CI)
Wealth quintile				
Poorest	0.58*	(0.37-0.91)	1.10	(0.64-1.91)
Poor	0.55**	(0.36-0.85)	1.20	(0.71-2.03)
Middle	0.64*	(0.41-0.99)	1.25	(0.74-2.11)
Wealthy	0.79	(0.51-1.24)	1.16	(0.67-2.01)
Wealthiest	Ref		Ref	
Provider				
Govt. health centre	Ref		Ref	
Govt. dispensary	0.74*	(0.57-0.95)	0.95	(0.74-1.22)
Method				
injectable	Ref		Ref	
implant	2.32***	(1.78-3.02)	2.15***	(1.62-2.86)
condom	29.87***	(9.84-90.66)	35.29***	(11.42-109.05)
pill	2.39***	(1.63-3.52)	2.27***	1.56-3.28)
IUD	4.14***	(2.26-7.56)	3.90***	(2.06-7.36)
Residence				
Urban	Ref		Ref	
Rural	0.58***	(0.43-0.79)	0.83	(0.60-1.14)
Region				
Central	0.36*	(0.14-0.93)	0.31*	(0.10-0.93)
Coast	0.48	(0.18-1.24)	0.53	(0.19-1.53)
Eastern	0.17***	(0.07-0.42)	0.18**	(0.06-0.52)
Nairobi	Ref		Ref	
Nyanza	0.18***	(0.07-0.44)	0.17**	(0.06-0.50)
Rift Valley	0.17***	(0.07-0.43)	0.19**	(0.07-0.53)
Western	0.21**	(0.08-0.53)	0.21**	(0.07-0.62)
Age group				
<20 years	1.31	(0.75-2.28)	1.58	(0.85-2.92)
20–29 years	0.76*	(0.61-0.95)	0.85	(0.67-1.08)
30+ years	Ref		Ref	
Marital status				
Never in union	0.93	(0.62-1.40)	0.80	(0.47-1.36)
Currently in union	Ref		Ref	
Formerly in union	1.38†	(0.96-1.98)	1.27	(0.88-1.83)

Note: Asterisks and daggers indicate that differences between the category and the reference category are significant at *p<0.05, **p<0.01, ***p<0.001, or marginally significant at †p<0.1.

For peer review only - http://bmjopen.bmj.com/site/about/guidelines.xhtml

Out-of-pocket payment for injectables and implants

Among injectable and implant users reporting greater than zero OOP payment to obtain the method from their most recent provider (both sectors combined), the mean cost was KES 80 (US $0.91) (95% CI: KES 78-82) for injectable and KES 378 (US $4.31) (95% CI: KES 327-429) for implant (Table 3). 1.7% of injectable and 1.5% of implant users reported paying amounts consistent with registration fees only (<KES 20). OOP payment varied, particularly for implant, by source of the method, with some private facility users reporting very high costs. Injectable users of public sector providers reported a median cost of KES 50, whereas the median cost was twice that (KES 100) for those accessing private facilities or pharmacies. Among implant users, those accessing public sector sources reported a median cost of KES 200, compared to a median cost of KES 503 among those utilising private facilities.

When assessed by user characteristics, mean OOP payment for both injectables and implants varied significantly by user's wealth, residence, education level and region, but not by user's age (Table 4). Urban and Nairobi residents paid more for both methods; this was particularly notable for implant users in Nairobi, where mean cost was more than twice that of implant users in Western or Nyanza regions. Mean and median cost did not increase linearly with increasing wealth quintile. For injectable users, median cost in the poorest three quintiles was KES 70 compared to KES 100 in the two wealthiest quintiles. For implant users, median cost of KES 500 in the wealthiest quintile was more than twice the median cost of KES 200 in the four poorer quintiles. The overall quintile ratio for all providers comparing mean cost in the wealthiest quintile to the poorest quintile was 1.3 ($p<0.001$) for injectable and 1.8 ($p=0.007$) for implant, indicating strong evidence of pro-poor OOP payment for both methods. Among public sector users, the quintile ratio was 1.2 ($p=0.033$) for injectable indicating pro-poor expenditure, and 0.90 ($p=0.660$) for implant (Table 5), indicating weakly pro-rich expenditure (no difference in mean cost between the quintiles) for public sector implant users.

DISCUSSION

This is the first study to our knowledge to use nationally representative household data from an LMIC to examine equity of OOP payment for FP, comparing differences in cost to users accessing public and non-public providers. The wealthiest FP users in Kenya utilised a greater mix of modern methods and providers compared to the poorest users, and use of non-public providers increased with increasing wealth. Despite Kenya's national policy to offer free FP services at public facilities, we found only half of modern method users reported obtaining their method at no cost from government providers. There were no differences by user's socio-economic position. Among injectable and implant users reporting OOP expenditure, there were considerable differences by source of the method. Consistent with a previous study of FP users in urban Kenya[30], we found private facility and pharmacy users, unsurprisingly, reported higher expenditures than users of public facilities. Unfortunately, due to very small sample sizes (<30 users), OOP payment by users of NGO/faith-based facilities remains unclear, though there is some indication that costs may be higher than among public sector providers. Greater use of higher cost, non-public providers by the wealthiest users contributed to overall pro-poor expenditure, with both injectable and implant users in the wealthiest quintile paying significantly more than their counterparts in the poorest quintile.

Public sector implant payments were weakly regressive (no difference in mean payment between the poorest and wealthiest quintiles), yet recent attempts to expand access to long-term methods, like implants, in Kenya have focused on expanding the range of providers available to the poor through vouchers. In 2005, Kenya launched a pilot system in five districts that enabled individuals below the poverty threshold to purchase vouchers for long-term or permanent contraceptive methods, which could be redeemed at a variety of public, private for-profit and private not-for-profit providers[31]. The voucher programme received criticism concerning the limited uptake of the scheme[4,32] and lack of demand generation activities, and some suggested that the FP voucher fee of KES 100 fee (approximately US $1.25) was still

For peer review only - http://bmjopen.bmj.com/site/about/guidelines.xhtml

Table 3: Summary of out-of-pocket payment (in KES) for injectable and implant users among users with non-zero expenditure, by most recent provider of the contraceptive method

	Govt hospital	Govt health center	Govt dispensary	TOTAL PUBLIC	Private facility	NGO/faith-based facility	Pharmacy/ chemist	Other[1]	TOTAL
Injectable									
n	247	225	490	962	821	28	148	17	1,976
Mean cost	KES 72	KES 66	KES 63	KES 66	KES 94	KES 75	KES 95	KES 93	KES 80
SD	33.38	28.37	28.78	30.10	24.33	26.37	24.42	24.56	30.63
25th percentile	KES 50	KES 50	KES 50	KES 50	KES 80	KES 50	KES 80	KES 100	KES 50
50th percentile (median)	KES 50	KES 50	KES 50	KES 50	KES 100	KES 70	KES 100	KES 100	KES 100
75th percentile	KES 100	KES 100	KES 100	KES 100	KES 100	KES 87	KES 100	KES 100	KES 100
Reporting registration fees only	7.5%	5.3%	0.8%	3.6%					1.7%
Implant									
n	136	94	102	332	130	11	-	3	477
Mean cost	KES 305	KES 255	KES 208	KES 261	KES 655	KES 564		KES 544	KES 378
SD	295.01	221.51	142.92	238.98	441.62	388.58		534.17	359.25
25th percentile	KES 200	KES 100	KES 100	KES 100	KES 300	KES 200		KES 100	KES 200
50th percentile (median)	KES 200	KES 200	KES 200	KES 200	KES 503	KES 800		KES 100	KES 200
75th percentile	KES 300	KES 300	KES 200	KES 300	KES 1,000	KES 800		KES 1,000	KES 500
Reporting registration fees only	1.7%	5.0%	0.0%	2.1%					1.5%

[1] Includes DHS response options: mobile clinic and other private medical

SD: Standard deviation 1 KES = 0.0114 USD

For peer review only

For peer review only - http://bmjopen.bmj.com/site/about/guidelines.xhtml

Table 4: Out-of-pocket payment (in KES) for injectable and implant across all provider types among users with non-zero expenditure by socio–demographic characteristics

	Injectable n	Median	Mean	(95% CI)			Implant n	Median	Mean	(95% CI)		
Wealth quintile						Quintile ratio†						Quintile ratio†
Poorest	209	KES 70	KES 71	(66-77)			29	KES 200	KES 294	(165-422)		
Poor	417	KES 70	KES 71	(67-74)			89	KES 200	KES 244	(212-274)		
Middle	459	KES 70	KES 76	(73-79)	p<0.001		81	KES 200	KES 266	(223-309)	p<0.001	
Wealthy	516	KES 100	KES 83	(80-87)			101	KES 200	KES 357	(248-465)		
Wealthiest	379	KES 100	KES 96	(91-101)		1.3 (p<0.001)	177	KES 500	KES 522	(415-629)		1.8 (p=0.007)
Residence												
Urban	790	KES 100	KES 91	(88-94)	p<0.001		230	KES 200	KES 455	(364-545)	p=0.005	
Rural	1191	KES 70	KES 73	(71-75)			246	KES 300	KES 306	(258-355)		
Educational attainment												
Less than primary	615	KES 80	KES 75	(72-78)			115	KES 200	KES 340	(255-425)		
Less than secondary	915	KES 100	KES 80	(77-84)	p<0.001		202	KES 200	KES 295	(244-346)	p=0.004	
Secondary+	451	KES 100	KES 87	(83-91)			160	KES 300	KES 510	(394-626)		
Age group												
<20 years	77	KES 100	KES 81	(74-87)			9	KES 400	KES 307	(171-442)		
20–29 years	1030	KES 87	KES 80	(77-83)	p=0.928		226	KES 200	KES 369	(304-433)	p=0.594	
30+ years	874	KES 100	KES 80	(77-82)			242	KES 200	KES 389	(307-472)		
*Region**												
Central	207	KES 100	KES 90	(86-95)			87	KES 300	KES 396	(304-488)		
Coast	125	KES 100	KES 82	(73-92)			11	KES 200	KES 379	(119-639)		
Eastern	425	KES 80	KES 77	(73-81)			67	KES 300	KES 414	(333-495)		
Nairobi	183	KES 100	KES 101	(91-111)	p<0.001		51	KES 503	KES 704	(374-1034)	p<0.001	
Nyanza	315	KES 50	KES 72	(68-77)			66	KES 200	KES 255	(185-324)		
Rift Valley	492	KES 87	KES 80	(76-84)			129	KES 200	KES 358	(275-440)		
Western	232	KES 70	KES 69	(64-74)			64	KES 200	KES 226	(183-270)		

*Due to the very low modern contraceptive prevalence (<5%), results for the North Eastern region are not presented.

†Ratio of mean OOP expenditure comparing the wealthiest users to the poorest users. Adjusted Wald-type test based on the delta method was used to test for significance.
1 KES = 0.0114 USD

For peer review only - http://bmjopen.bmj.com/site/about/guidelines.xhtml

Table 5: Out-of-pocket payment (in KES) for public sector injectable and implant among users with non-zero expenditure by wealth quintile

	Injectable				Implant			
	n	Mean	(95% CI)	Quintile ratio†	n	Mean	(95% CI)	Quintile ratio†
Wealth quintile								
Poorest	147	KES 65	(59-72)		27	KES 267	(146-389)	
Poor	247	KES 61	(57-65)		76	KES 231	(200-262)	
Middle	256	KES 66	(62-70)		68	KES 253	(205-301)	
Wealthy	197	KES 66	(61-72)		78	KES 317	(187-447)	
Wealthiest	116	KES 78	(69-87)	1.2 (p=0.033)	82	KES 240	(190-291)	0.90 (p=0.660)

†Ratio of mean OOP expenditure comparing the wealthiest users to the poorest users. Adjusted Wald-type test based on the delta method was used to test for significance.
1 KES = 0.0114 USD

For peer review only

relatively costly for the poorest users[2]. Our findings highlight that greater attention is needed for the one-third of implant users in the public sector reporting OOP expenditure and ensuring payment at government providers is not a burden on the poorest users.

Respondent's region was significantly associated with differences in reporting free FP and the amount paid for injectable and implant. In 2010, Kenya's Ministry of Health devolved decision-making power and budgets to the county level, though policy continued to be set at the national level[33]. Despite recent gains in national modern contraceptive prevalence and reduction of unmet need, large regional disparities in coverage remain[23]. Differences in regional levels of free FP suggest that counties may be operating different systems of payment for contraception or distribution channels for FP commodities. Additionally, facility administrative and staffing costs likely vary depending on facility size and county-level cost of living.

Public facilities cannot directly charge for FP under current policy, so when faced with declining revenue, they may introduce indirect charges, framed as registration fees or other costs, to recoup expenses[16]. For example, the considerable variation in free services by method in public facilities possibly reflects differing auxiliary costs associated with dispensing methods, with, for example, more staff time and medical equipment required to insert IUDs and implants compared to condoms, which are often available without a consultation. We found that injectable users were significantly less likely to report receiving this method for free compared to long-acting IUD and implants or even the pill, raising questions about the long-term cost burden to users, who require frequent re-supply, for this popular method. Increased understanding of sub-national implementation of national FP policy is needed to ensure fair access to free contraceptives and preserve fragile, uneven gains in meeting demand for modern contraception.

Limitations

This study was limited in relying on the accuracy of women's self-report of their method, source and cost of FP. While current injectable users needed to recall how much was paid up to three months earlier, some current implant users were asked to report the amount paid up to three years prior to interview, though median length of implant use was less than 17 months. Additionally, we were only able to consider cost and source among women who were current users of FP. Findings are likely not generalisable to former implant or injectable users, particularly if they discontinued due to costs associated with obtaining their method of choice, or to prospective users who were discouraged from initiating FP due to costs associated.

We acknowledge that the first consultation visit to initiate the contraceptive method may be longer, involving counselling and taking of medical history, than a re-supply visit and could result in

For peer review only - http://bmjopen.bmj.com/site/about/guidelines.xhtml

increased cost. However, we compared results for initiators, users starting injectable and implant less than three months and three years, respectively, before the survey where the cost paid likely refers to the initiating consultation, against re-supply users, those starting the method more than three months or years prior to interview. Yet we found initiating users reported slightly lower mean costs than re-suppliers, though differences were not significant (results not shown).

The DHS question regarding contraceptive cost asked for the total paid for commodity and consultation, and it did not capture costs associated with time and travel to obtain the method. These may be significant, particularly for rural users. We were unable to estimate the share of OOP payment for FP from total income because the DHS does not collect information on household/individual income or expenditures. Additionally, DHS household wealth quintiles may not align with the poverty definition used to determine FP fee waivers or offer sufficient nuance to distinguish very disadvantaged households[34].

Finally, FP budget implementation is done at the county-level in Kenya, yet the DHS FP cost question was intended to be representative at national, urban/rural and regional levels only[23] and thus county-level results could not be examined.

Conclusions

A way of exempting the poor from payment is a core component of an equitable system of user fees for healthcare[3,35], yet our findings highlight that the poorest contraceptive users in the public sector were as likely to pay for FP services as wealthier users. Kenya's National Reproductive Health Strategy (2009-2015), for the first time, outlined pro-poor strategies and objectives to increase equity of FP access[2,19], including the abolishment of user fees for reproductive health services in public facilities, except for a registration fee of up to 20 KES. Yet a substantial proportion of injectable and implant users paid much more than this to obtain their chosen method. The government's commitment to expand FP access is admirable, but more attention is needed to implementation, particularly to account for geographic variation, and ensuring recent efforts to reimburse facilities for lost user fee revenue are done at appropriate levels.

Public sector resources alone are unlikely to meet Kenya's growing demand for modern contraception. Policymakers should consider how government resources could be targeted at those least able to tap the private sector for FP care. While individual price discrimination offers one route to targeting public services to the poor, efforts could also focus resources—including outreach campaigns about patients' rights and correct fees—toward facilities closest to where the poor live. Fulfilling the promise of equity in FP access in Kenya demands turning policy intention into sustainable action from the national to facility-level.

List of abbreviations

DHS: Demographic & Health Surveys **FP**: Family planning **IUD:** Intrauterine device
KES: Kenyan Shillings **LMICs**: Low- and middle-income countries
NGO: Non-governmental organisation **OOP:** Out-of-pocket **USD**: United States Dollar

DECLARATIONS

Ethics approval

The DHS receive government permission and follow ethical practices including informed consent and assurance of confidentiality. The Research Ethics Committee of the London School of Hygiene and Tropical Medicine approved our secondary-data analysis.

For peer review only - http://bmjopen.bmj.com/site/about/guidelines.xhtml

Data sharing

The data that support the findings of this study are owned by the Demographic and Health Surveys (DHS) Program, operated by ICF International. Restrictions apply to the availability of these data, which were used under license for the current study, and so are not publicly available. Data are available for free from the DHS Program website and available for researchers who apply for and meet the criteria for access. Legal access agreements do not allow the sharing of datasets to unregistered researchers.

Competing interests

The authors declare that they have no competing interests.

Funding statement

The research in this publication was supported by funding from MSD, through its MSD for Mothers program. MSD had no role in the design, collection, analysis and interpretation of data, in writing of the manuscript, or in the decision to submit the manuscript for publication. The content of this publication is solely the responsibility of the authors and does not represent the official views of MSD. MSD for Mothers is an initiative of Merck & Co., Inc., Kenilworth, N.J., U.S.A. Edwine Barasa is supported by a Wellcome Trust research training grant (#107527).

Authors' contributions

ER designed the research question, analysed data, and prepared the manuscript. LB, MD, CL and JB contributed to the design of the study. KW contributed to analysis of the data. LB, MD, FC and EB assessed interpretation of findings, and contributed to manuscript revisions. JB, TA, KW and MLA reviewed and edited the manuscript. All authors read and approved the final manuscript.

REFERENCES

1 Campbell OMR, Benova L, Macleod D, *et al.* Who, What, Where: An analysis of private sector family planning provision in 57 low- and middle-income countries. *Trop Med Int Heal* 2015;**20**:1639–56. doi:10.1111/tmi.12597

2 Health Policy Initiative. The EQUITY Framework: Influencing Policy and Financing Reforms to Increase Family Planning Access for the Poor in Kenya. 2010.

3 Munge K, Briggs AH. The progressivity of health-care financing in Kenya. *Health Policy Plan* 2014;**29**:912–20. doi:10.1093/heapol/czt073

4 Obare F, Warren C, Kanya L, *et al.* Community-level effect of the reproductive health vouchers program on out-of-pocket spending on family planning and safe motherhood services in Kenya. *BMC Health Serv Res* 2015;**15**:343. doi:10.1186/s12913-015-1000-3

5 Agha S, Do M. Does an expansion in private sector contraceptive supply increase inequality in modern contraceptive use? *Health Policy Plan* 2008;**23**:465–75. doi:10.1093/heapol/czn035

6 Chuma J, Maina T. Catastrophic health care spending and impoverishment in Kenya. *BMC Health Serv Res* 2012;**12**:413. doi:10.1186/1472-6963-12-413

7 Haghparast-Bidgoli H, Pulkki-Brännström A-M, Lafort Y, *et al.* Inequity in costs of seeking sexual and reproductive health services in India and Kenya. *Int J Equity Health* 2015;**14**:84. doi:10.1186/s12939-015-0216-5

8 Holtz J. Achieving Universal Access to Family Planning Services. 2016.

9 Chuma J, Gilson L, Molyneux C. Treatment-seeking behaviour, cost burdens and coping strategies among rural and urban households in Coastal Kenya: an equity analysis. *Trop Med Int Heal* 2007;**12**:673–86. doi:10.1111/j.1365-3156.2007.01825.x

For peer review only - http://bmjopen.bmj.com/site/about/guidelines.xhtml

10 Korachais C, Macouillard E, Meessen B. How User Fees Influence Contraception in Low and Middle Income Countries: A Systematic Review. *Stud Fam Plann* 2016;**47**:341–56. doi:10.1111/sifp.12005

11 Sedgh G, Ashford LS, Hussain R. Unmet Need for Contraception in Developing Countries: Examining Women’s Reasons for Not Using a Method. 2016.

12 Sedgh G, Hussain R. Reasons for Contraceptive Nonuse among Women Having Unmet Need for Contraception in Developing Countries. *Stud Fam Plann* 2014;**45**:151–69. doi:10.1111/j.1728-4465.2014.00382.x

13 Janisch CP, Albrecht M, Wolfschuetz A, *et al.* Vouchers for health: A demand side output-based aid approach to reproductive health services in Kenya. *Glob Public Health* 2010;**5**:578–94. doi:10.1080/17441690903436573

14 POLICY Project. Targeting: A Key Element of National Contraceptive Security Planning. 2003.

15 Barasa EW, Maina T, Ravishankar N. Assessing the impoverishing effects, and factors associated with the incidence of catastrophic health care payments in Kenya. *Int J Equity Health* 2017;**16**:31. doi:10.1186/s12939-017-0526-x

16 Chuma J, Musimbi J, Okungu V, *et al.* Reducing user fees for primary health care in Kenya: Policy on paper or policy in practice? *Int J Equity Health* 2009;**8**:15. doi:10.1186/1475-9276-8-15

17 Chuma J, Maina T. Free Maternal Care and Removal of User Fees at Primary-Level Facilities in Kenya: Monitoring the Implementation and Impact—Baseline Report. 2013.

18 National Coordinating Agency for Population and Development - NCAPD/Kenya, Ministry of Medical Services - MOMS/Kenya, Ministry of Public Health and Sanitation - MOPHS/Kenya, *et al.* Kenya Service Provision Assessment Survey 2010. Nairobi, Kenya: 2011.

19 Ministry of Public Health and Sanitation & Ministry of Medical Services. National Reproductive Health Strategy, 2009-2015.

20 Kenya Reproductive, Maternal, Newborn, Child and Adolescent Health (RMNCAH) Investment Framework. 2016.

21 Health Policy Initiative. FP/RH Access for the Poor in Kenya: EQUITY Framework Encourages Policy and Financing Reforms. 2010.

22 Health Policy Initiative. The cost of family planning in Kenya. Washington DC: : Futures Group 2010.

23 Kenya National Bureau of Statistics, Ministry of Health, National AIDS Control Council, *et al.* Kenya Demographic and Health Survey 2014. Nairobi, Kenya and Rockville, MD: : ICF International 2015.

24 Hubacher D, Trussell J. A definition of modern contraceptive methods. *Contraception* 2015;**92**:420–1. doi:10.1016/j.contraception.2015.08.008

25 Rutstein SO, Johnson K. The DHS Wealth Index. Calverton, Maryland: 2004.

26 Filzmoser P, Gussenbauer J, Templ M. Detecting outliers in household consumption survey data. Vienna, Austria: 2016.

27 XE Currency Charts: USD to KES. http://www.xe.com/currencycharts/?from=USD&to=KES&view=5Y (accessed 5 Dec 2016).

28 Skordis-Worrall J, Pace N, Bapat U, *et al.* Maternal and neonatal health expenditure in Mumbai slums (India): a cross sectional study. *BMC Public Health* 2011;**11**:150.

For peer review only - http://bmjopen.bmj.com/site/about/guidelines.xhtml

doi:10.1186/1471-2458-11-150

29 Stata. testnl — Test nonlinear hypotheses after estimation. https://www.stata.com/manuals13/rtestnl.pdf (accessed 11 Jan 2018).

30 Chakraborty NM, Wanderi J, Oduor C, *et al.* Assessing provision and equity in low and middle income country health markets: a study from Kenya. Oakland, CA: 2017.

31 Arur A, Gitonga N, O'Hanlon B, *et al.* Insights from innovations: Lessons from designing and implementing family planning / reproductive health voucher programs in Kenya and Uganda. Bethesda, MD: 2009.

32 Njuki R, Obare F, Warren C, *et al.* Community experiences and perceptions of reproductive health vouchers in Kenya. *BMC Public Health* 2013;**13**:660. doi:10.1186/1471-2458-13-660

33 Barker C, Mulaki A, Mwai D, *et al.* Assessing county health system readiness in Kenya: A review of selected health inputs. Washington D.C.: 2014.

34 Wong KLM, Restrepo-Méndez MC, Barros AJD, *et al.* Socioeconomic inequalities in skilled birth attendance and child stunting in selected low and middle income countries: Wealth quintiles or deciles? *PLoS One* 2017;**12**:e0174823. doi:10.1371/journal.pone.0174823

35 Mwabu G, Mwanzia J, Liambila W. User charges in government health facilities in Kenya: effect on attendance and revenue. *Health Policy Plan* 1995;**10**:164–70. doi:10.1093/heapol/10.2.164

For peer review only

For peer review only - http://bmjopen.bmj.com/site/about/guidelines.xhtml

FIGURE 1: METHOD MIX AND PROVIDER USE BY WEALTH QUINTILE AMONG CURRENT MODERN FP USERS

For peer review only

For peer review only - http://bmjopen.bmj.com/site/about/guidelines.xhtml

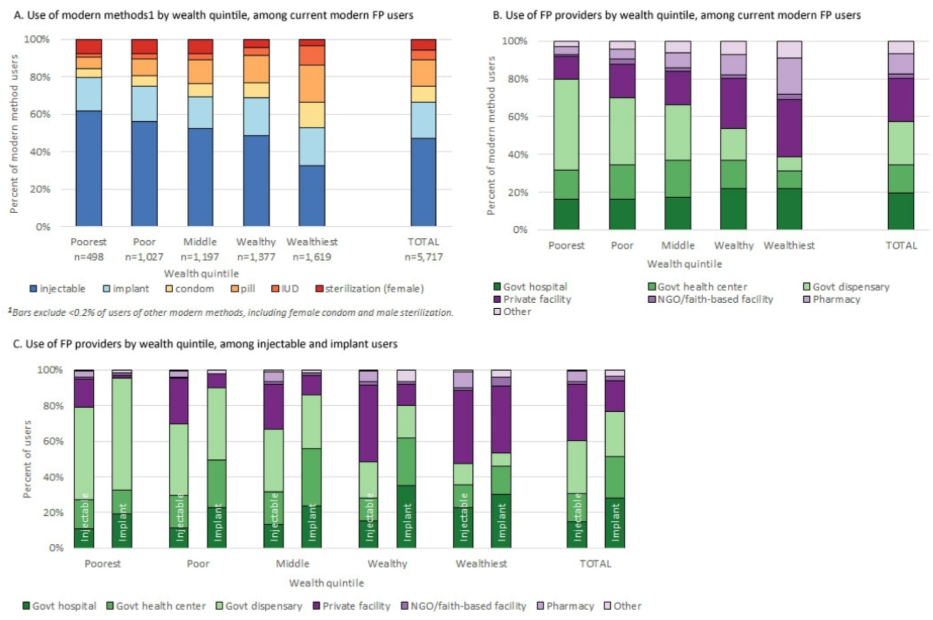

Method mix and provider use by wealth quintile among modern FP users

250x156mm (300 x 300 DPI)

Supplementary Table 1 Summary of out-of-pocket payment (in USD) for injectable and implant users among users with non-zero expenditure, by most recent provider of the contraceptive method, with different methods for dealing with outliers.

a. Observations greater than two standard deviations from the mean recoded as equal to the mean.

	Govt hospital	Govt health centre	Govt dispensary	TOTAL PUBLIC	Private facility	NGO/faith-based facility	Pharmacy/ chemist	Other[1]	TOTAL
Injectable									
n	247	225	490	962	821	28	148	17	1,976
Mean cost in USD	$0.83	$0.78	$0.74	$0.75	$1.07	$1.01	$1.08	$1.06	$0.91
SD	0.412	0.399	0.385	0.343	0.277	0.560	0.278	0.280	0.349
25th percentile	$0.57	$0.57	$0.57	$0.57	$0.91	$0.57	$0.91	$1.14	$0.57
50th percentile (median)	$0.57	$0.57	$0.57	$0.57	$1.14	$0.80	$1.14	$1.14	$1.14
75th percentile	$1.14	$1.14	$1.14	$1.14	$1.14	$1.14	$1.14	$1.14	$1.14
Implant									
n	136	94	102	332	130	11	-	3	477
Mean cost in USD	$3.48	$2.90	$2.37	$2.97	$7.47	$6.43		$6.20	$4.31
SD	3.363	2.525	1.629	2.724	5.034	4.430		6.090	4.096
25th percentile	$2.28	$1.14	$1.14	$1.14	$3.42	$2.28		$1.14	$2.28
50th percentile (median)	$2.28	$2.28	$2.28	$2.28	$5.74	$9.12		$1.14	$2.28
75th percentile	$3.42	$3.42	$2.28	$3.42	$11.40	$9.12		$1.14	$5.70

b. Observations greater than two standard deviations from the mean recoded to missing.

	Govt hospital	Govt health centre	Govt dispensary	TOTAL PUBLIC	Private facility	NGO/faith-based facility	Pharmacy/ chemist	Other[1]	TOTAL
Injectable									
n	237	216	476	929	794	21	144	17	1,905
Mean cost in USD	$0.81	$0.74	$0.71	$0.74	$1.07	$0.82	$1.09	$1.06	$0.91
SD	0.387	0.327	0.330	0.346	0.282	0.338	0.282	0.280	0.355
25th percentile	$0.57	$0.57	$0.57	$0.57	$0.91	$0.57	$0.91	$1.14	$0.57
50th percentile (median)	$0.57	$0.57	$0.57	$0.57	$1.14	$0.80	$1.14	$1.14	$1.14
75th percentile	$1.14	$1.14	$1.14	$1.14	$1.14	$1.14	$1.14	$1.14	$1.14
Implant									
n	135	94	102	331	109	11	-	3	454
Mean cost in USD	$3.47	$2.90	$2.37	$2.97	$7.81	$6.43	-	$6.20	$4.24
SD	3.367	2.525	1.629	2.724	5.454	4.430	-	6.090	4.183
25th percentile	$2.28	$1.14	$1.14	$1.14	$2.85	$2.28	-	$1.14	$2.28
50th percentile (median)	$2.28	$2.28	$2.28	$2.28	$5.70	$9.12	-	$1.14	$2.28
75th percentile	$3.42	$3.42	$2.28	$3.42	$11.40	$9.12	-	$11.40	$5.70

c. Observations greater than two standard deviations from the mean recoded as equal to the value two standard deviations from the mean.

	Govt hospital	Govt health centre	Govt dispensary	TOTAL PUBLIC	Private facility	NGO/faith-based facility	Pharmacy/ chemist	Other[1]	TOTAL
Injectable									
n	247	225	490	962	821	28	148	17	1,976
Mean cost in USD	$0.86	$0.80	$0.75	$0.79	$1.11	$1.13	$1.11	$1.06	$0.95
SD	0.454	0.419	0.396	0.419	0.331	0.628	0.319	0.280	0.412
25th percentile	$0.57	$0.57	$0.57	$0.57	$0.91	$0.57	$0.91	$1.14	$0.57
50th percentile (median)	$0.57	$0.57	$0.57	$0.57	$1.14	$0.80	$1.14	$1.14	$1.14
75th percentile	$1.14	$1.14	$1.14	$1.14	$1.14	$1.71	$1.14	$1.14	$1.14
Implant									
n	136	94	102	332	130	11	-	3	477
Mean cost in USD	$3.54	$2.90	$2.37	$3.00	$10.16	$6.43	-	$6.20	$5.06
SD	3.559	2.525	1.629	2.829	7.236	4.430	-	6.090	5.528
25th percentile	$2.28	$1.14	$1.14	$1.14	$3.42	$2.28	-	$1.14	$2.28
50th percentile (median)	$2.28	$2.28	$2.28	$2.28	$6.84	$9.12	-	$1.14	$2.28
75th percentile	$3.42	$3.42	$2.28	$3.42	$17.10	$9.12	-	$11.40	$5.70

d. Observations greater than 1.5 times the interquartile range recoded to missing.

	Govt hospital	Govt health centre	Govt dispensary	TOTAL PUBLIC	Private facility	NGO/faith-based facility	Pharmacy/ chemist	Other[1]	TOTAL
Injectable									
n	237	216	476	929	794	21	144	17	1,905
Mean cost in USD	$0.81	$0.74	$0.71	$0.74	$1.07	$0.82	$1.09	$1.06	$0.91
SD	0.387	0.327	0.330	0.346	0.282	0.338	0.282	0.280	0.355
25th percentile	$0.57	0.57	0.57	$0.57	0.912	0.57	0.912	1.14	$0.57
50th percentile (median)	$0.57	0.57	0.57	$0.57	1.14	0.798	1.14	1.14	$1.14
75th percentile	$1.14	1.14	1.14	$1.14	1.14	1.14	1.14	1.14	$1.14
Implant									
n	128	89	102	319	72	9	-	2	401
Mean cost in USD	$2.80	$2.43	$2.37	$2.56	$4.33	$5.12	-	$1.14	$2.93
SD	1.738	1.565	1.629	1.663	2.060	3.647	-	0.000	1.948
25th percentile	$2.28	$1.14	$1.14	$1.14	$2.28	$2.28	-	$1.14	$1.71
50th percentile (median)	$2.28	$2.28	$2.28	$2.28	$3.42	$2.28	-	$1.14	$2.28
75th percentile	$3.42	$2.85	$2.28	$3.42	$5.70	$9.12	-	$1.14	$3.42

e. Observations greater than 1.5 times the interquartile range recoded as equal to the value 1.5 times the interquartile range.

	Govt hospital	Govt health centre	Govt dispensary	TOTAL PUBLIC	Private facility	NGO/faith-based facility	Pharmacy/ chemist	Other[1]	TOTAL
Injectable									
n	247	225	490	962	821	28	148	17	1,976
Mean cost in USD	$0.86	$0.80	$0.75	$0.79	$1.10	$1.11	$1.11	$1.06	$0.95
SD	0.445	0.408	0.388	0.410	0.323	0.595	0.313	0.280	0.403
25th percentile	$0.57	$0.57	$0.57	$0.57	$0.91	$0.57	$0.91	$1.14	$0.57
50th percentile (median)	$0.57	$0.57	$0.57	$0.57	$1.14	$0.80	$1.14	$1.14	$1.14
75th percentile	$1.14	$1.14	$1.14	$1.14	$1.14	$1.71	$1.14	$1.14	$1.14
Implant									
n	136	94	102	332	130	11	-	3	477
Mean cost in USD	$3.25	$2.87	$2.37	$2.87	$7.27	$6.14	-	$5.92	$4.18
SD	2.515	2.424	1.629	2.274	3.583	3.994	-	5.751	3.394
25th percentile	$2.28	$1.14	$1.14	$1.14	$3.42	$2.28	-	$1.14	$2.28
50th percentile (median)	$2.28	$2.28	$2.28	$2.28	$6.84	$9.12	-	$1.14	$2.28
75th percentile	$3.42	$3.42	$2.28	$3.42	$10.83	$9.12	-	$10.83	$5.70

[1] Includes DHS response options: mobile clinic and other private medical

SD: Standard deviation

For peer review only

Supplementary Table 2 Out-of-pocket payment (in USD) for injectable and implant across all provider types among users with non-zero expenditure by socio–demographic characteristics.

	Injectable				*Implant*			
	n	Median	Mean (95% CI)		n	Median	Mean (95% CI)	
Wealth quintile								
Poorest	209	$0.80	$0.84 (0.77-0.91)		29	$2.28	$3.35 (1.88-4.81)	
Poor	417	$0.80	$0.82 (0.78-0.86)		89	$2.28	$2.78 (2.43-3.13)	
Middle	459	$0.80	$0.89 (0.84-0.93)	p<0.001	81	$2.28	$3.03 (2.54-3.53)	p<0.001
Wealthy	519	$1.14	$0.97 (0.93-1.01)		101	$2.28	$4.06 (2.83-5.30)	
Wealthiest	379	$1.14	$1.14 (1.08-1.21)		177	$5.70	$5.95 (4.74-7.17)	
Residence								
Urban	792	$1.14	$1.07 (1.03-1.11)	p<0.001	230	$3.42	$5.18 (4.15-6.21)	p=0.005
Rural	1191	$0.80	$0.85 (0.82-0.88)		246	$2.28	$3.49 (2.94-4.04)	
Educational attainment								
Less than primary	615	$0.91	$0.87 (0.84-0.91)		115	$2.28	$3.88 (2.90-4.85)	
Less than secondary	915	$1.14	$0.94 (0.90-0.98)	p<0.001	202	$2.28	$3.36 (2.78-3.94)	p=0.004
Secondary+	453	$1.14	$1.03 (0.98-1.08)		160	$3.42	$5.81 (4.49-7.14)	
Age group								
<20 years	77	$1.14	$0.92 (0.85-1.00)		9	$4.56	$3.50 (1.95-5.04)	
20–29 years	1032	$1.14	$0.94 (0.90-0.98)	p=0.897	226	$2.28	$4.20 (3.47-4.93)	p=0.594
30+ years	874	$1.14	$0.94 (0.90-0.97)		242	$2.28	$4.44 (3.50-5.38)	
Region†								
Central	207	$1.14	$1.07 (1.01-1.14)		87	$3.42	$4.51 (3.47-5.56)	
Coast	125	$1.14	$0.99 (0.87-1.11)		11	$2.28	$4.32 (1.35-7.29)	
Eastern	425	$0.91	$0.91 (0.85-0.96)		67	$3.42	$4.72 (3.79-5.64)	
Nairobi	183	$1.14	$1.18 (1.05-1.30)	p<0.001	51	$5.74	$8.03 (4.27-11.78)	p<0.001
Nyanza	315	$0.57	$0.84 (0.79-0.90)		66	$2.28	$2.90 (2.11-3.70)	
Rift Valley	495	$1.14	$0.93 (0.89-0.98)		129	$2.28	$4.08 (3.13-5.02)	
Western	232	$0.80	$0.81 (0.75-0.87)		64	$2.28	$2.58 (2.08-3.07)	

†Due to the very low modern contraceptive prevalence (<5%), results for the North Eastern region are not presented.

For peer review only

Supplementary Table 3 Proportion reporting free family planning by provider among users initiating their long–acting contraceptive method before or after June 2013 abolition of fees.

	Govt hospital % (95% CI)	Govt health centre % (95% CI)	Govt dispensary % (95% CI)	TOTAL PUBLIC % (95% CI)
Initiated before June 2013				
IUD (n=898)	49.9 (36.6–63.2)	78.8 (59.5–90.4)	76.9 (56.4–89.6)	61.2 (51.5–70.1)
Implant (n=469)	51.3 (40.6–61.9)	62.7 (51.5–72.7)	57.8 (48.3–66.7)	56.6 (50.4–62.7)
Initiated from June 2013 onward				
IUD (n=48)	48.5 (26.4–71.2)	69.2 (41.6–87.6)	64.6 (32.1–87.6)	59.2 (42.8–73.7)
Implant (n=357)	61.2 (50.5–70.9)	63.2 (50.0–74.7)	65.6 (56.0–74.0)	63.4 (56.9–69.5)

For peer review only

BMJ Open

Who pays and how much? A cross-sectional study of out-of-pocket payment for modern contraception in Kenya

Journal:	*BMJ Open*
Manuscript ID	bmjopen-2018-022414.R1
Article Type:	Research
Date Submitted by the Author:	12-Jul-2018
Complete List of Authors:	Radovich, Emma; London School of Hygiene and Tropical Medicine, Faculty of Epidemiology & Population Health<br>Dennis, Mardieh; London School of Hygiene and Tropical Medicine, Faculty of Epidemiology & Population Health<br>Barasa, Edwine; KEMRI-Wellcome Trust Research Programme, Health Economics Research Unit<br>Cavallaro, Francesca; London School of Hygiene and Tropical Medicine, Faculty of Epidemiology & Population Health<br>Wong, Kerry; London School of Hygiene and Tropical Medicine, Faculty of Epidemiology & Population Health<br>Borghi, Josephine; London School of Hygiene and Tropical Medicine, Faculty of Public Health & Policy<br>Lynch, Caroline; London School of Hygiene and Tropical Medicine, Faculty of Epidemiology & Population Health<br>Lyons-Amos, Mark; London School of Hygiene and Tropical Medicine, Faculty of Epidemiology & Population Health<br>Abuya, Timothy; Population Council Kenya<br>Benova, Lenka; London School of Hygiene and Tropical Medicine, Faculty of Epidemiology & Population Health; Instituut voor Tropische Geneeskunde, Department of Public Health
**Primary Subject Heading**:	Global health
Secondary Subject Heading:	Health services research
Keywords:	Kenya, family planning, user fees, Demographic & Health Survey, private sector, government provision

SCHOLARONE™
Manuscripts

Who pays and how much? A cross-sectional study of out-of-pocket payment for modern contraception in Kenya

Authors: Emma Radovich[1], Mardieh L. Dennis[1], Edwine Barasa[3], Francesca Cavallaro[1], Kerry L.M. Wong[1], Josephine Borghi[2], Caroline A. Lynch[1], Mark Lyons-Amos[1], Timothy Abuya[4], and Lenka Benova[1,5]

[1] Faculty of Epidemiology and Population Health, London School of Hygiene & Tropical Medicine, London, UK
[2] Faculty of Public Health and Policy, London School of Hygiene & Tropical Medicine, London, UK
[3] Health Economics Research Unit, KEMRI-Wellcome Trust Research Programme, Nairobi, Kenya
[4] Population Council, Nairobi, Kenya
[5] Department of Public Health, Institute of Tropical Medicine, Antwerp, Belgium

Corresponding author:
Emma Radovich, London School of Hygiene & Tropical Medicine
Keppel Street
London WC1E 7HT
United Kingdom
+44(0)20 7612 7922
emma.radovich@lshtm.ac.uk

Word count: 4,302

Keywords: Kenya; family planning; user fees; Demographic & Health Survey; private sector; government provision

For peer review only

ABSTRACT (300 words)

Objectives: Out-of-pocket (OOP) payment for modern contraception is an under-studied component of healthcare financing in countries like Kenya, where wealth gradients in met need have prompted efforts to expand access to free contraception. This study aims to examine whether, among public sector providers, the poor are more likely to receive free contraception and to compare how OOP payment for injectables and implants—two popular methods—differs by public/private provider type and user's socio-demographic characteristics.

Design, setting and participants: Secondary analyses of nationally representative, cross-sectional household data from the 2014 Kenya Demographic and Health Survey. Respondents were women of reproductive age (15-49 years). The sample comprised 5,717 current modern contraception users, including 2,691 injectable and 1,073 implant users with non-missing expenditure values.

Main outcome: Respondent's self-reported source and payment to obtain their current modern contraceptive method.

Methods: We used multivariable logistic regression to examine predictors of free public sector contraception and compared average expenditure for injectable and implant. Quintile ratios examined progressivity of non-zero expenditure by wealth.

Results: Half of public sector users reported free contraception; this varied considerably by method and region. Users of implants, condoms, pills and IUDs were all more likely to report receiving their method for free ($p<0.001$) compared to injectable users. The poorest were as likely to pay for contraception as the wealthiest users at public providers (OR: 1.10, 95%CI: 0.64-1.91). Across all providers, among users with non-zero expenditure, injectable and implant users reported a mean OOP payment of KES 80 (US $0.91), 95%CI: KES 78-82 and KES 378 (US $4.31), 95%CI: KES 327-429, respectively. In the public sector, expenditure was pro-poor for injectable users yet weakly pro-rich for implant users.

Conclusions: More attention is needed to targeting subsidies to the poorest and ensuring government facilities are equipped to cope with lost user fee revenue.

ARTICLE SUMMARY

Strengths and limitations of this study

- A major strength of the study is that it is the first to our knowledge to use nationally representative data from a low-income country to examine out-of-pocket payment for modern contraception.
- Another strength is the transparency in the classification of family planning providers, handling of outliers and appropriate adjustments for complex survey design.
- One limitation of the study is the reliance on self-reported cost data from current users of modern contraception and the inability to compare this with costs to women who discontinued or eschewed use of modern contraception.

For peer review only - http://bmjopen.bmj.com/site/about/guidelines.xhtml

BACKGROUND

Achieving Universal Health Coverage—including for family planning (FP) services—demands attention to financial protection and whether the inability to pay restricts individuals from accessing needed healthcare. Consideration of user fees is particularly important in countries like Kenya, where out-of-pocket (OOP) payments form a substantial proportion of healthcare financing[1,2]. In Kenya, unmet need for FP is highest among the poor, with a documented 8-14 percentage point increase in modern contraception use with each increase in household wealth quintile[3]. A study in Kenya and India found that poor households spend a significantly higher proportion of their income on reproductive health care (including FP), with the poorest households in Kenya spending 10 times the proportion spent by the least poor[4]. Many government financial protection policies focus on inpatient events where healthcare expenditure is likely to be catastrophic, yet the greater frequency of outpatient expenses—including for contraceptive services, which affect women in particular—can also push households into poverty[5] or reduce care-seeking among the poor[3,6].

A systematic review on the relationship between user fees and FP use in low- and middle-income countries (LMICs) was inconclusive, though some included studies suggested that young people and the poor were more sensitive to price increases than wealthier or less marginalised groups[7]. Cost is rarely cited as the reason for non-use of modern contraception among women in need (those wishing to delay or avoid pregnancy) in Demographic and Health Surveys (DHS) [8,9]. Yet, focus groups in Nyanza Province, Kenya found that the poor identify high cost of services as a barrier to FP care, both in opportunity costs associated with seeking care and direct fees for services[3], suggesting that for some individuals, cost can impact FP access.

Kenya has used various financing mechanisms to support increased access to FP and reproductive health services[3,10]. A 2004 policy, commonly known as the "10/20 policy", abolished user fees in primary care facilities; instead government dispensaries and health centres were allowed to charge a registration fee of 10 or 20 Kenyan shillings (KES) (approximately US $0.11 and $0.23), with the poor exempted from payment[11,12]. Public hospitals could continue charging fees to users under a cost sharing policy. Yet fee waiver implementation and identification of eligible individuals was left to the discretion of actors at the community and facility-level. Despite the 10/20 policy, many FP clients in government facilities reported paying additional 'hidden fees' for the consultation, medical tests or equipment, and the contraceptive commodity[3]. A 2010 health facility survey found that approximately 70% of government facilities providing FP charged user fees for services[13]. A 2009 study found low community knowledge of the 10/20 policy and qualifying exemptions[11]. However, as of June 2013, all fees at public outpatient primary care facilities (dispensaries and health centres) were eliminated[12], and FP services are intended to be provided for free at public facilities[14,15]. The extent to which users currently receive free FP services from public outpatient primary care facilities is unknown, and similar to the 10/20 policy, implementation of the June 2013 policy for free FP services may vary, for example by facility type, geographic region or client characteristics.

Efforts to achieve universal coverage for reproductive health have led to increasing calls by donors and others for a "total market approach" in considering the different contributions of public and private providers. In this approach, government- or otherwise-subsidised services are targeted to meet the needs of the poor while individuals with the ability to pay are indirectly encouraged to seek FP services from commercial or unsubsidised private providers[3,5,16,17]. Kenya's changing fee policies within the public sector and the country's growing private sector, which now owns half of all health facilities[12], raise questions about where individuals, especially the poor, seek FP and what this means for their OOP payment for modern contraception. Little is known about OOP payment to obtain modern contraception in sub-Saharan Africa, and in Kenya in particular, and how this varies by provider type. In the context of limited resources to expand FP access[18], it is important to understand the burden of user fees—who pays and

For peer review only - http://bmjopen.bmj.com/site/about/guidelines.xhtml

how much—and the degree to which vulnerable groups are served by current efforts to provide affordable care.

This paper aims to address these knowledge gaps by describing FP sources by user's wealth in Kenya, examining, among public sector users, who receives free FP services, and comparing how payment for injectables and implants—the two most commonly used methods—differs by FP provider type and the user's socio-demographic characteristics.

METHODS

Data source

We used data from the most recent Kenya DHS (2014), a nationally representative, cross-sectional household survey of women age 15-49 with a multi-level cluster sampling design. A detailed description of the survey sampling can be found in the Kenya 2014 DHS report[19]. Interviews were administered between May and October 2014. Women in a random sample of half of the households in the Kenya 2014 DHS were administered a short-version Woman's Questionnaire[19], which did not ask respondents for the amount paid for their current contraceptive method. We include in our analysis only women in the other half of randomly selected households who were administered the long-version Woman's Questionnaire; the long-version questionnaire included a question on the amount paid for the respondent's current contraceptive method. Respondents were not asked for the reason for the payment.

Study populations

We examined data from three populations of women: 1) current users of modern contraception, based on the Hubacher & Trussell definition of modern methods[20]; 2) users of IUD, implant, injectable, pill and male condom as these users were asked to self-report the total amount paid to obtain their method (the combined cost of the commodity and any consultation fees) during their most recent (re-)supply visit; and 3) users of injectable and implant, where estimates of OOP payment refer to a single quantity of the contraceptive, as users can receive only one "dose" during insertion or re-supply. Respondents with missing or "don't know" expenditure values accounted for 4.4% of all users in group two, and less than 1% of injectable and implant users, and were excluded from analysis.

Definitions

We classified women's self-reported most recent source of modern FP into seven provider categories: 1) government hospital; 2) government health centre; 3) government dispensary; 4) private facility, a constructed category comprising DHS response options of private hospital/clinic and private nursing/maternity home; 5) NGO/faith-based facility; 6) pharmacy/chemist; and 7) other, a constructed category of the response options: shop, mobile clinic, friend/relative, other, community health worker, community-based distributor and other private medical. We defined the public sector to be government-provided services (categories 1-3) and non-public providers to be categories 4-7. We consider public primary care providers to be categories 2 and 3. Less than 1% of all current modern contraceptive users were missing the source of their method and were excluded from analysis.

We examined three measures of the respondents' socio-economic status: household wealth quintiles derived by the DHS from household assets[21], urban/rural residence, and three levels of educational attainment: less than primary school (respondents with no education and those who started but did not complete primary school), less than secondary school (respondents with complete primary or incomplete secondary school) and secondary+ (respondents with complete secondary or some higher education). We used DHS categories for respondent's current marital status (never, currently or formerly in union) and grouped respondents by their current age: less than 20, 20-29 and 30+ years. Kenya is administratively divided into 47 counties; however the variable for OOP payment for contraception on the 2014 Kenya DHS

For peer review only - http://bmjopen.bmj.com/site/about/guidelines.xhtml

was intended to provide representative estimates for the national level, for urban and rural areas, and for the eight regions (former provinces)[19].

Analysis of free or 'registration fee only' FP in the public sector

We limited analysis of free FP to users whose most recent source of the method was a public sector provider. We include both public primary care providers (subject to the June 2013 abolishment of fees) and government hospitals as a point of comparison. Adjusted Wald tests were performed to compare proportions reporting free FP by facility type and user characteristics. Bivariable and multivariable logistic regression was used to examine predictors, such as wealth quintile, facility type and region, of receiving free FP from public primary care facilities, as indicated under the 2013 policy.

Users of long-acting methods like IUD and implant could report OOP payment based on FP consultations before the June 2013 abolishment of fees. Thus, estimates of free FP for IUD and implant were further disaggregated by whether the method was obtained before or after June 2013 based on the respondent's self-reported month and year of initiating use of the method.

Facility-level implementation of the June 2013 policy abolishing all fees at public primary care facilities may not have been immediate. As such, we additionally examined the proportion of users who reported paying up to 10 KES or 20 KES at a government dispensary or health centre, respectively, referring to these users as paying 'registration fees only' consistent with the former 10/20 policy, though respondents did not indicate the reason for the charge.

Analysis of OOP payment for injectable and implant

Prior to analysis, we assessed the data for improbable values and recoded observations to missing if reported expenditure was greater than 10 times the 95th percentile (six observations). Among injectable and implant users reporting non-zero cost, we described the patterns of OOP expenditure, reporting mean and median values. We conducted sensitivity analyses to ensure the robustness of our results, comparing results from multiple methods for dealing with outliers[22]; results did not differ substantially (Supplementary Table 1). For this analysis, observations greater than two standard deviations (SD) from the mean (2.7% and 2.1% of injectable and implant users, respectively) were recoded to be equal to the mean. Simple linear regression and marginal effects were used to compare means between providers and user characteristics. We additionally present estimates of OOP payment converted from KES to US dollars (USD) based on 1 KES to 0.0114 USD conversion rate for the midpoint of fieldwork in July 2014[23] in Supplementary Tables 1-2.

Equity of OOP payment for injectable and implant

Quintile ratios were used to measure the progressiveness of OOP payments for injectables and implants overall and within the public sector. This measure of equity in expenditure assumes that individuals in the lowest wealth quintile have less capacity to pay and thus if they spend the same or more as those in the highest quintile, this represents a greater proportion of income and constitutes regressive spending[24]. Quintile ratios were calculated by comparing mean expenditure in the wealthiest and poorest wealth quintiles and testing for differences in using an adjusted Wald-type test of nonlinear hypotheses based on the delta method, attributing significance at a 95% confidence level[4,24,25]. We define expenditure as weakly pro-rich if there was no significant difference in mean payment between the poorest and wealthiest users and strongly pro-rich if the poorest users paid significantly more than the wealthiest users (quintile ratio <1)[4,24].

All analysis used women's individual sampling weights and standard error adjustment to account for complex survey design. Analyses were conducted in Stata/SE v14 (StataCorp, College Station, TX, USA).

For peer review only - http://bmjopen.bmj.com/site/about/guidelines.xhtml

RESULTS

A total of 5,717 modern contraceptive users with non-missing provider data were included in our analysis sample.

Methods and sources of family planning

Among all current FP users, the wealthiest quintile had the broadest mix of methods, with no single method accounting for more than a third of current modern FP users (Figure 1a). In contrast, method mix among users in the three poorer quintiles was dominated by injectables, which accounted for more than half of methods used. While injectables and implants were the two most popular methods for all users, this was particularly true for the poorest users, where these two methods accounted for nearly 80% of all modern methods used.

The wealthiest contraceptive users also reported a broader mix of providers (Figure 1b). Among the poorest users, 80.0% reported a public sector source. Public provider use decreased steadily and use of private facilities and pharmacies increased with increasing wealth quintile. The wealthiest users reported the largest use of private facilities (30.5%) and pharmacies (18.7%). Among injectable users, public sector providers were the most-used source for the three poorest quintiles, with a clear decline in government dispensary use with increasing wealth (Figure 1c). The vast majority of implants in the four poorer wealth quintiles were sourced from public providers, and there was a dramatic increase in use of private facilities for implants in the fifth, wealthiest quintile.

Free family planning

Overall, 51.1% of public sector users reported obtaining their modern FP method for free at their most recent visit (Table 1). This varied by method used: more than 90% of condom users compared to 40.7% of injectable users reported free FP. Across the three levels of facilities, 50.1% of government hospital, 56.2% of government health centre, and 48.5% of government dispensary users reported free FP, with some evidence of a difference by facility type (p=0.048). The percentage of women obtaining free FP in public facilities differed only slightly by respondent's wealth quintile, urban/rural residence, education level, or age group. The proportion of users reporting free FP varied considerably by region, with 39.4% of Rift Valley residents compared to 76.6% of Nairobi residents reporting free contraception. Additionally, 1.3% (95% CI: 0.9-2.1%) of users of government health centres and dispensaries reported paying a 'registration fee only' amount under the former 10/20 policy (results not shown). There was no difference by user's wealth quintile.

For peer review only - http://bmjopen.bmj.com/site/about/guidelines.xhtml

Table 1: Among public sector providers, proportion reporting free family planning by modern method users' socio–demographic characteristics

	Govt hospital (n=929)	Govt health centre (n=815)	Govt dispensary (n=1,267)	TOTAL PUBLIC (n=3,011)
Overall (95% CI)	50.1 (45.9–54.3)	56.2 (50.9–61.4)	48.5 (45.0–52.1)	51.1 (48.5–53.7)
Method				
Injectable	38.4 (32.4–44.8)	46.0 (39.3–52.9)	39.2 (34.8–43.7)	40.7 (37.5–44.1)
Implant	55.0 (47.1–62.6)	63.0 (53.9–71.1)	61.5 (54.6–67.9)	59.6 (54.8–64.2)
Pill	68.7 (53.8–80.6)	66.0 (50.6–78.6)	61.1 (50.0–71.3)	64.7 (57.1–71.6)
Condom	90.8 (71.0–97.5)	92.8 (77.4–98.0)	97.4 (83.7–99.6)	93.6 (85.0–97.3)
IUD	49.6 (38.6–60.7)	75.4 (57.8–87.3)	73.4 (57.1–85.1)	60.7 (52.8–68.1)
Wealth quintile				
Poorest	61.7 (48.7–73.1)	54.1 (41.3–66.5)	46.1 (39.2–53.1)	50.2 (44.4–55.9)
Poor	51.2 (41.0–61.4)	51.7 (42.2–61.1)	44.3 (38.4–50.4)	47.6 (43.0–52.3)
Middle	43.2 (35.5–51.2)	52.4 (42.6–62.0)	48.9 (41.3–56.5)	48.6 (43.6–53.7)
Wealthy	51.8 (43.6–60.0)	57.1 (47.3–66.3)	54.4 (45.9–62.7)	54.2 (48.6–59.7)
Wealthiest	49.6 (42.0–57.2)	66.9 (55.0–76.9)	53.9 (39.8–67.4)	54.9 (48.7–61.0)
Residence				
Urban	49.1 (43.6–54.7)	66.0 (56.8–74.2)	56.1 (47.6–64.3)	55.2 (50.6–59.7)
Rural	51.6 (45.1–58.0)	50.9 (44.7–57.0)	46.8 (42.9–50.7)	48.8 (45.8–51.9)
Educational attainment				
Less than primary	56.7 (48.3–64.7)	55.1 (47.0–62.9)	47.6 (42.5–52.6)	51.7 (47.8–55.6)
Less than secondary	46.0 (40.0–52.0)	56.6 (49.3–63.6)	48.9 (43.8–53.9)	49.9 (46.4–53.5)
Secondary+	51.3 (43.0–59.4)	57.1 (46.9–66.6)	49.8 (40.9–58.7)	52.5 (47.0–58.0)
Age group				
<20 years	41.3 (19.4–67.4)	61.3 (40.2–78.9)	60.4 (42.8–75.7)	55.4 (43.4–66.9)
20–29 years	45.8 (40.0–51.7)	55.3 (48.1–62.4)	42.9 (37.9–48.1)	47.0 (43.5–50.6)
30+ years	54.2 (48.0–60.3)	56.5 (49.8–63.0)	52.7 (47.8–57.5)	54.2 (50.7–57.7)
*Region**				
Central	53.1 (43.5–62.4)	64.0 (50.7–75.4)	60.2 (47.8–71.4)	58.6 (51.3–65.5)
Coast	70.3 (58.9–79.7)	81.2 (69.6–89.0)	62.4 (52.4–71.4)	69.1 (62.1–75.2)
Eastern	35.2 (26.3–45.2)	40.4 (29.5–52.3)	44.5 (36.9–52.4)	41.5 (36.0–47.2)
Nairobi	70.4 (54.1–82.7)	76.0 (55.3–89.0)	–†	76.6 (63.4–86.0)
Nyanza	59.4 (49.8–68.3)	55.6 (44.2–66.4)	37.0 (30.0–44.7)	49.0 (43.1–55.0)
Rift Valley	30.9 (24.2–38.5)	48.0 (37.3–58.9)	42.8 (36.7–49.1)	39.4 (35.1–43.8)
Western	60.1 (43.6–74.5)	46.6 (34.2–59.4)	50.0 (39.8–60.3)	50.6 (43.5–57.7)

*Due to the very low modern contraceptive prevalence (<5%), results for the North Eastern region are not presented.

†No respondents reported this provider.

Among non-public sector providers (results not shown), 10.9% of private facility users and less than 1% of pharmacy users reported free FP. Of the limited number of users of NGO/faith-based facilities (n=91), 30.9% reported obtaining their contraceptive method for free.

Supplementary Table 3 shows the proportion of IUD and implant users receiving free FP from public sector providers among users initiating the method before and after the June 2013 fee abolishment. Among implant users, the proportion receiving free FP from government health centres was similar between the two initiation periods and increased in the later period among users of government hospitals and dispensaries, though confidence intervals overlap. Among IUD users, the proportion receiving free care was slightly lower across all three public provider categories in the later initiation period, but differences were not statistically significant.

For peer review only - http://bmjopen.bmj.com/site/about/guidelines.xhtml

Table 2 shows the results of bivariable and multivariable analysis of receiving free modern FP among public primary care facility users. There were no differences by wealth quintile in the odds of obtaining free contraception after adjusting for method, provider type and user characteristics. Users of implants, condoms, pills and IUDs were all more likely to report receiving their method for free (p<0.001) compared to injectable users, and this relationship remained after adjusting for provider and user characteristics. Users in all regions had lower odds of free contraception compared to Nairobi, except Coast region (where it was not significantly different).

Table 2: Unadjusted and adjusted odds ratios from logistic regression analysis of reporting free family planning services from government primary care providers among modern method users

Variables	*Modern method users utilising public primary care providers (n=2,079)*			
	Unadjusted		Adjusted	
	OR	(95% CI)	OR	(95% CI)
Wealth quintile				
Poorest	0.58*	(0.37-0.91)	1.10	(0.64-1.91)
Poor	0.55**	(0.36-0.85)	1.20	(0.71-2.03)
Middle	0.64*	(0.41-0.99)	1.25	(0.74-2.11)
Wealthy	0.79	(0.51-1.24)	1.16	(0.67-2.01)
Wealthiest	Ref		Ref	
Provider				
Govt. health centre	Ref		Ref	
Govt. dispensary	0.74*	(0.57-0.95)	0.95	(0.74-1.22)
Method				
injectable	Ref		Ref	
implant	2.32***	(1.78-3.02)	2.15***	(1.62-2.86)
condom	29.87***	(9.84-90.66)	35.29***	(11.42-109.05)
pill	2.39***	(1.63-3.52)	2.27***	1.56-3.28)
IUD	4.14***	(2.26-7.56)	3.90***	(2.06-7.36)
Residence				
Urban	Ref		Ref	
Rural	0.58***	(0.43-0.79)	0.83	(0.60-1.14)
Region				
Central	0.36*	(0.14-0.93)	0.31*	(0.10-0.93)
Coast	0.48	(0.18-1.24)	0.53	(0.19-1.53)
Eastern	0.17***	(0.07-0.42)	0.18**	(0.06-0.52)
Nairobi	Ref		Ref	
Nyanza	0.18***	(0.07-0.44)	0.17**	(0.06-0.50)
Rift Valley	0.17***	(0.07-0.43)	0.19**	(0.07-0.53)
Western	0.21**	(0.08-0.53)	0.21**	(0.07-0.62)
Age group				
<20 years	1.31	(0.75-2.28)	1.58	(0.85-2.92)
20–29 years	0.76*	(0.61-0.95)	0.85	(0.67-1.08)
30+ years	Ref		Ref	
Marital status				
Never in union	0.93	(0.62-1.40)	0.80	(0.47-1.36)
Currently in union	Ref		Ref	
Formerly in union	1.38†	(0.96-1.98)	1.27	(0.88-1.83)

Note: Asterisks and daggers indicate that differences between the category and the reference category are significant at *p<0.05, **p<0.01, ***p<0.001, or marginally significant at †p<0.1.

For peer review only

For peer review only - http://bmjopen.bmj.com/site/about/guidelines.xhtml

Out-of-pocket payment for injectables and implants

Among injectable and implant users reporting greater than zero OOP payment to obtain the method from their most recent provider (both sectors combined), the mean cost was KES 80 (US $0.91) (95% CI: KES 78-82) for injectable and KES 378 (US $4.31) (95% CI: KES 327-429) for implant (Table 3). 1.7% of injectable and 1.5% of implant users reported paying amounts consistent with registration fees only (<KES 20). OOP payment varied, particularly for implant, by source of the method, with some private facility users reporting very high costs. Injectable users of public sector providers reported a median cost of KES 50, whereas the median cost was twice that (KES 100) for those accessing private facilities or pharmacies. Among implant users, those accessing public sector sources reported a median cost of KES 200, compared to a median cost of KES 503 among those utilising private facilities.

When assessed by user characteristics, mean OOP payment for both injectables and implants varied significantly by user's wealth, residence, education level and region, but not by user's age (Table 4). Urban and Nairobi residents paid more for both methods; this was particularly notable for implant users in Nairobi, where mean cost was more than twice that of implant users in Western or Nyanza regions. Mean and median cost did not increase linearly with increasing wealth quintile. For injectable users, median cost in the poorest three quintiles was KES 70 compared to KES 100 in the two wealthiest quintiles. For implant users, median cost of KES 500 in the wealthiest quintile was more than twice the median cost of KES 200 in the four poorer quintiles. The overall quintile ratio for all providers comparing mean cost in the wealthiest quintile to the poorest quintile was 1.3 ($p<0.001$) for injectable and 1.8 ($p=0.007$) for implant, indicating strong evidence of pro-poor OOP payment for both methods. Among public sector users, the quintile ratio was 1.2 ($p=0.033$) for injectable indicating pro-poor expenditure, and 0.90 ($p=0.660$) for implant (Table 5), indicating weakly pro-rich expenditure (no difference in mean cost between the quintiles) for public sector implant users.

For peer review only

Table 3: Summary of out-of-pocket payment (in KES) for injectable and implant users among users with non-zero expenditure, by most recent provider of the contraceptive method

	Govt hospital	Govt health centre	Govt dispensary	TOTAL PUBLIC	Private facility	NGO/faith-based facility	Pharmacy/ chemist	Other[1]	TOTAL
Injectable									
n	247	225	490	962	821	28	148	17	1,976
Mean cost	KES 72	KES 66	KES 63	KES 66	KES 94	KES 75	KES 95	KES 93	KES 80
SD	33.38	28.37	28.78	30.10	24.33	26.37	24.42	24.56	30.63
25th percentile	KES 50	KES 50	KES 50	KES 50	KES 80	KES 50	KES 80	KES 100	KES 50
50th percentile (median)	KES 50	KES 50	KES 50	KES 50	KES 100	KES 70	KES 100	KES 100	KES 100
75th percentile	KES 100	KES 100	KES 100	KES 100	KES 100	KES 87	KES 100	KES 100	KES 100
Reporting registration fees only	7.5%	5.3%	0.8%	3.6%					1.7%
Implant									
n	136	94	102	332	130	11	-	3	477
Mean cost	KES 305	KES 255	KES 208	KES 261	KES 655	KES 564		KES 544	KES 378
SD	295.01	221.51	142.92	238.98	441.62	388.58		534.17	359.25
25th percentile	KES 200	KES 100	KES 100	KES 100	KES 300	KES 200		KES 100	KES 200
50th percentile (median)	KES 200	KES 200	KES 200	KES 200	KES 503	KES 800		KES 100	KES 200
75th percentile	KES 300	KES 300	KES 200	KES 300	KES 1,000	KES 800		KES 1,000	KES 500
Reporting registration fees only	1.7%	5.0%	0.0%	2.1%					1.5%

[1] Includes DHS response options: mobile clinic and other private medical

SD: Standard deviation 1 KES = 0.0114 USD

For peer review only

For peer review only - http://bmjopen.bmj.com/site/about/guidelines.xhtml

Table 4: Out-of-pocket payment (in KES) for injectable and implant across all provider types among users with non-zero expenditure by socio–demographic characteristics

	**Injectable**						**Implant**					
	n	Median	Mean	(95% CI)			n	Median	Mean	(95% CI)		
Wealth quintile						Quintile ratio†						Quintile ratio†
Poorest	209	KES 70	KES 71	(66-77)			29	KES 200	KES 294	(165-422)		
Poor	417	KES 70	KES 71	(67-74)			89	KES 200	KES 244	(212-274)		
Middle	459	KES 70	KES 76	(73-79)	p<0.001		81	KES 200	KES 266	(223-309)	p<0.001	
Wealthy	516	KES 100	KES 83	(80-87)			101	KES 200	KES 357	(248-465)		
Wealthiest	379	KES 100	KES 96	(91-101)		1.3 (p<0.001)	177	KES 500	KES 522	(415-629)		1.8 (p=0.007)
Residence												
Urban	790	KES 100	KES 91	(88-94)	p<0.001		230	KES 200	KES 455	(364-545)	p=0.005	
Rural	1191	KES 70	KES 73	(71-75)			246	KES 300	KES 306	(258-355)		
Educational attainment												
Less than primary	615	KES 80	KES 75	(72-78)			115	KES 200	KES 340	(255-425)		
Less than secondary	915	KES 100	KES 80	(77-84)	p<0.001		202	KES 200	KES 295	(244-346)	p=0.004	
Secondary+	451	KES 100	KES 87	(83-91)			160	KES 300	KES 510	(394-626)		
Age group												
<20 years	77	KES 100	KES 81	(74-87)			9	KES 400	KES 307	(171-442)		
20–29 years	1030	KES 87	KES 80	(77-83)	p=0.928		226	KES 200	KES 369	(304-433)	p=0.594	
30+ years	874	KES 100	KES 80	(77-82)			242	KES 200	KES 389	(307-472)		
*Region**												
Central	207	KES 100	KES 90	(86-95)			87	KES 300	KES 396	(304-488)		
Coast	125	KES 100	KES 82	(73-92)			11	KES 200	KES 379	(119-639)		
Eastern	425	KES 80	KES 77	(73-81)			67	KES 300	KES 414	(333-495)		
Nairobi	183	KES 100	KES 101	(91-111)	p<0.001		51	KES 503	KES 704	(374-1034)	p<0.001	
Nyanza	315	KES 50	KES 72	(68-77)			66	KES 200	KES 255	(185-324)		
Rift Valley	492	KES 87	KES 80	(76-84)			129	KES 200	KES 358	(275-440)		
Western	232	KES 70	KES 69	(64-74)			64	KES 200	KES 226	(183-270)		

*Due to the very low modern contraceptive prevalence (<5%), results for the North Eastern region are not presented.

†Ratio of mean OOP expenditure comparing the wealthiest users to the poorest users. Adjusted Wald-type test based on the delta method was used to test for significance.
1 KES = 0.0114 USD

For peer review only - http://bmjopen.bmj.com/site/about/guidelines.xhtml

Table 5: Out-of-pocket payment (in KES) for public sector injectable and implant among users with non-zero expenditure by wealth quintile

	Injectable				*Implant*			
	n	Mean	(95% CI)	Quintile ratio†	n	Mean	(95% CI)	Quintile ratio†
Wealth quintile								
Poorest	147	KES 65	(59-72)		27	KES 267	(146-389)	
Poor	247	KES 61	(57-65)		76	KES 231	(200-262)	
Middle	256	KES 66	(62-70)		68	KES 253	(205-301)	
Wealthy	197	KES 66	(61-72)		78	KES 317	(187-447)	
Wealthiest	116	KES 78	(69-87)	1.2 (p=0.033)	82	KES 240	(190-291)	0.90 (p=0.660)

†Ratio of mean OOP expenditure comparing the wealthiest users to the poorest users. Adjusted Wald-type test based on the delta method was used to test for significance.
1 KES = 0.0114 USD

For peer review only

DISCUSSION

This is the first study to our knowledge to use nationally representative household data from a LMIC to examine equity of OOP payment for FP, comparing differences in cost to users accessing public and non-public providers. The wealthiest FP users in Kenya utilised a greater mix of modern methods and providers compared to the poorest users, and use of non-public providers increased with increasing wealth. Despite Kenya's national policy to offer free FP services at public primary care facilities, we found only half of modern method users reported obtaining their method at no cost from government providers, with little variation by facility type. There were no differences by user's socio-economic position. Among injectable and implant users reporting OOP expenditure, there were considerable differences by source of the method. Consistent with a previous study of FP users in urban Kenya[26], we found private facility and pharmacy users, unsurprisingly, reported higher expenditures than users of public facilities. Unfortunately, due to very small sample sizes (<30 users), OOP payment by users of NGO/faith-based facilities remains unclear, though there is some indication that costs may be higher than among public sector providers. Greater use of higher cost, non-public providers by the wealthiest users contributed to overall pro-poor expenditure, with both injectable and implant users in the wealthiest quintile paying significantly more than their counterparts in the poorest quintile.

A 'total market approach' to FP includes efforts to target government subsidies to the poorest contraceptive users and indirectly nudge wealthier users to seek FP from non-public providers. Evidence from this study suggests that market forces appear to be working to encourage greater use of non-public providers by the wealthiest users, though nearly 40% of FP users in the wealthiest quintile still sourced their method from the public sector. However, while the poorest users obtained their methods overwhelmingly from public providers they were equally likely to pay for FP as users in the wealthiest quintile, suggesting the potential for better targeting of free services to ensure the national pro-poor strategy of removing user fees for FP in public primary care facilities is reaching recipients most in need.

The Kenyan government faces the challenge of both meeting targets to reduce unmet need for FP[14] and ensuring all women, including the poor, have choice in FP methods and providers. Recent attempts to expand access to long-term methods, like implants, in Kenya have focused on expanding the range of providers available to the poor through vouchers. In 2005, Kenya launched a pilot system in five districts that enabled individuals below the poverty threshold to purchase vouchers for long-term or permanent contraceptive methods, which could be redeemed at a variety of public, private for-profit and private not-for-profit providers[27]. The FP voucher programme received criticism concerning the limited uptake of the scheme[2,28] and lack of demand generation activities. Some also suggested that the FP voucher fee of KES 100 (approximately US $1.25) was still

For peer review only - http://bmjopen.bmj.com/site/about/guidelines.xhtml

relatively costly for the poorest users[3], though this is half the reported median cost (KES 200) for implant in the public sector in our study. Studies in Kenya have found many women express a preference for or high satisfaction with FP services at private sector facilities[29–31]. Initiatives to expand the range of affordable providers offering high-quality care and a range of contraceptives are still an important component of ensuring FP access and choice.

Respondent's region was significantly associated with differences in reporting free FP and the amount paid for injectable and implant. In 2010, Kenya's Ministry of Health devolved decision-making power and budgets to the county level, though policy continued to be set at the national level[32]. Despite recent gains in national modern contraceptive prevalence and reduction of unmet need, large regional disparities in coverage remain[19]. Differences in regional levels of free FP, with a substantially higher proportion of users in Nairobi reporting free FP compared to nearly all other regions, suggest that counties may be operating different systems of payment for contraception or distribution channels for FP commodities. Public primary care facilities in Kenya have long faced challenges of resource scarcity[33]. As facilities cannot directly charge for FP under current policy, when faced with declining revenue, they may introduce indirect charges, framed as registration fees or other costs, to recoup expenses[11]. Efforts to reimburse primary care facilities to account for the abolishment of user fees have been at relatively low levels, and as our findings also show, user fees above those set in national policy continue to be charged[33]. Further research is needed to understand sub-national implementation of national FP policy, the impact of facility-level strategies to cope with financial shortfalls on user's access to care and the reasons users are charged for contraceptive services.

The considerable variation in free services by method in public facilities possibly reflects differing auxiliary costs associated with dispensing methods, with, for example, more staff time and medical equipment required to insert IUDs and implants compared to condoms, which are often available without a consultation. We found that injectable users were significantly less likely to report receiving this method for free compared to long-acting IUD and implants or even the pill, raising questions about the long-term cost burden to users, who require re-supply every three months for continued coverage, for this popular method.

Limitations

This study was limited in relying on the accuracy of women's self-report of their method, source and cost of FP. While current injectable users needed to recall how much was paid up to three months earlier, some current implant users were asked to report the amount paid up to three years prior to interview, though median length of implant use was less than 17 months. Additionally, we were only able to consider cost and source among women who were current users of FP. Findings are likely not generalisable to former implant or injectable users, particularly if they discontinued due to costs associated with obtaining their method of choice, or to prospective users who were discouraged from initiating FP due to costs associated.

The DHS question regarding contraceptive cost asked for the total paid for commodity and consultation, and it did not capture costs associated with time and travel to obtain the method. These may be significant, particularly for rural users. We were unable to estimate the share of OOP payment for FP from total income because the DHS does not collect information on household/individual income or expenditures. As such, we cannot draw conclusions about the extent to which the amount paid for FP represents an undue burden on individual users. Additionally, DHS household wealth quintiles may not align with the poverty definition used to determine FP fee waivers or offer sufficient nuance to distinguish very disadvantaged households[34].

For peer review only - http://bmjopen.bmj.com/site/about/guidelines.xhtml

We acknowledge that the first consultation visit to initiate the contraceptive method may be longer, involving counselling and taking of medical history, than a re-supply visit and could result in increased cost. However, we compared results for initiators, users starting injectable and implant less than three months and three years, respectively, before the survey where the cost paid likely refers to the initiating consultation, against re-supply users, those starting the method more than three months or years prior to interview. Yet we found initiating users reported slightly lower mean costs than re-suppliers, though differences were not significant (results not shown).

Finally, FP budget implementation is done at the county-level in Kenya, yet the DHS FP cost question was intended to be representative at national, urban/rural and regional levels only[19] and thus county-level results could not be examined.

Conclusions

Removing or subsidising costs for the poor is a core component of an equitable system of user fees for healthcare, yet our findings highlight that the poorest contraceptive users in the public sector were as likely to pay for FP services as wealthier users. Kenya's National Reproductive Health Strategy (2009-2015) outlined pro-poor strategies and objectives to increase equity of FP access. The Kenyan government has made important progress in expanding FP access but more attention is needed to implementation of user fee policies, particularly to ensure the poorest receive affordable services and to account for geographic variation, ensuring recent efforts to reimburse facilities for lost user fee revenue are done at appropriate levels. However, public sector resources alone are unlikely to meet Kenya's growing demand for modern contraception. Policymakers should consider how government resources could be targeted at those least able to tap the private sector for FP care. While individual price discrimination offers one route to targeting public services to the poor, efforts could also focus resources—including outreach campaigns about patients' rights and correct fees—toward facilities in poor areas or toward increasing choice of affordable methods and accessible, high-quality providers for the poor. Fulfilling the promise of equity in FP access in Kenya demands turning policy intention into sustainable action from the national to facility-level.

List of abbreviations

DHS: Demographic & Health Surveys **FP**: Family planning **IUD:** Intrauterine device
KES: Kenyan Shillings **LMICs**: Low- and middle-income countries
NGO: Non-governmental organisation **OOP:** Out-of-pocket **USD**: United States Dollar

DECLARATIONS

Ethics approval

The DHS receive government permission and follow ethical practices including informed consent and assurance of confidentiality. The Research Ethics Committee of the London School of Hygiene and Tropical Medicine approved our secondary-data analysis.

Data sharing

The data that support the findings of this study are owned by the Demographic and Health Surveys (DHS) Program, operated by ICF International. Restrictions apply to the availability of these data, which were used under license for the current study, and so are not publicly available. Data are available for free from the DHS Program website and available for researchers who apply for and meet the criteria for access. Legal access agreements do not allow the sharing of datasets to unregistered researchers.

Competing interests

The authors declare that they have no competing interests.

For peer review only - http://bmjopen.bmj.com/site/about/guidelines.xhtml

Funding statement

The research in this publication was supported by funding from MSD, through its MSD for Mothers program. MSD had no role in the design, collection, analysis and interpretation of data, in writing of the manuscript, or in the decision to submit the manuscript for publication. The content of this publication is solely the responsibility of the authors and does not represent the official views of MSD. MSD for Mothers is an initiative of Merck & Co., Inc., Kenilworth, N.J., U.S.A. Edwine Barasa is supported by a Wellcome Trust research training grant (#107527).

Authors' contributions

ER designed the research question, analysed data, and prepared the manuscript. LB, MD, CL and JB contributed to the design of the study. KW contributed to analysis of the data. LB, MD, FC and EB assessed interpretation of findings and contributed to manuscript revisions. JB, TA, KW and MLA reviewed and edited the manuscript. All authors read and approved the final manuscript.

REFERENCES

1 Munge K, Briggs AH. The progressivity of health-care financing in Kenya. *Health Policy Plan* 2014;**29**:912–20. doi:10.1093/heapol/czt073

2 Obare F, Warren C, Kanya L, *et al.* Community-level effect of the reproductive health vouchers program on out-of-pocket spending on family planning and safe motherhood services in Kenya. *BMC Health Serv Res* 2015;**15**:343. doi:10.1186/s12913-015-1000-3

3 Health Policy Initiative. The EQUITY Framework: Influencing Policy and Financing Reforms to Increase Family Planning Access for the Poor in Kenya. Washington, DC: : Futures Group 2010.

4 Haghparast-Bidgoli H, Pulkki-Brännström A-M, Lafort Y, *et al.* Inequity in costs of seeking sexual and reproductive health services in India and Kenya. *Int J Equity Health* 2015;**14**:84. doi:10.1186/s12939-015-0216-5

5 Holtz J. Achieving Universal Access to Family Planning Services. Bethesda, MD: : Sustaining Health Outcomes through the Private Sector (SHOPS) Plus Project, Abt Associates 2016.

6 Chuma J, Gilson L, Molyneux C. Treatment-seeking behaviour, cost burdens and coping strategies among rural and urban households in Coastal Kenya: an equity analysis. *Trop Med Int Heal* 2007;**12**:673–86. doi:10.1111/j.1365-3156.2007.01825.x

7 Korachais C, Macouillard E, Meessen B. How User Fees Influence Contraception in Low and Middle Income Countries: A Systematic Review. *Stud Fam Plann* 2016;**47**:341–56. doi:10.1111/sifp.12005

8 Sedgh G, Ashford LS, Hussain R. Unmet Need for Contraception in Developing Countries: Examining Women's Reasons for Not Using a Method. New York: : Guttmacher Institute 2016.

9 Sedgh G, Hussain R. Reasons for Contraceptive Nonuse among Women Having Unmet Need for Contraception in Developing Countries. *Stud Fam Plann* 2014;**45**:151–69. doi:10.1111/j.1728-4465.2014.00382.x

10 Janisch CP, Albrecht M, Wolfschuetz A, *et al.* Vouchers for health: A demand side output-based aid approach to reproductive health services in Kenya. *Glob Public Health* 2010;**5**:578–94. doi:10.1080/17441690903436573

11 Chuma J, Musimbi J, Okungu V, *et al.* Reducing user fees for primary health care in Kenya: Policy on paper or policy in practice? *Int J Equity Health* 2009;**8**:15. doi:10.1186/1475-9276-8-15

12 Chuma J, Maina T. Free Maternal Care and Removal of User Fees at Primary-Level Facilities in

For peer review only - http://bmjopen.bmj.com/site/about/guidelines.xhtml

Kenya: Monitoring the Implementation and Impact—Baseline Report. Washington, DC: : Futures Group, Health Policy Initiative 2013.

13 National Coordinating Agency for Population and Development, Ministry of Medical Services, Ministry of Public Health and Sanitation, *et al.* Kenya Service Provision Assessment Survey 2010. Nairobi, Kenya: 2011.

14 National Reproductive Health Strategy, 2009-2015. Nairobi, Kenya: : Ministry of Public Health and Sanitation & Ministry of Medical Services (Republic of Kenya)

15 Kenya Reproductive, Maternal, Newborn, Child and Adolescent Health (RMNCAH) Investment Framework. Nairobi, Kenya: : Ministry of Health (Republic of Kenya) 2016.

16 Health Policy Initiative. FP/RH Access for the Poor in Kenya: EQUITY Framework Encourages Policy and Financing Reforms. Washington, DC: : Futures Group 2010.

17 POLICY Project. Targeting: A Key Element of National Contraceptive Security Planning. Washington, DC: : Futures Group 2003.

18 Health Policy Initiative. The cost of family planning in Kenya. Washington, DC: : Futures Group 2010.

19 Kenya National Bureau of Statistics, Ministry of Health, National AIDS Control Council, *et al.* Kenya Demographic and Health Survey 2014. Nairobi, Kenya and Rockville, MD: : ICF International 2015.

20 Hubacher D, Trussell J. A definition of modern contraceptive methods. *Contraception* 2015;**92**:420–1. doi:10.1016/j.contraception.2015.08.008

21 Rutstein SO, Johnson K. The DHS Wealth Index (DHS Comparative Reports #6). Calverton, MD: : ORC Macro 2004.

22 Filzmoser P, Gussenbauer J, Templ M. Detecting outliers in household consumption survey data. Vienna, Austria: : Vienna University of Technology 2016.

23 XE Currency Charts: USD to KES. http://www.xe.com/currencycharts/?from=USD&to=KES&view=5Y (accessed 5 Dec 2016).

24 Skordis-Worrall J, Pace N, Bapat U, *et al.* Maternal and neonatal health expenditure in Mumbai slums (India): a cross sectional study. *BMC Public Health* 2011;**11**:150. doi:10.1186/1471-2458-11-150

25 Stata. testnl — Test nonlinear hypotheses after estimation. https://www.stata.com/manuals13/rtestnl.pdf (accessed 11 Jan 2018).

26 Chakraborty NM, Wanderi J, Oduor C, *et al.* Assessing provision and equity in low and middle income country health markets: a study from Kenya. Oakland, CA: : Metrics for Management 2017.

27 Arur A, Gitonga N, O'Hanlon B, *et al.* Insights from innovations: Lessons from designing and implementing family planning / reproductive health voucher programs in Kenya and Uganda. Bethesda, MD: : Private Sector Partnerships-One project, Abt Associates Inc. 2009.

28 Njuki R, Obare F, Warren C, *et al.* Community experiences and perceptions of reproductive health vouchers in Kenya. *BMC Public Health* 2013;**13**:660. doi:10.1186/1471-2458-13-660

29 Keesara SR, Juma PA, Harper CC, *et al.* Why do women choose private over public facilities for family planning services? A qualitative study of post-partum women in an informal urban settlement in Kenya. *BMC Health Serv Res* 2015;**15**:335. doi:http://dx.doi.org/10.1186/s12913-015-0997-7

For peer review only - http://bmjopen.bmj.com/site/about/guidelines.xhtml

30 Agha S, Do M. The quality of family planning services and client satisfaction in the public and private sectors in Kenya. *Int J Qual Heal Care* 2009;**21**:87–96. doi:10.1093/intqhc/mzp002

31 Hutchinson PL, Do M, Agha S. Measuring client satisfaction and the quality of family planning services: a comparative analysis of public and private health facilities in Tanzania, Kenya and Ghana. *BMC Health Serv Res* 2011;**11**:203. doi:10.1186/1472-6963-11-203

32 Barker C, Mulaki A, Mwai D, *et al.* Assessing county health system readiness in Kenya: A review of selected health inputs. Washington, DC: : Futures Group 2014.

33 Nyikuri M, Tsofa B, Barasa E, *et al.* Crises and Resilience at the Frontline—Public Health Facility Managers under Devolution in a Sub-County on the Kenyan Coast. *PLoS One* 2015;**10**:e0144768. doi:10.1371/journal.pone.0144768

34 Wong KLM, Restrepo-Méndez MC, Barros AJD, *et al.* Socioeconomic inequalities in skilled birth attendance and child stunting in selected low and middle income countries: Wealth quintiles or deciles? *PLoS One* 2017;**12**:e0174823. doi:10.1371/journal.pone.0174823

For peer review only

For peer review only - http://bmjopen.bmj.com/site/about/guidelines.xhtml

FIGURE 1: METHOD MIX AND PROVIDER USE BY WEALTH QUINTILE AMONG CURRENT MODERN FP USERS

For peer review only

For peer review only - http://bmjopen.bmj.com/site/about/guidelines.xhtml

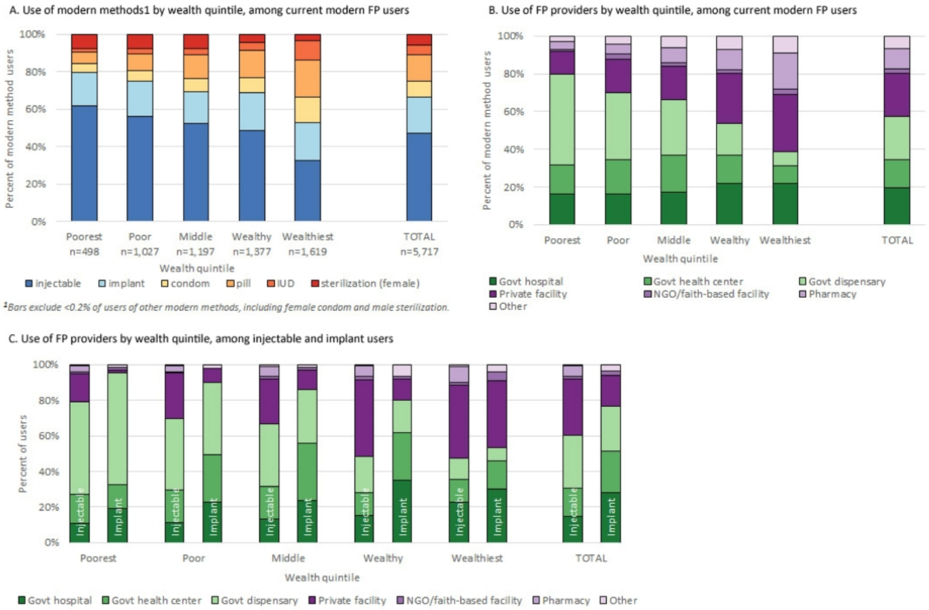

Method mix and provider use by wealth quintile among modern FP users

250x156mm (300 x 300 DPI)

Supplementary Table 1
Summary of out-of-pocket payment (in USD) for injectable and implant users among users with non-zero expenditure, by most recent provider of the contraceptive method, with different methods for dealing with outliers.

a. Observations greater than two standard deviations from the mean recoded as equal to the mean.

	Govt hospital	Govt health centre	Govt dispensary	TOTAL PUBLIC	Private facility	NGO/faith-based facility	Pharmacy/chemist	Other[1]	TOTAL
Injectable									
n	247	225	490	962	821	28	148	17	1,976
Mean cost in USD	$0.83	$0.78	$0.74	$0.75	$1.07	$1.01	$1.08	$1.06	$0.91
SD	0.412	0.399	0.385	0.343	0.277	0.560	0.278	0.280	0.349
25th percentile	$0.57	$0.57	$0.57	$0.57	$0.91	$0.57	$0.91	$1.14	$0.57
50th percentile (median)	$0.57	$0.57	$0.57	$0.57	$1.14	$0.80	$1.14	$1.14	$1.14
75th percentile	$1.14	$1.14	$1.14	$1.14	$1.14	$1.14	$1.14	$1.14	$1.14
Implant									
n	136	94	102	332	130	11	-	3	477
Mean cost in USD	$3.48	$2.90	$2.37	$2.97	$7.47	$6.43		$6.20	$4.31
SD	3.363	2.525	1.629	2.724	5.034	4.430		6.090	4.096
25th percentile	$2.28	$1.14	$1.14	$1.14	$3.42	$2.28		$1.14	$2.28
50th percentile (median)	$2.28	$2.28	$2.28	$2.28	$5.74	$9.12		$1.14	$2.28
75th percentile	$3.42	$3.42	$2.28	$3.42	$11.40	$9.12		$1.14	$5.70

b. Observations greater than two standard deviations from the mean recoded to missing.

	Govt hospital	Govt health centre	Govt dispensary	TOTAL PUBLIC	Private facility	NGO/faith-based facility	Pharmacy/chemist	Other[1]	TOTAL
Injectable									
n	237	216	476	929	794	21	144	17	1,905
Mean cost in USD	$0.81	$0.74	$0.71	$0.74	$1.07	$0.82	$1.09	$1.06	$0.91
SD	0.387	0.327	0.330	0.346	0.282	0.338	0.282	0.280	0.355
25th percentile	$0.57	$0.57	$0.57	$0.57	$0.91	$0.57	$0.91	$1.14	$0.57
50th percentile (median)	$0.57	$0.57	$0.57	$0.57	$1.14	$0.80	$1.14	$1.14	$1.14
75th percentile	$1.14	$1.14	$1.14	$1.14	$1.14	$1.14	$1.14	$1.14	$1.14
Implant									
n	135	94	102	331	109	11	-	3	454
Mean cost in USD	$3.47	$2.90	$2.37	$2.97	$7.81	$6.43	-	$6.20	$4.24
SD	3.367	2.525	1.629	2.724	5.454	4.430	-	6.090	4.183
25th percentile	$2.28	$1.14	$1.14	$1.14	$2.85	$2.28	-	$1.14	$2.28
50th percentile (median)	$2.28	$2.28	$2.28	$2.28	$5.70	$9.12	-	$1.14	$2.28
75th percentile	$3.42	$3.42	$2.28	$3.42	$11.40	$9.12	-	$11.40	$5.70

c. Observations greater than two standard deviations from the mean recoded as equal to the value two standard deviations from the mea

	Govt hospital	Govt health centre	Govt dispensary	TOTAL PUBLIC	Private facility	NGO/faith-based facility	Pharmacy/chemist	Other[1]	TOTAL
Injectable									
n	247	225	490	962	821	28	148	17	1,976
Mean cost in USD	$0.86	$0.80	$0.75	$0.79	$1.11	$1.13	$1.11	$1.06	$0.95
SD	0.454	0.419	0.396	0.419	0.331	0.628	0.319	0.280	0.412
25th percentile	$0.57	$0.57	$0.57	$0.57	$0.91	$0.57	$0.91	$1.14	$0.57
50th percentile (median)	$0.57	$0.57	$0.57	$0.57	$1.14	$0.80	$1.14	$1.14	$1.14
75th percentile	$1.14	$1.14	$1.14	$1.14	$1.14	$1.71	$1.14	$1.14	$1.14
Implant									
n	136	94	102	332	130	11	-	3	477
Mean cost in USD	$3.54	$2.90	$2.37	$3.00	$10.16	$6.43	-	$6.20	$5.06
SD	3.559	2.525	1.629	2.829	7.236	4.430	-	6.090	5.528
25th percentile	$2.28	$1.14	$1.14	$1.14	$3.42	$2.28	-	$1.14	$2.28
50th percentile (median)	$2.28	$2.28	$2.28	$2.28	$6.84	$9.12	-	$1.14	$2.28
75th percentile	$3.42	$3.42	$2.28	$3.42	$17.10	$9.12	-	$11.40	$5.70

For peer review only

d. Observations greater than 1.5 times the interquartile range recoded to missing.

	Govt hospital	Govt health centre	Govt dispensary	TOTAL PUBLIC	Private facility	NGO/faith-based facility	Pharmacy/ chemist	Other[1]	TOTAL
Injectable									
n	237	216	476	929	794	21	144	17	1,905
Mean cost in USD	$0.81	$0.74	$0.71	$0.74	$1.07	$0.82	$1.09	$1.06	$0.91
SD	0.387	0.327	0.330	0.346	0.282	0.338	0.282	0.280	0.355
25th percentile	$0.57	0.57	0.57	$0.57	0.912	0.57	0.912	1.14	$0.57
50th percentile (median)	$0.57	0.57	0.57	$0.57	1.14	0.798	1.14	1.14	$1.14
75th percentile	$1.14	1.14	1.14	$1.14	1.14	1.14	1.14	1.14	$1.14
Implant									
n	128	89	102	319	72	9	-	2	401
Mean cost in USD	$2.80	$2.43	$2.37	$2.56	$4.33	$5.12	-	$1.14	$2.93
SD	1.738	1.565	1.629	1.663	2.060	3.647	-	0.000	1.948
25th percentile	$2.28	$1.14	$1.14	$1.14	$2.28	$2.28	-	$1.14	$1.71
50th percentile (median)	$2.28	$2.28	$2.28	$2.28	$3.42	$2.28	-	$1.14	$2.28
75th percentile	$3.42	$2.85	$2.28	$3.42	$5.70	$9.12	-	$1.14	$3.42

e. Observations greater than 1.5 times the interquartile range recoded as equal to the value 1.5 times the interquartile range.

	Govt hospital	Govt health centre	Govt dispensary	TOTAL PUBLIC	Private facility	NGO/faith-based facility	Pharmacy/ chemist	Other[1]	TOTAL
Injectable									
n	247	225	490	962	821	28	148	17	1,976
Mean cost in USD	$0.86	$0.80	$0.75	$0.79	$1.10	$1.11	$1.11	$1.06	$0.95
SD	0.445	0.408	0.388	0.410	0.323	0.595	0.313	0.280	0.403
25th percentile	$0.57	$0.57	$0.57	$0.57	$0.91	$0.57	$0.91	$1.14	$0.57
50th percentile (median)	$0.57	$0.57	$0.57	$0.57	$1.14	$0.80	$1.14	$1.14	$1.14
75th percentile	$1.14	$1.14	$1.14	$1.14	$1.14	$1.71	$1.14	$1.14	$1.14
Implant									
n	136	94	102	332	130	11	-	3	477
Mean cost in USD	$3.25	$2.87	$2.37	$2.87	$7.27	$6.14	-	$5.92	$4.18
SD	2.515	2.424	1.629	2.274	3.583	3.994	-	5.751	3.394
25th percentile	$2.28	$1.14	$1.14	$1.14	$3.42	$2.28	-	$1.14	$2.28
50th percentile (median)	$2.28	$2.28	$2.28	$2.28	$6.84	$9.12	-	$1.14	$2.28
75th percentile	$3.42	$3.42	$2.28	$3.42	$10.83	$9.12	-	$10.83	$5.70

[1] Includes DHS response options: mobile clinic and other private medical

SD: Standard deviation

For peer review only

Supplementary Table 2

Out-of-pocket payment (in USD) for injectable and implant across all provider types among users with non-zero expenditure by socio–demographic characteristics.

	Injectable				Implant			
	n	Median	Mean (95% CI)		n	Median	Mean (95% CI)	
Wealth quintile								
Poorest	209	$0.80	$0.84 (0.77-0.91)		29	$2.28	$3.35 (1.88-4.81)	
Poor	417	$0.80	$0.82 (0.78-0.86)		89	$2.28	$2.78 (2.43-3.13)	
Middle	459	$0.80	$0.89 (0.84-0.93)	p<0.001	81	$2.28	$3.03 (2.54-3.53)	p<0.001
Wealthy	519	$1.14	$0.97 (0.93-1.01)		101	$2.28	$4.06 (2.83-5.30)	
Wealthiest	379	$1.14	$1.14 (1.08-1.21)		177	$5.70	$5.95 (4.74-7.17)	
Residence								
Urban	792	$1.14	$1.07 (1.03-1.11)	p<0.001	230	$3.42	$5.18 (4.15-6.21)	p=0.005
Rural	1191	$0.80	$0.85 (0.82-0.88)		246	$2.28	$3.49 (2.94-4.04)	
Educational attainment								
Less than primary	615	$0.91	$0.87 (0.84-0.91)		115	$2.28	$3.88 (2.90-4.85)	
Less than secondary	915	$1.14	$0.94 (0.90-0.98)	p<0.001	202	$2.28	$3.36 (2.78-3.94)	p=0.004
Secondary+	453	$1.14	$1.03 (0.98-1.08)		160	$3.42	$5.81 (4.49-7.14)	
Age group								
<20 years	77	$1.14	$0.92 (0.85-1.00)		9	$4.56	$3.50 (1.95-5.04)	
20–29 years	1032	$1.14	$0.94 (0.90-0.98)	p=0.897	226	$2.28	$4.20 (3.47-4.93)	p=0.594
30+ years	874	$1.14	$0.94 (0.90-0.97)		242	$2.28	$4.44 (3.50-5.38)	
Region†								
Central	207	$1.14	$1.07 (1.01-1.14)		87	$3.42	$4.51 (3.47-5.56)	
Coast	125	$1.14	$0.99 (0.87-1.11)		11	$2.28	$4.32 (1.35-7.29)	
Eastern	425	$0.91	$0.91 (0.85-0.96)		67	$3.42	$4.72 (3.79-5.64)	
Nairobi	183	$1.14	$1.18 (1.05-1.30)	p<0.001	51	$5.74	$8.03 (4.27-11.78)	p<0.001
Nyanza	315	$0.57	$0.84 (0.79-0.90)		66	$2.28	$2.90 (2.11-3.70)	
Rift Valley	495	$1.14	$0.93 (0.89-0.98)		129	$2.28	$4.08 (3.13-5.02)	
Western	232	$0.80	$0.81 (0.75-0.87)		64	$2.28	$2.58 (2.08-3.07)	

†Due to the very low modern contraceptive prevalence (<5%), results for the North Eastern region are not presented.

For peer review only

Supplementary Table 3

Proportion reporting free family planning by provider among users initiating their long–acting contraceptive method before or after June 2013 abolition of fees.

	Govt hospital<br>% (95% CI)	Govt health centre<br>% (95% CI)	Govt dispensary<br>% (95% CI)	TOTAL PUBLIC<br>% (95% CI)
Initiated before June 2013				
IUD (n=898)	49.9 (36.6–63.2)	78.8 (59.5–90.4)	76.9 (56.4–89.6)	61.2 (51.5–70.1)
Implant (n=469)	51.3 (40.6–61.9)	62.7 (51.5–72.7)	57.8 (48.3–66.7)	56.6 (50.4–62.7)
Initiated from June 2013 onward				
IUD (n=48)	48.5 (26.4–71.2)	69.2 (41.6–87.6)	64.6 (32.1–87.6)	59.2 (42.8–73.7)
Implant (n=357)	61.2 (50.5–70.9)	63.2 (50.0–74.7)	65.6 (56.0–74.0)	63.4 (56.9–69.5)

For peer review only

BMJ Open

Who pays and how much? A cross-sectional study of out-of-pocket payment for modern contraception in Kenya

Journal:	*BMJ Open*
Manuscript ID	bmjopen-2018-022414.R2
Article Type:	Research
Date Submitted by the Author:	19-Oct-2018
Complete List of Authors:	Radovich, Emma; London School of Hygiene and Tropical Medicine, Faculty of Epidemiology & Population Health<br>Dennis, Mardieh; London School of Hygiene and Tropical Medicine, Faculty of Epidemiology & Population Health<br>Barasa, Edwine; KEMRI-Wellcome Trust Research Programme, Health Economics Research Unit<br>Cavallaro, Francesca; London School of Hygiene and Tropical Medicine, Faculty of Epidemiology & Population Health<br>Wong, Kerry; London School of Hygiene and Tropical Medicine, Faculty of Epidemiology & Population Health<br>Borghi, Josephine; London School of Hygiene and Tropical Medicine, Faculty of Public Health & Policy<br>Lynch, Caroline; London School of Hygiene and Tropical Medicine, Faculty of Epidemiology & Population Health<br>Lyons-Amos, Mark; London School of Hygiene and Tropical Medicine, Faculty of Epidemiology & Population Health<br>Abuya, Timothy; Population Council Kenya<br>Benova, Lenka; London School of Hygiene and Tropical Medicine, Faculty of Epidemiology & Population Health; Instituut voor Tropische Geneeskunde, Department of Public Health
**Primary Subject Heading**:	Global health
Secondary Subject Heading:	Health services research
Keywords:	Kenya, family planning, user fees, Demographic & Health Survey, private sector, government provision

SCHOLARONE™ Manuscripts

Who pays and how much? A cross-sectional study of out-of-pocket payment for modern contraception in Kenya

Authors: Emma Radovich[1], Mardieh L. Dennis[1], Edwine Barasa[3], Francesca Cavallaro[1], Kerry L.M. Wong[1], Josephine Borghi[2], Caroline A. Lynch[1], Mark Lyons-Amos[1], Timothy Abuya[4], and Lenka Benova[1,5]

[1] Faculty of Epidemiology and Population Health, London School of Hygiene & Tropical Medicine, London, UK
[2] Faculty of Public Health and Policy, London School of Hygiene & Tropical Medicine, London, UK
[3] Health Economics Research Unit, KEMRI-Wellcome Trust Research Programme, Nairobi, Kenya
[4] Population Council, Nairobi, Kenya
[5] Department of Public Health, Institute of Tropical Medicine, Antwerp, Belgium

Corresponding author:
Emma Radovich, London School of Hygiene & Tropical Medicine
Keppel Street
London WC1E 7HT
United Kingdom
+44(0)20 7612 7922
emma.radovich@lshtm.ac.uk

Word count: 4,304

Keywords: Kenya; family planning; user fees; Demographic & Health Survey; private sector; government provision

For peer review only

ABSTRACT (300 words)

Objectives: Out-of-pocket (OOP) payment for modern contraception is an under-studied component of healthcare financing in countries like Kenya, where wealth gradients in met need have prompted efforts to expand access to free contraception. This study aims to examine whether, among public sector providers, the poor are more likely to receive free contraception and to compare how OOP payment for injectables and implants—two popular methods—differs by public/private provider type and user's socio-demographic characteristics.

Design, setting and participants: Secondary analyses of nationally representative, cross-sectional household data from the 2014 Kenya Demographic and Health Survey. Respondents were women of reproductive age (15-49 years). The sample comprised 5,717 current modern contraception users, including 2,691 injectable and 1,073 implant users with non-missing expenditure values.

Main outcome: Respondent's self-reported source and payment to obtain their current modern contraceptive method.

Methods: We used multivariable logistic regression to examine predictors of free public sector contraception and compared average expenditure for injectable and implant. Quintile ratios examined progressivity of non-zero expenditure by wealth.

Results: Half of public sector users reported free contraception; this varied considerably by method and region. Users of implants, condoms, pills and IUDs were all more likely to report receiving their method for free ($p<0.001$) compared to injectable users. The poorest were as likely to pay for contraception as the wealthiest users at public providers (OR: 1.10, 95%CI: 0.64-1.91). Across all providers, among users with non-zero expenditure, injectable and implant users reported a mean OOP payment of KES 80 (US $0.91), 95%CI: KES 78-82 and KES 378 (US $4.31), 95%CI: KES 327-429, respectively. In the public sector, expenditure was pro-poor for injectable users yet weakly pro-rich for implant users.

Conclusions: More attention is needed to targeting subsidies to the poorest and ensuring government facilities are equipped to cope with lost user fee revenue.

ARTICLE SUMMARY

Strengths and limitations of this study

- A major strength of the study is that it is the first to our knowledge to use nationally representative data from a low-income country to examine out-of-pocket payment for modern contraception.
- Another strength is the transparency in the classification of family planning providers, handling of outliers and appropriate adjustments for complex survey design.
- One limitation of the study is the reliance on self-reported cost data from current users of modern contraception and the inability to compare this with costs to women who discontinued or eschewed use of modern contraception.

For peer review only - http://bmjopen.bmj.com/site/about/guidelines.xhtml

For peer review only

BACKGROUND

Achieving Universal Health Coverage—including for family planning (FP) services—demands attention to financial protection. Consideration of user fees is particularly important in countries like Kenya, where out-of-pocket (OOP) payments form a substantial proportion of healthcare financing[1,2]. In Kenya, unmet need for FP is highest among the poor, with a documented 8-14 percentage point increase in modern contraception use with each increase in household wealth quintile[3]. A study in Kenya and India found that poor households spend a significantly higher proportion of their income on reproductive health care (including FP), with the poorest households in Kenya spending 10 times the proportion spent by the least poor[4]. Many government financial protection policies focus on inpatient events where healthcare expenditure is likely to be catastrophic, yet the greater frequency of outpatient expenses—including for contraceptive services, which affect women in particular—can also push households into poverty[5] or reduce care-seeking among the poor[3,6].

A systematic review on the relationship between user fees and FP use in low- and middle-income countries (LMICs) was inconclusive, though some included studies suggested that young people and the poor were more sensitive to price increases than wealthier or less marginalised groups[7]. Cost is rarely cited as the reason for non-use of modern contraception among women in need (those wishing to delay or avoid pregnancy) in Demographic and Health Surveys (DHS) [8,9]. Yet, focus groups in Nyanza Province, Kenya found that the poor identify high cost of services as a barrier to FP care, both in opportunity costs associated with seeking care and direct fees for services[3], suggesting that for some individuals, cost can impact FP access.

Kenya has used various financing mechanisms to support increased access to FP and reproductive health services[3,10]. A 2004 policy, commonly known as the "10/20 policy", abolished user fees in primary care facilities; instead government dispensaries and health centres were allowed to charge a registration fee of 10 or 20 Kenyan shillings (KES) (approximately US $0.11 and $0.23), with the poor exempted from payment[11,12]. Public hospitals could continue charging fees to users under a cost sharing policy. Yet fee waiver implementation and identification of eligible individuals was left to the discretion of actors at the community and facility-level. Despite the 10/20 policy, many FP clients in government facilities reported paying additional 'hidden fees' for the consultation, medical tests or equipment, and the contraceptive commodity[3]. A 2010 health facility survey found that approximately 70% of government facilities providing FP charged user fees for services[13]. A 2009 study found low community knowledge of the 10/20 policy and qualifying exemptions[11]. However, as of June 2013, all fees at public outpatient primary care facilities (dispensaries and health centres) were eliminated[12], and FP services are intended to be provided for free at public facilities[14,15]. The extent to which users currently receive free FP services from public outpatient primary care facilities is unknown, and similar to the 10/20 policy, implementation of the June 2013 policy for free FP services may vary, for example by facility type, geographic region or client characteristics.

Efforts to achieve universal coverage for reproductive health have led to increasing calls by donors and others for a "total market approach" in considering the different contributions of public and private providers. In this approach, government- or otherwise-subsidised services are targeted to meet the needs of the poor while individuals with the ability to pay are indirectly encouraged to seek FP services from commercial or unsubsidised private providers[3,5,16,17]. Kenya's changing fee policies within the public sector and the country's growing private sector, which now owns half of all health facilities[12], raise questions about where individuals, especially the poor, seek FP and what this means for their OOP payment for modern contraception. Little is known about OOP payment to obtain modern contraception in sub-Saharan Africa, and in Kenya in particular, and how this varies by provider type. In the context of limited resources to expand FP access[18], it is important

For peer review only - http://bmjopen.bmj.com/site/about/guidelines.xhtml

to understand the burden of user fees—who pays and how much—and the degree to which vulnerable groups are served by current efforts to provide affordable care.

This paper aims to address these knowledge gaps by describing FP sources by user's wealth in Kenya, examining, among public sector users, who receives free FP services, and comparing how payment for injectables and implants—the two most commonly used methods—differs by FP provider type and the user's socio-demographic characteristics.

METHODS

Data source

We used data from the most recent Kenya DHS (2014), a nationally representative, cross-sectional household survey of women age 15-49 with a multi-level cluster sampling design. A detailed description of the survey sampling can be found in the Kenya 2014 DHS report[19]. Interviews were administered between May and October 2014. Our analysis includes women in half of the randomly selected households who were administered the long-version Woman's Questionnaire (unweighted n=14,741), which included a question on the amount paid for the respondent's current contraceptive method [19]. Respondents were not asked for the reason for the payment.

Study populations

We examined data from three populations of women: 1) current users of modern contraception, based on the Hubacher & Trussell definition of modern methods[20]; 2) users of IUD, implant, injectable, pill and male condom as these users were asked to self-report the total amount paid to obtain their method (the combined cost of the commodity and any consultation fees) during their most recent (re-)supply visit; and 3) users of injectable and implant, where estimates of OOP payment refer to a single quantity of the contraceptive, as users can receive only one "dose" during insertion or re-supply. Respondents with missing or "don't know" expenditure values accounted for 4.4% of all users in group two, and less than 1% of injectable and implant users, and were excluded from analysis.

Definitions

We classified women's self-reported most recent source of modern FP into seven provider categories: 1) government hospital; 2) government health centre; 3) government dispensary; 4) private facility, a constructed category comprising DHS response options of private hospital/clinic and private nursing/maternity home; 5) NGO/faith-based facility; 6) pharmacy/chemist; and 7) other, a constructed category of the response options: shop, mobile clinic, friend/relative, other, community health worker, community-based distributor and other private medical. We defined the public sector to be government-provided services (categories 1-3) and non-public providers to be categories 4-7. We consider public primary care providers to be categories 2 and 3. Less than 1% of all current modern contraceptive users were missing the source of their method and were excluded from analysis.

We examined three measures of the respondents' socio-economic status: household wealth quintiles derived by the DHS from household assets[21], urban/rural residence, and three levels of educational attainment: less than primary school (respondents with no education and those who started but did not complete primary school), less than secondary school (respondents with complete primary or incomplete secondary school) and secondary+ (respondents with complete secondary or some higher education). We used DHS categories for respondent's current marital status (never, currently or formerly in union) and grouped respondents by their current age: less than 20, 20-29 and 30+ years. Kenya is administratively divided into 47 counties; however the

For peer review only - http://bmjopen.bmj.com/site/about/guidelines.xhtml

variable for OOP payment for contraception in the 2014 Kenya DHS was intended to provide representative estimates for the national level, for urban and rural areas, and for the eight regions (former provinces)[19].

Analysis of free or 'registration fee only' FP in the public sector

We limited analysis of free FP to users whose most recent source of the method was a public sector provider. We include both categories of public primary care providers (subject to the June 2013 abolishment of fees) and government hospitals as a point of comparison. Adjusted Wald tests were performed to compare proportions reporting free FP by facility type and user characteristics. Bivariable and multivariable logistic regression was used to examine predictors, such as wealth quintile, facility type and region, of receiving free FP from public primary care facilities, as indicated under the 2013 policy.

Users of long-acting methods like IUD and implant could report OOP payment based on FP consultations before the June 2013 abolishment of fees. Thus, estimates of free FP for IUD and implant were further disaggregated by whether the method was obtained before or after June 2013 based on the respondent's self-reported month and year of initiating use of the method.

Facility-level implementation of the June 2013 policy abolishing all fees at public primary care facilities may not have been immediate. As such, we additionally examined the proportion of users who reported paying up to 10 KES or 20 KES at a government dispensary or health centre, respectively, referring to these users as paying 'registration fees only' consistent with the former 10/20 policy, though respondents did not indicate the reason for the charge.

Analysis of OOP payment for injectable and implant

Prior to analysis, we assessed the data for improbable values and recoded observations to missing if reported expenditure was greater than 10 times the 95th percentile (six observations). Among injectable and implant users reporting non-zero cost, we described the patterns of OOP expenditure, reporting mean and median values. We conducted sensitivity analyses to ensure the robustness of our results, comparing results from multiple methods for dealing with outliers[22]; results did not differ substantially (Supplementary Table 1). For this analysis, observations greater than two standard deviations (SD) from the mean (2.7% and 2.1% of injectable and implant users, respectively) were recoded to be equal to the mean. Simple linear regression and marginal effects were used to compare means between providers and user characteristics. We additionally present estimates of OOP payment converted from KES to US dollars (USD) based on 1 KES to 0.0114 USD conversion rate for the midpoint of fieldwork in July 2014[23] in Supplementary Tables 1-2.

Equity of OOP payment for injectable and implant

Quintile ratios were used to measure the progressiveness of OOP payments for injectables and implants overall and within the public sector. This measure of equity in expenditure assumes that individuals in the lowest wealth quintile have less capacity to pay and thus if they spend the same or more as those in the highest quintile, this represents a greater proportion of income and constitutes regressive spending[24]. Quintile ratios were calculated by comparing mean expenditure in the wealthiest and poorest wealth quintiles and testing for differences in using an adjusted Wald-type test of nonlinear hypotheses based on the delta method, attributing significance at a 95% confidence level[4,24,25]. We define expenditure as weakly pro-rich if there was no significant difference in mean payment between the poorest and wealthiest users and strongly pro-rich if the poorest users paid significantly more than the wealthiest users (quintile ratio <1)[4,24].

All analysis used women's individual sampling weights and standard error adjustment to account for complex survey design. Analyses were conducted in Stata/SE v14 (StataCorp, College Station, TX, USA).

For peer review only - http://bmjopen.bmj.com/site/about/guidelines.xhtml

Patient and public involvement statement

Patients and the public were not involved in this secondary data analysis.

RESULTS

A total of 5,717 (weighted n) modern contraceptive users with non-missing provider data were included in our analysis sample.

Methods and sources of family planning

Among all current modern FP users, the wealthiest quintile had the broadest mix of methods, with no single method accounting for more than a third of modern FP users (Figure 1a). In contrast, method mix among users in the three poorer quintiles was dominated by injectables, which accounted for more than half of methods used. While injectables and implants were the two most popular methods for all users, this was particularly true for the poorest users, where these two methods accounted for nearly 80% of all modern methods used.

The wealthiest contraceptive users also reported a broader mix of providers (Figure 1b). Among the poorest users, 80.0% reported a public sector source. Public provider use decreased steadily and use of private facilities and pharmacies increased with increasing wealth quintile. The wealthiest users reported the largest use of private facilities (30.5%) and pharmacies (18.7%). Among injectable users, public sector providers were the most-used source for the three poorest quintiles, with a clear decline in government dispensary use with increasing wealth (Figure 1c). The vast majority of implants in the four poorer wealth quintiles were sourced from public providers, and there was a dramatic increase in use of private facilities for implants in the fifth, wealthiest quintile. (Supplementary Table 3 shows the distribution of all modern methods by provider type.)

Free family planning

Users of injectable, implant, pill, condom and IUD were asked to self-report the total amount paid to obtain their method. Overall, 51.1% of public sector users reported obtaining their modern FP method for free at their most recent visit (Table 1). This varied by method used: more than 90% of condom users compared to 40.7% of injectable users reported free FP. Across the three levels of facilities, 50.1% of government hospital, 56.2% of government health centre, and 48.5% of government dispensary users reported free FP, with some evidence of a difference by facility type (p=0.048). The percentage of women obtaining free FP in public facilities differed only slightly by respondent's wealth quintile, urban/rural residence, education level, or age group. The proportion of users reporting free FP varied considerably by region, with 39.4% of Rift Valley residents compared to 76.6% of Nairobi residents reporting free contraception. Additionally, 1.3% (95% CI: 0.9-2.1%) of users of government health centres and dispensaries reported paying a 'registration fee only' amount under the former 10/20 policy (results not shown). There was no difference by user's wealth quintile.

For peer review only - http://bmjopen.bmj.com/site/about/guidelines.xhtml

Table 1: Among public sector providers, proportion reporting free family planning by modern method users' socio–demographic characteristics

	Govt hospital (n=929)	Govt health centre (n=815)	Govt dispensary (n=1,267)	TOTAL PUBLIC (n=3,011)
Overall (95% CI)	50.1 (45.9–54.3)	56.2 (50.9–61.4)	48.5 (45.0–52.1)	51.1 (48.5–53.7)
Method				
Injectable	38.4 (32.4–44.8)	46.0 (39.3–52.9)	39.2 (34.8–43.7)	40.7 (37.5–44.1)
Implant	55.0 (47.1–62.6)	63.0 (53.9–71.1)	61.5 (54.6–67.9)	59.6 (54.8–64.2)
Pill	68.7 (53.8–80.6)	66.0 (50.6–78.6)	61.1 (50.0–71.3)	64.7 (57.1–71.6)
Condom	90.8 (71.0–97.5)	92.8 (77.4–98.0)	97.4 (83.7–99.6)	93.6 (85.0–97.3)
IUD	49.6 (38.6–60.7)	75.4 (57.8–87.3)	73.4 (57.1–85.1)	60.7 (52.8–68.1)
Wealth quintile				
Poorest	61.7 (48.7–73.1)	54.1 (41.3–66.5)	46.1 (39.2–53.1)	50.2 (44.4–55.9)
Poor	51.2 (41.0–61.4)	51.7 (42.2–61.1)	44.3 (38.4–50.4)	47.6 (43.0–52.3)
Middle	43.2 (35.5–51.2)	52.4 (42.6–62.0)	48.9 (41.3–56.5)	48.6 (43.6–53.7)
Wealthy	51.8 (43.6–60.0)	57.1 (47.3–66.3)	54.4 (45.9–62.7)	54.2 (48.6–59.7)
Wealthiest	49.6 (42.0–57.2)	66.9 (55.0–76.9)	53.9 (39.8–67.4)	54.9 (48.7–61.0)
Residence				
Urban	49.1 (43.6–54.7)	66.0 (56.8–74.2)	56.1 (47.6–64.3)	55.2 (50.6–59.7)
Rural	51.6 (45.1–58.0)	50.9 (44.7–57.0)	46.8 (42.9–50.7)	48.8 (45.8–51.9)
Educational attainment				
Less than primary	56.7 (48.3–64.7)	55.1 (47.0–62.9)	47.6 (42.5–52.6)	51.7 (47.8–55.6)
Less than secondary	46.0 (40.0–52.0)	56.6 (49.3–63.6)	48.9 (43.8–53.9)	49.9 (46.4–53.5)
Secondary+	51.3 (43.0–59.4)	57.1 (46.9–66.6)	49.8 (40.9–58.7)	52.5 (47.0–58.0)
Age group				
<20 years	41.3 (19.4–67.4)	61.3 (40.2–78.9)	60.4 (42.8–75.7)	55.4 (43.4–66.9)
20–29 years	45.8 (40.0–51.7)	55.3 (48.1–62.4)	42.9 (37.9–48.1)	47.0 (43.5–50.6)
30+ years	54.2 (48.0–60.3)	56.5 (49.8–63.0)	52.7 (47.8–57.5)	54.2 (50.7–57.7)
*Region**				
Central	53.1 (43.5–62.4)	64.0 (50.7–75.4)	60.2 (47.8–71.4)	58.6 (51.3–65.5)
Coast	70.3 (58.9–79.7)	81.2 (69.6–89.0)	62.4 (52.4–71.4)	69.1 (62.1–75.2)
Eastern	35.2 (26.3–45.2)	40.4 (29.5–52.3)	44.5 (36.9–52.4)	41.5 (36.0–47.2)
Nairobi	70.4 (54.1–82.7)	76.0 (55.3–89.0)	–†	76.6 (63.4–86.0)
Nyanza	59.4 (49.8–68.3)	55.6 (44.2–66.4)	37.0 (30.0–44.7)	49.0 (43.1–55.0)
Rift Valley	30.9 (24.2–38.5)	48.0 (37.3–58.9)	42.8 (36.7–49.1)	39.4 (35.1–43.8)
Western	60.1 (43.6–74.5)	46.6 (34.2–59.4)	50.0 (39.8–60.3)	50.6 (43.5–57.7)

*Due to the very low modern contraceptive prevalence (<5%), results for the North Eastern region are not presented.

†No respondents reported this provider.

Among non-public sector providers (results not shown), 10.9% of private facility users and less than 1% of pharmacy users reported free FP. Of the limited number of users of NGO/faith-based facilities (n=91), 30.9% reported obtaining their contraceptive method for free.

Supplementary Table 4 shows the proportion of IUD and implant users receiving free FP from public sector providers among users initiating the method before and after the June 2013 fee abolishment. Among implant

For peer review only - http://bmjopen.bmj.com/site/about/guidelines.xhtml

users, the proportion receiving free FP from government health centres was similar between the two initiation periods and increased in the later period among users of government hospitals and dispensaries, though confidence intervals overlap. Among IUD users, the proportion receiving free care was slightly lower across all three public provider categories in the later initiation period, but differences were not statistically significant.

Table 2 shows the results of bivariable and multivariable analysis of receiving free modern FP among users of public primary care facilities, which were subject to the June 2013 fee abolishment policy. There were no differences by wealth quintile in the odds of obtaining free contraception after adjusting for method, provider type and user characteristics. Users of implants, condoms, pills and IUDs were all more likely to report receiving their method for free (p<0.001) compared to injectable users, and this relationship remained after adjusting for provider and user characteristics. Users in all regions had lower odds of free contraception compared to Nairobi, except Coast region (where it was not significantly different).

Table 2: Unadjusted and adjusted odds ratios from logistic regression analysis of reporting free family planning services from government primary care providers among modern method users

Variables	*Modern method users utilising public primary care providers (n=2,079)*			
	Unadjusted		Adjusted	
	OR	(95% CI)	OR	(95% CI)
Wealth quintile				
Poorest	0.58*	(0.37-0.91)	1.10	(0.64-1.91)
Poor	0.55**	(0.36-0.85)	1.20	(0.71-2.03)
Middle	0.64*	(0.41-0.99)	1.25	(0.74-2.11)
Wealthy	0.79	(0.51-1.24)	1.16	(0.67-2.01)
Wealthiest	Ref		Ref	
Provider				
Govt. health centre	Ref		Ref	
Govt. dispensary	0.74*	(0.57-0.95)	0.95	(0.74-1.22)
Method				
injectable	Ref		Ref	
implant	2.32***	(1.78-3.02)	2.15***	(1.62-2.86)
condom	29.87***	(9.84-90.66)	35.29***	(11.42-109.05)
pill	2.39***	(1.63-3.52)	2.27***	1.56-3.28)
IUD	4.14***	(2.26-7.56)	3.90***	(2.06-7.36)
Residence				
Urban	Ref		Ref	
Rural	0.58***	(0.43-0.79)	0.83	(0.60-1.14)
Region				
Central	0.36*	(0.14-0.93)	0.31*	(0.10-0.93)
Coast	0.48	(0.18-1.24)	0.53	(0.19-1.53)
Eastern	0.17***	(0.07-0.42)	0.18**	(0.06-0.52)
Nairobi	Ref		Ref	
Nyanza	0.18***	(0.07-0.44)	0.17**	(0.06-0.50)
Rift Valley	0.17***	(0.07-0.43)	0.19**	(0.07-0.53)
Western	0.21**	(0.08-0.53)	0.21**	(0.07-0.62)
Age group				
<20 years	1.31	(0.75-2.28)	1.58	(0.85-2.92)
20–29 years	0.76*	(0.61-0.95)	0.85	(0.67-1.08)
30+ years	Ref		Ref	
Marital status				

For peer review only

For peer review only - http://bmjopen.bmj.com/site/about/guidelines.xhtml

Never in union	0.93	(0.62-1.40)	0.80	(0.47-1.36)
Currently in union	Ref		Ref	
Formerly in union	1.38†	(0.96-1.98)	1.27	(0.88-1.83)

Note: Asterisks and daggers indicate that differences between the category and the reference category are significant at *p<0.05, **p<0.01, ***p<0.001, or marginally significant at †p<0.1.

Out-of-pocket payment for injectables and implants

Among injectable and implant users reporting greater than zero OOP payment to obtain the method from their most recent provider (both sectors combined), the mean cost was KES 80 (US $0.91) (95% CI: KES 78-82) for injectable and KES 378 (US $4.31) (95% CI: KES 327-429) for implant (Table 3). 1.7% of injectable and 1.5% of implant users reported paying amounts consistent with registration fees only (<KES 20). OOP payment varied, particularly for implant, by source of the method, with some private facility users reporting very high costs. Injectable users of public sector providers reported a median cost of KES 50, whereas the median cost was twice that (KES 100) for those accessing private facilities or pharmacies. Among implant users, those accessing public sector sources reported a median cost of KES 200, compared to a median cost of KES 503 among those utilising private facilities.

When assessed by user characteristics, mean OOP payment for both injectables and implants varied significantly by user's wealth, residence, education level and region, but not by user's age (Table 4). Urban and Nairobi residents paid more for both methods; this was particularly notable for implant users in Nairobi, where mean cost was more than twice that of implant users in Western or Nyanza regions. Mean and median cost did not increase linearly with increasing wealth quintile. For injectable users, median cost in the poorest three quintiles was KES 70 compared to KES 100 in the two wealthiest quintiles. For implant users, median cost of KES 500 in the wealthiest quintile was more than twice the median cost of KES 200 in the four poorer quintiles. The overall quintile ratio for all providers comparing mean cost in the wealthiest quintile to the poorest quintile was 1.3 (p<0.001) for injectable and 1.8 (p=0.007) for implant, indicating strong evidence of pro-poor OOP payment for both methods. Among public sector users, the quintile ratio was 1.2 (p=0.033) for injectable indicating pro-poor expenditure, and 0.90 (p=0.660) for implant (Table 5), indicating weakly pro-rich expenditure (no difference in mean cost between the quintiles) for public sector implant users.

For peer review only

Table 3: Summary of out-of-pocket payment (in KES) for injectable and implant users among users with non-zero expenditure, by most recent provider of the contraceptive method

	Govt hospital	Govt health centre	Govt dispensary	TOTAL PUBLIC	Private facility	NGO/faith-based facility	Pharmacy/ chemist	Other[1]	TOTAL
Injectable									
n	247	225	490	962	821	28	148	17	1,976
Mean cost	KES 72	KES 66	KES 63	KES 66	KES 94	KES 75	KES 95	KES 93	KES 80
SD	33.38	28.37	28.78	30.10	24.33	26.37	24.42	24.56	30.63
25th percentile	KES 50	KES 50	KES 50	KES 50	KES 80	KES 50	KES 80	KES 100	KES 50
50th percentile (median)	KES 50	KES 50	KES 50	KES 50	KES 100	KES 70	KES 100	KES 100	KES 100
75th percentile	KES 100	KES 100	KES 100	KES 100	KES 100	KES 87	KES 100	KES 100	KES 100
Reporting registration fees only	7.5%	5.3%	0.8%	3.6%					1.7%
Implant									
n	136	94	102	332	130	11	-	3	477
Mean cost	KES 305	KES 255	KES 208	KES 261	KES 655	KES 564		KES 544	KES 378
SD	295.01	221.51	142.92	238.98	441.62	388.58		534.17	359.25
25th percentile	KES 200	KES 100	KES 100	KES 100	KES 300	KES 200		KES 100	KES 200
50th percentile (median)	KES 200	KES 200	KES 200	KES 200	KES 503	KES 800		KES 100	KES 200
75th percentile	KES 300	KES 300	KES 200	KES 300	KES 1,000	KES 800		KES 1,000	KES 500
Reporting registration fees only	1.7%	5.0%	0.0%	2.1%					1.5%

[1] Includes DHS response options: mobile clinic and other private medical

SD: Standard deviation 1 KES = 0.0114 USD

For peer review only

For peer review only - http://bmjopen.bmj.com/site/about/guidelines.xhtml

Table 4: Out-of-pocket payment (in KES) for injectable and implant across all provider types among users with non-zero expenditure by socio–demographic characteristics

	**Injectable**						**Implant**					
	n	Median	Mean	(95% CI)			n	Median	Mean	(95% CI)		
Wealth quintile						Quintile ratio†						Quintile ratio†
Poorest	209	KES 70	KES 71	(66-77)			29	KES 200	KES 294	(165-422)		
Poor	417	KES 70	KES 71	(67-74)			89	KES 200	KES 244	(212-274)		
Middle	459	KES 70	KES 76	(73-79)	p<0.001		81	KES 200	KES 266	(223-309)	p<0.001	
Wealthy	516	KES 100	KES 83	(80-87)			101	KES 200	KES 357	(248-465)		
Wealthiest	379	KES 100	KES 96	(91-101)		1.3 (p<0.001)	177	KES 500	KES 522	(415-629)		1.8 (p=0.007)
Residence												
Urban	790	KES 100	KES 91	(88-94)	p<0.001		230	KES 200	KES 455	(364-545)	p=0.005	
Rural	1191	KES 70	KES 73	(71-75)			246	KES 300	KES 306	(258-355)		
Educational attainment												
Less than primary	615	KES 80	KES 75	(72-78)			115	KES 200	KES 340	(255-425)		
Less than secondary	915	KES 100	KES 80	(77-84)	p<0.001		202	KES 200	KES 295	(244-346)	p=0.004	
Secondary+	451	KES 100	KES 87	(83-91)			160	KES 300	KES 510	(394-626)		
Age group												
<20 years	77	KES 100	KES 81	(74-87)			9	KES 400	KES 307	(171-442)		
20–29 years	1030	KES 87	KES 80	(77-83)	p=0.928		226	KES 200	KES 369	(304-433)	p=0.594	
30+ years	874	KES 100	KES 80	(77-82)			242	KES 200	KES 389	(307-472)		
*Region**												
Central	207	KES 100	KES 90	(86-95)			87	KES 300	KES 396	(304-488)		
Coast	125	KES 100	KES 82	(73-92)			11	KES 200	KES 379	(119-639)		
Eastern	425	KES 80	KES 77	(73-81)			67	KES 300	KES 414	(333-495)		
Nairobi	183	KES 100	KES 101	(91-111)	p<0.001		51	KES 503	KES 704	(374-1034)	p<0.001	
Nyanza	315	KES 50	KES 72	(68-77)			66	KES 200	KES 255	(185-324)		
Rift Valley	492	KES 87	KES 80	(76-84)			129	KES 200	KES 358	(275-440)		
Western	232	KES 70	KES 69	(64-74)			64	KES 200	KES 226	(183-270)		

*Due to the very low modern contraceptive prevalence (<5%), results for the North Eastern region are not presented.

†Ratio of mean OOP expenditure comparing the wealthiest users to the poorest users. Adjusted Wald-type test based on the delta method was used to test for significance.
1 KES = 0.0114 USD

For peer review only - http://bmjopen.bmj.com/site/about/guidelines.xhtml

Table 5: Out-of-pocket payment (in KES) for public sector injectable and implant among users with non-zero expenditure by wealth quintile

	Injectable				Implant			
	n	Mean	(95% CI)	Quintile ratio†	n	Mean	(95% CI)	Quintile ratio†
Wealth quintile								
Poorest	147	KES 65	(59-72)		27	KES 267	(146-389)	
Poor	247	KES 61	(57-65)		76	KES 231	(200-262)	
Middle	256	KES 66	(62-70)		68	KES 253	(205-301)	
Wealthy	197	KES 66	(61-72)		78	KES 317	(187-447)	
Wealthiest	116	KES 78	(69-87)	1.2 (p=0.033)	82	KES 240	(190-291)	0.90 (p=0.660)

†Ratio of mean OOP expenditure comparing the wealthiest users to the poorest users. Adjusted Wald-type test based on the delta method was used to test for significance.
1 KES = 0.0114 USD

DISCUSSION

This is the first study to our knowledge to use nationally representative household data from a LMIC to examine equity of OOP payment for FP, comparing differences in cost to users accessing public and non-public providers. The wealthiest FP users in Kenya utilised a greater mix of modern methods and providers compared to the poorest users, and use of non-public providers increased with increasing wealth. Despite Kenya's national policy to offer free FP services at public primary care facilities, we found only half of modern method users reported obtaining their method at no cost from government providers, with little variation by facility type. There were no differences by user's socio-economic position. Among injectable and implant users reporting OOP expenditure, there were considerable differences by source of the method. Consistent with a previous study of FP users in urban Kenya[26], we found private facility and pharmacy users, unsurprisingly, reported higher expenditures than users of public facilities. Unfortunately, due to very small sample sizes (<30 users), OOP payment by users of NGO/faith-based facilities remains unclear, though there is some indication that costs may be higher than among public sector providers. Greater use of higher cost, non-public providers by the wealthiest users contributed to overall pro-poor expenditure, with both injectable and implant users in the wealthiest quintile paying significantly more than their counterparts in the poorest quintile.

A 'total market approach' to FP includes efforts to target government subsidies to the poorest contraceptive users and indirectly nudge wealthier users to seek FP from non-public providers. Evidence from this study suggests that market forces appear to be working to encourage greater use of non-public providers by the wealthiest users, though nearly 40% of FP users in the wealthiest quintile still sourced their method from the public sector. However, while the poorest users obtained their methods overwhelmingly from public providers they were equally likely to pay for FP as users in the wealthiest quintile, suggesting the potential for better targeting of free services to ensure the national pro-poor strategy of removing user fees for FP in public primary care facilities is reaching recipients most in need.

The Kenyan government faces the challenge of both meeting targets to reduce unmet need for FP[14] and ensuring all women, including the poor, have choice in FP methods and providers. Recent attempts to expand access to long-term methods, like implants, in Kenya have focused on expanding the range of providers available to the poor through vouchers. In 2005, Kenya launched a pilot system in five districts that enabled individuals below the poverty threshold to purchase vouchers for long-term or permanent contraceptive methods, which could be redeemed at a variety of public, private for-profit and private not-for-profit providers[27]. The FP voucher programme received criticism concerning the limited

For peer review only - http://bmjopen.bmj.com/site/about/guidelines.xhtml

For peer review only

uptake of the scheme[2,28] and lack of demand generation activities. Some also suggested that the FP voucher fee of KES 100 (approximately US $1.25) was still relatively costly for the poorest users[3], though this is half the reported median cost (KES 200) for implant in the public sector in our study. Studies in Kenya have found many women express a preference for or high satisfaction with FP services at private sector facilities[29–31]. Initiatives to expand the range of affordable providers offering high-quality care and a range of contraceptives are still an important component of ensuring FP access and choice.

Respondent's region was significantly associated with differences in reporting free FP and the amount paid for injectable and implant. In 2010, Kenya's Ministry of Health devolved decision-making power and budgets to the county level, though policy continued to be set at the national level[32]. Despite recent gains in national modern contraceptive prevalence and reduction of unmet need, large regional disparities in coverage remain[19]. Differences in regional levels of free FP, with a substantially higher proportion of users in Nairobi reporting free FP compared to nearly all other regions, suggest that counties may be operating different systems of payment for contraception or distribution channels for FP commodities. Public primary care facilities in Kenya have long faced challenges of resource scarcity[33]. As public primary care facilities cannot directly charge for FP under the current policy, when faced with declining revenue, they may introduce indirect charges, framed as registration fees or other costs, to recoup expenses[11]. Efforts to reimburse primary care facilities to account for the abolishment of user fees have been at relatively low levels, and as our findings also show, user fees above those set in national policy continue to be charged[33]. Further research is needed to understand sub-national implementation of the national FP policy, the impact of facility-level strategies to cope with financial shortfalls on user's access to care and the reasons users are charged for contraceptive services.

The considerable variation in free services by method in public facilities possibly reflects differing auxiliary costs associated with dispensing methods, with, for example, more staff time, training and medical equipment required to insert IUDs and implants compared to condoms, which are often available without a consultation. We found that injectable users were significantly less likely to report receiving this method for free compared to long-acting IUD and implants or even the pill, raising questions about the long-term cost burden to users, who require re-supply every three months for continued coverage, for this popular method.

Limitations

This study was limited in relying on the accuracy of women's self-report of their method, source and cost of FP. While current injectable users needed to recall how much was paid up to three months earlier, some current implant users were asked to report the amount paid up to three years prior to interview, though median length of implant use was less than 17 months. Additionally, we were only able to consider cost and source among women who were current users of FP. Findings are likely not generalisable to former implant or injectable users, particularly if they discontinued due to costs associated with obtaining their method of choice, or to prospective users who were discouraged from initiating FP due to costs associated.

The DHS question regarding contraceptive cost asked for the total paid for commodity and consultation, and it did not capture costs associated with time and travel to obtain the method. These may be significant, particularly for rural users. We were unable to estimate the share of OOP payment for FP from total income because the DHS does not collect information on household/individual income or expenditures. As such, we cannot draw conclusions about the extent to which the amount paid for FP represents an undue burden on individual users. Additionally, DHS household wealth quintiles may not

For peer review only - http://bmjopen.bmj.com/site/about/guidelines.xhtml

align with the poverty definition used to determine FP fee waivers or offer sufficient nuance to distinguish very disadvantaged households[34].

We acknowledge that the first consultation visit to initiate the contraceptive method may be longer, involving counselling and taking of medical history, than a re-supply visit and could result in increased cost. However, we compared results for initiators, users starting injectable and implant less than three months and three years, respectively, before the survey where the cost paid likely refers to the initiating consultation, against re-supply users, those starting the method more than three months or years prior to interview. Yet we found initiating users reported slightly lower mean costs than re-suppliers, though differences were not significant (results not shown).

Finally, FP budget implementation is done at the county-level in Kenya, yet the DHS FP cost question was intended to be representative at national, urban/rural and regional levels only[19] and thus county-level results could not be examined.

Conclusions

Removing or subsidising costs for the poor is a core component of an equitable system of user fees for healthcare, yet our findings highlight that the poorest contraceptive users in the public sector were as likely to pay for FP services as wealthier users. Kenya's National Reproductive Health Strategy (2009-2015) outlined pro-poor strategies and objectives to increase equity of FP access. The Kenyan government has made important progress in expanding FP access but more attention is needed to implementation of user fee policies, particularly to ensure the poorest receive affordable services and to account for geographic variation, ensuring recent efforts to reimburse facilities for lost user fee revenue are done at appropriate levels. However, public sector resources alone are unlikely to meet Kenya's growing demand for modern contraception. Policymakers should consider how government resources could be targeted at those least able to tap the private sector for FP care. While individual price discrimination offers one route to targeting public services to the poor, efforts could also focus resources—including outreach campaigns about patients' rights and correct fees—toward facilities in poor areas or toward increasing choice of affordable methods and accessible, high-quality providers for the poor. Fulfilling the promise of equity in FP access in Kenya demands turning policy intention into sustainable action from the national to facility-level.

List of abbreviations

DHS: Demographic & Health Surveys **FP**: Family planning **IUD:** Intrauterine device
KES: Kenyan Shillings **LMICs**: Low- and middle-income countries
NGO: Non-governmental organisation **OOP:** Out-of-pocket **USD**: United States Dollar

DECLARATIONS

Ethics approval

The DHS receive government permission and follow ethical practices including informed consent and assurance of confidentiality. The Research Ethics Committee of the London School of Hygiene and Tropical Medicine approved our secondary-data analysis.

Data sharing

The data that support the findings of this study are owned by the Demographic and Health Surveys (DHS) Program, operated by ICF International. Restrictions apply to the availability of these data, which

For peer review only - http://bmjopen.bmj.com/site/about/guidelines.xhtml

were used under license for the current study, and so are not publicly available. Data are available for free from the DHS Program website and available for researchers who apply for and meet the criteria for access. Legal access agreements do not allow the sharing of datasets to unregistered researchers.

Competing interests

The authors declare that they have no competing interests.

Funding statement

The research in this publication was supported by funding from MSD, through its MSD for Mothers program. MSD had no role in the design, collection, analysis and interpretation of data, in writing of the manuscript, or in the decision to submit the manuscript for publication. The content of this publication is solely the responsibility of the authors and does not represent the official views of MSD. MSD for Mothers is an initiative of Merck & Co., Inc., Kenilworth, N.J., U.S.A. Edwine Barasa is supported by a Wellcome Trust research training grant (#107527).

Authors' contributions

ER designed the research question, analysed data, and prepared the manuscript. LB, MD, CL and JB contributed to the design of the study. KW contributed to analysis of the data. LB, MD, FC and EB assessed interpretation of findings and contributed to manuscript revisions. JB, TA, KW and MLA reviewed and edited the manuscript. All authors read and approved the final manuscript.

REFERENCES

1 Munge K, Briggs AH. The progressivity of health-care financing in Kenya. *Health Policy Plan* 2014;**29**:912–20. doi:10.1093/heapol/czt073

2 Obare F, Warren C, Kanya L, *et al.* Community-level effect of the reproductive health vouchers program on out-of-pocket spending on family planning and safe motherhood services in Kenya. *BMC Health Serv Res* 2015;**15**:343. doi:10.1186/s12913-015-1000-3

3 Health Policy Initiative. The EQUITY Framework: Influencing Policy and Financing Reforms to Increase Family Planning Access for the Poor in Kenya. Washington, DC: : Futures Group 2010.

4 Haghparast-Bidgoli H, Pulkki-Brännström A-M, Lafort Y, *et al.* Inequity in costs of seeking sexual and reproductive health services in India and Kenya. *Int J Equity Health* 2015;**14**:84. doi:10.1186/s12939-015-0216-5

5 Holtz J. Achieving Universal Access to Family Planning Services. Bethesda, MD: : Sustaining Health Outcomes through the Private Sector (SHOPS) Plus Project, Abt Associates 2016.

6 Chuma J, Gilson L, Molyneux C. Treatment-seeking behaviour, cost burdens and coping strategies among rural and urban households in Coastal Kenya: an equity analysis. *Trop Med Int Heal* 2007;**12**:673–86. doi:10.1111/j.1365-3156.2007.01825.x

7 Korachais C, Macouillard E, Meessen B. How User Fees Influence Contraception in Low and Middle Income Countries: A Systematic Review. *Stud Fam Plann* 2016;**47**:341–56. doi:10.1111/sifp.12005

8 Sedgh G, Ashford LS, Hussain R. Unmet Need for Contraception in Developing Countries: Examining Women's Reasons for Not Using a Method. New York: : Guttmacher Institute 2016.

9 Sedgh G, Hussain R. Reasons for Contraceptive Nonuse among Women Having Unmet Need for Contraception in Developing Countries. *Stud Fam Plann* 2014;**45**:151–69. doi:10.1111/j.1728-4465.2014.00382.x

For peer review only - http://bmjopen.bmj.com/site/about/guidelines.xhtml

10 Janisch CP, Albrecht M, Wolfschuetz A, *et al.* Vouchers for health: A demand side output-based aid approach to reproductive health services in Kenya. *Glob Public Health* 2010;**5**:578–94. doi:10.1080/17441690903436573

11 Chuma J, Musimbi J, Okungu V, *et al.* Reducing user fees for primary health care in Kenya: Policy on paper or policy in practice? *Int J Equity Health* 2009;**8**:15. doi:10.1186/1475-9276-8-15

12 Chuma J, Maina T. Free Maternal Care and Removal of User Fees at Primary-Level Facilities in Kenya: Monitoring the Implementation and Impact—Baseline Report. Washington, DC: : Futures Group, Health Policy Initiative 2013.

13 National Coordinating Agency for Population and Development, Ministry of Medical Services, Ministry of Public Health and Sanitation, *et al.* Kenya Service Provision Assessment Survey 2010. Nairobi, Kenya: 2011.

14 National Reproductive Health Strategy, 2009-2015. Nairobi, Kenya: : Ministry of Public Health and Sanitation & Ministry of Medical Services (Republic of Kenya)

15 Kenya Reproductive, Maternal, Newborn, Child and Adolescent Health (RMNCAH) Investment Framework. Nairobi, Kenya: : Ministry of Health (Republic of Kenya) 2016.

16 Health Policy Initiative. FP/RH Access for the Poor in Kenya: EQUITY Framework Encourages Policy and Financing Reforms. Washington, DC: : Futures Group 2010.

17 POLICY Project. Targeting: A Key Element of National Contraceptive Security Planning. Washington, DC: : Futures Group 2003.

18 Health Policy Initiative. The cost of family planning in Kenya. Washington, DC: : Futures Group 2010.

19 Kenya National Bureau of Statistics, Ministry of Health, National AIDS Control Council, *et al.* Kenya Demographic and Health Survey 2014. Nairobi, Kenya and Rockville, MD: : ICF International 2015.

20 Hubacher D, Trussell J. A definition of modern contraceptive methods. *Contraception* 2015;**92**:420–1. doi:10.1016/j.contraception.2015.08.008

21 Rutstein SO, Johnson K. The DHS Wealth Index (DHS Comparative Reports #6). Calverton, MD: : ORC Macro 2004.

22 Filzmoser P, Gussenbauer J, Templ M. Detecting outliers in household consumption survey data. Vienna, Austria: : Vienna University of Technology 2016.

23 XE Currency Charts: USD to KES. http://www.xe.com/currencycharts/?from=USD&to=KES&view=5Y (accessed 5 Dec 2016).

24 Skordis-Worrall J, Pace N, Bapat U, *et al.* Maternal and neonatal health expenditure in Mumbai slums (India): a cross sectional study. *BMC Public Health* 2011;**11**:150. doi:10.1186/1471-2458-11-150

25 Stata. testnl — Test nonlinear hypotheses after estimation. https://www.stata.com/manuals13/rtestnl.pdf (accessed 11 Jan 2018).

26 Chakraborty NM, Wanderi J, Oduor C, *et al.* Assessing provision and equity in low and middle income country health markets: a study from Kenya. Oakland, CA: : Metrics for Management 2017.

For peer review only

For peer review only - http://bmjopen.bmj.com/site/about/guidelines.xhtml

27 Arur A, Gitonga N, O'Hanlon B, *et al.* Insights from innovations: Lessons from designing and implementing family planning / reproductive health voucher programs in Kenya and Uganda. Bethesda, MD: : Private Sector Partnerships-One project, Abt Associates Inc. 2009.

28 Njuki R, Obare F, Warren C, *et al.* Community experiences and perceptions of reproductive health vouchers in Kenya. *BMC Public Health* 2013;**13**:660. doi:10.1186/1471-2458-13-660

29 Keesara SR, Juma PA, Harper CC, *et al.* Why do women choose private over public facilities for family planning services? A qualitative study of post-partum women in an informal urban settlement in Kenya. *BMC Health Serv Res* 2015;**15**:335. doi:http://dx.doi.org/10.1186/s12913-015-0997-7

30 Agha S, Do M. The quality of family planning services and client satisfaction in the public and private sectors in Kenya. *Int J Qual Heal Care* 2009;**21**:87–96. doi:10.1093/intqhc/mzp002

31 Hutchinson PL, Do M, Agha S. Measuring client satisfaction and the quality of family planning services: a comparative analysis of public and private health facilities in Tanzania, Kenya and Ghana. *BMC Health Serv Res* 2011;**11**:203. doi:10.1186/1472-6963-11-203

32 Barker C, Mulaki A, Mwai D, *et al.* Assessing county health system readiness in Kenya: A review of selected health inputs. Washington, DC: : Futures Group 2014.

33 Nyikuri M, Tsofa B, Barasa E, *et al.* Crises and Resilience at the Frontline—Public Health Facility Managers under Devolution in a Sub-County on the Kenyan Coast. *PLoS One* 2015;**10**:e0144768. doi:10.1371/journal.pone.0144768

34 Wong KLM, Restrepo-Méndez MC, Barros AJD, *et al.* Socioeconomic inequalities in skilled birth attendance and child stunting in selected low and middle income countries: Wealth quintiles or deciles? *PLoS One* 2017;**12**:e0174823. doi:10.1371/journal.pone.0174823

For peer review only

For peer review only - http://bmjopen.bmj.com/site/about/guidelines.xhtml

FIGURE 1: METHOD MIX AND PROVIDER USE BY WEALTH QUINTILE AMONG CURRENT MODERN FP USERS

For peer review only

For peer review only - http://bmjopen.bmj.com/site/about/guidelines.xhtml

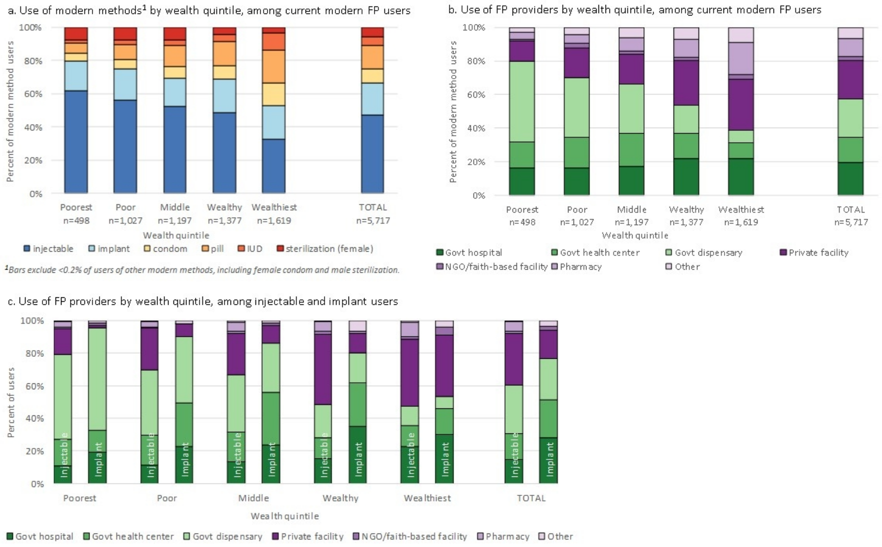

Method mix and provider use by wealth quintile among current modern FP users

338x190mm (96 x 96 DPI)

Supplementary Table 1

Summary of out-of-pocket payment (in USD) for injectable and implant users among users with non-zero expenditure, by most recent provider of the contraceptive method, with different methods for dealing with outliers.

a. Observations greater than two standard deviations from the mean recoded as equal to the mean.

	Govt hospital	Govt health centre	Govt dispensary	TOTAL PUBLIC	Private facility	NGO/faith-based facility	Pharmacy/ chemist	Other[1]	TOTAL
Injectable									
n	247	225	490	962	821	28	148	17	1,976
Mean cost in USD	$0.83	$0.78	$0.74	$0.75	$1.07	$1.01	$1.08	$1.06	$0.91
SD	0.412	0.399	0.385	0.343	0.277	0.560	0.278	0.280	0.349
25th percentile	$0.57	$0.57	$0.57	$0.57	$0.91	$0.57	$0.91	$1.14	$0.57
50th percentile (median)	$0.57	$0.57	$0.57	$0.57	$1.14	$0.80	$1.14	$1.14	$1.14
75th percentile	$1.14	$1.14	$1.14	$1.14	$1.14	$1.14	$1.14	$1.14	$1.14
Implant									
n	136	94	102	332	130	11	-	3	477
Mean cost in USD	$3.48	$2.90	$2.37	$2.97	$7.47	$6.43		$6.20	$4.31
SD	3.363	2.525	1.629	2.724	5.034	4.430		6.090	4.096
25th percentile	$2.28	$1.14	$1.14	$1.14	$3.42	$2.28		$1.14	$2.28
50th percentile (median)	$2.28	$2.28	$2.28	$2.28	$5.74	$9.12		$1.14	$2.28
75th percentile	$3.42	$3.42	$2.28	$3.42	$11.40	$9.12		$1.14	$5.70

b. Observations greater than two standard deviations from the mean recoded to missing.

	Govt hospital	Govt health centre	Govt dispensary	TOTAL PUBLIC	Private facility	NGO/faith-based facility	Pharmacy/ chemist	Other[1]	TOTAL
Injectable									
n	237	216	476	929	794	21	144	17	1,905
Mean cost in USD	$0.81	$0.74	$0.71	$0.74	$1.07	$0.82	$1.09	$1.06	$0.91
SD	0.387	0.327	0.330	0.346	0.282	0.338	0.282	0.280	0.355
25th percentile	$0.57	$0.57	$0.57	$0.57	$0.91	$0.57	$0.91	$1.14	$0.57
50th percentile (median)	$0.57	$0.57	$0.57	$0.57	$1.14	$0.80	$1.14	$1.14	$1.14
75th percentile	$1.14	$1.14	$1.14	$1.14	$1.14	$1.14	$1.14	$1.14	$1.14
Implant									
n	135	94	102	331	109	11	-	3	454
Mean cost in USD	$3.47	$2.90	$2.37	$2.97	$7.81	$6.43	-	$6.20	$4.24
SD	3.367	2.525	1.629	2.724	5.454	4.430	-	6.090	4.183
25th percentile	$2.28	$1.14	$1.14	$1.14	$2.85	$2.28	-	$1.14	$2.28
50th percentile (median)	$2.28	$2.28	$2.28	$2.28	$5.70	$9.12	-	$1.14	$2.28
75th percentile	$3.42	$3.42	$2.28	$3.42	$11.40	$9.12	-	$11.40	$5.70

c. Observations greater than two standard deviations from the mean recoded as equal to the value two standard deviations from the me[illegible]

	Govt hospital	Govt health centre	Govt dispensary	TOTAL PUBLIC	Private facility	NGO/faith-based facility	Pharmacy/ chemist	Other[1]	TOTAL
Injectable									
n	247	225	490	962	821	28	148	17	1,976
Mean cost in USD	$0.86	$0.80	$0.75	$0.79	$1.11	$1.13	$1.11	$1.06	$0.95
SD	0.454	0.419	0.396	0.419	0.331	0.628	0.319	0.280	0.412
25th percentile	$0.57	$0.57	$0.57	$0.57	$0.91	$0.57	$0.91	$1.14	$0.57
50th percentile (median)	$0.57	$0.57	$0.57	$0.57	$1.14	$0.80	$1.14	$1.14	$1.14
75th percentile	$1.14	$1.14	$1.14	$1.14	$1.14	$1.71	$1.14	$1.14	$1.14
Implant									
n	136	94	102	332	130	11	-	3	477
Mean cost in USD	$3.54	$2.90	$2.37	$3.00	$10.16	$6.43	-	$6.20	$5.06
SD	3.559	2.525	1.629	2.829	7.236	4.430	-	6.090	5.528
25th percentile	$2.28	$1.14	$1.14	$1.14	$3.42	$2.28	-	$1.14	$2.28
50th percentile (median)	$2.28	$2.28	$2.28	$2.28	$6.84	$9.12	-	$1.14	$2.28
75th percentile	$3.42	$3.42	$2.28	$3.42	$17.10	$9.12	-	$11.40	$5.70

For peer review only

d. Observations greater than 1.5 times the interquartile range recoded to missing.

	Govt hospital	Govt health centre	Govt dispensary	TOTAL PUBLIC	Private facility	NGO/faith-based facility	Pharmacy/ chemist	Other[1]	TOTAL
Injectable									
n	237	216	476	929	794	21	144	17	1,905
Mean cost in USD	$0.81	$0.74	$0.71	$0.74	$1.07	$0.82	$1.09	$1.06	$0.91
SD	0.387	0.327	0.330	0.346	0.282	0.338	0.282	0.280	0.355
25th percentile	$0.57	0.57	0.57	$0.57	0.912	0.57	0.912	1.14	$0.57
50th percentile (median)	$0.57	0.57	0.57	$0.57	1.14	0.798	1.14	1.14	$1.14
75th percentile	$1.14	1.14	1.14	$1.14	1.14	1.14	1.14	1.14	$1.14
Implant									
n	128	89	102	319	72	9	-	2	401
Mean cost in USD	$2.80	$2.43	$2.37	$2.56	$4.33	$5.12	-	$1.14	$2.93
SD	1.738	1.565	1.629	1.663	2.060	3.647	-	0.000	1.948
25th percentile	$2.28	$1.14	$1.14	$1.14	$2.28	$2.28	-	$1.14	$1.71
50th percentile (median)	$2.28	$2.28	$2.28	$2.28	$3.42	$2.28	-	$1.14	$2.28
75th percentile	$3.42	$2.85	$2.28	$3.42	$5.70	$9.12	-	$1.14	$3.42

e. Observations greater than 1.5 times the interquartile range recoded as equal to the value 1.5 times the interquartile range.

	Govt hospital	Govt health centre	Govt dispensary	TOTAL PUBLIC	Private facility	NGO/faith-based facility	Pharmacy/ chemist	Other[1]	TOTAL
Injectable									
n	247	225	490	962	821	28	148	17	1,976
Mean cost in USD	$0.86	$0.80	$0.75	$0.79	$1.10	$1.11	$1.11	$1.06	$0.95
SD	0.445	0.408	0.388	0.410	0.323	0.595	0.313	0.280	0.403
25th percentile	$0.57	$0.57	$0.57	$0.57	$0.91	$0.57	$0.91	$1.14	$0.57
50th percentile (median)	$0.57	$0.57	$0.57	$0.57	$1.14	$0.80	$1.14	$1.14	$1.14
75th percentile	$1.14	$1.14	$1.14	$1.14	$1.14	$1.71	$1.14	$1.14	$1.14
Implant									
n	136	94	102	332	130	11	-	3	477
Mean cost in USD	$3.25	$2.87	$2.37	$2.87	$7.27	$6.14	-	$5.92	$4.18
SD	2.515	2.424	1.629	2.274	3.583	3.994	-	5.751	3.394
25th percentile	$2.28	$1.14	$1.14	$1.14	$3.42	$2.28	-	$1.14	$2.28
50th percentile (median)	$2.28	$2.28	$2.28	$2.28	$6.84	$9.12	-	$1.14	$2.28
75th percentile	$3.42	$3.42	$2.28	$3.42	$10.83	$9.12	-	$10.83	$5.70

[1] Includes DHS response options: mobile clinic and other private medical

SD: Standard deviation

For peer review only

Supplementary Table 2

Out-of-pocket payment (in USD) for injectable and implant across all provider types among users with non-zero expenditure by socio–demographic characteristics.

	Injectable				*Implant*			
	n	Median	Mean (95% CI)		n	Median	Mean (95% CI)	
Wealth quintile								
Poorest	209	$0.80	$0.84 (0.77-0.91)		29	$2.28	$3.35 (1.88-4.81)	
Poor	417	$0.80	$0.82 (0.78-0.86)		89	$2.28	$2.78 (2.43-3.13)	
Middle	459	$0.80	$0.89 (0.84-0.93)	p<0.001	81	$2.28	$3.03 (2.54-3.53)	p<0.001
Wealthy	519	$1.14	$0.97 (0.93-1.01)		101	$2.28	$4.06 (2.83-5.30)	
Wealthiest	379	$1.14	$1.14 (1.08-1.21)		177	$5.70	$5.95 (4.74-7.17)	
Residence								
Urban	792	$1.14	$1.07 (1.03-1.11)	p<0.001	230	$3.42	$5.18 (4.15-6.21)	p=0.005
Rural	1191	$0.80	$0.85 (0.82-0.88)		246	$2.28	$3.49 (2.94-4.04)	
Educational attainment								
Less than primary	615	$0.91	$0.87 (0.84-0.91)		115	$2.28	$3.88 (2.90-4.85)	
Less than secondary	915	$1.14	$0.94 (0.90-0.98)	p<0.001	202	$2.28	$3.36 (2.78-3.94)	p=0.004
Secondary+	453	$1.14	$1.03 (0.98-1.08)		160	$3.42	$5.81 (4.49-7.14)	
Age group								
<20 years	77	$1.14	$0.92 (0.85-1.00)		9	$4.56	$3.50 (1.95-5.04)	
20–29 years	1032	$1.14	$0.94 (0.90-0.98)	p=0.897	226	$2.28	$4.20 (3.47-4.93)	p=0.594
30+ years	874	$1.14	$0.94 (0.90-0.97)		242	$2.28	$4.44 (3.50-5.38)	
Region†								
Central	207	$1.14	$1.07 (1.01-1.14)		87	$3.42	$4.51 (3.47-5.56)	
Coast	125	$1.14	$0.99 (0.87-1.11)		11	$2.28	$4.32 (1.35-7.29)	
Eastern	425	$0.91	$0.91 (0.85-0.96)		67	$3.42	$4.72 (3.79-5.64)	
Nairobi	183	$1.14	$1.18 (1.05-1.30)	p<0.001	51	$5.74	$8.03 (4.27-11.78)	p<0.001
Nyanza	315	$0.57	$0.84 (0.79-0.90)		66	$2.28	$2.90 (2.11-3.70)	
Rift Valley	495	$1.14	$0.93 (0.89-0.98)		129	$2.28	$4.08 (3.13-5.02)	
Western	232	$0.80	$0.81 (0.75-0.87)		64	$2.28	$2.58 (2.08-3.07)	

†Due to the very low modern contraceptive prevalence (<5%), results for the North Eastern region are not presented.

For peer review only

Supplementary Table 3

Among users with non-missing source of the method, percentage of modern contraceptive method by provider type.

	Govt hospital	Govt health center	Govt dispensary	Private facility	NGO/faith–based facility	Pharmacy/ chemist	Other[1]
**Method**							
Injectable	36.2%	49.4%	62.0%	65.6%	29.4%	24.6%	5.8%
Implant	27.4%	29.4%	20.9%	14.7%	17.8%	–†	10.7%
Condom	4.1%	3.1%	3.1%	1.6%	0.4%	13.2%	73.7%
Pill	7.3%	8.0%	9.2%	8.1%	4.6%	61.6%	7.8%
IUD	9.4%	5.6%	2.6%	7.3%	18.2%	–†	0.7%
Other modern methods[2]	15.7%	4.5%	2.2%	2.8%	29.7%	0.5%	1.3%
TOTAL	100%	100%	100%	100%	100%	100%	100%
Total number of users (weighted n)	*1,106*	*863*	*1,304*	*1,313*	*129*	*622*	*379*

[1] Includes DHS response options: mobile clinic and other private medical

[2] Includes other modern FP methods such as female and male sterilisation and female condoms.

† No respondents reported this provider and method.

For peer review only

Supplementary Table 4

Proportion reporting free family planning by provider among users initiating their long–acting contraceptive method before or after June 2013 abolition of fees.

	Govt hospital % (95% CI)	Govt health centre % (95% CI)	Govt dispensary % (95% CI)	TOTAL PUBLIC % (95% CI)
Initiated before June 2013				
IUD (n=898)	49.9 (36.6–63.2)	78.8 (59.5–90.4)	76.9 (56.4–89.6)	61.2 (51.5–70.1)
Implant (n=469)	51.3 (40.6–61.9)	62.7 (51.5–72.7)	57.8 (48.3–66.7)	56.6 (50.4–62.7)
Initiated from June 2013 onward				
IUD (n=48)	48.5 (26.4–71.2)	69.2 (41.6–87.6)	64.6 (32.1–87.6)	59.2 (42.8–73.7)
Implant (n=357)	61.2 (50.5–70.9)	63.2 (50.0–74.7)	65.6 (56.0–74.0)	63.4 (56.9–69.5)

For peer review only

STROBE Checklist for cross-sectional studies

	Item No.	Recommendation	Page No.
**Title and abstract**	1	(*a*) Indicate the study's design with a commonly used term in the title or the abstract	1
		(*b*) Provide in the abstract an informative and balanced summary of what was done and what was found	2
**Introduction**			
Background/rationale	2	Explain the scientific background and rationale for the investigation being reported	3
Objectives	3	State specific objectives, including any prespecified hypotheses	4
**Methods**			
Study design	4	Present key elements of study design early in the paper	4
Setting	5	Describe the setting, locations, and relevant dates, including periods of recruitment, exposure, follow-up, and data collection	4
Participants	6	(*a*) Give the eligibility criteria, and the sources and methods of selection of participants	4
Variables	7	Clearly define all outcomes, exposures, predictors, potential confounders, and effect modifiers. Give diagnostic criteria, if applicable	4-5
Data sources/ measurement	8*	For each variable of interest, give sources of data and details of methods of assessment (measurement). Describe comparability of assessment methods if there is more than one group	4-5
Bias	9	Describe any efforts to address potential sources of bias	4-5
Study size	10	Explain how the study size was arrived at	4
Quantitative variables	11	Explain how quantitative variables were handled in the analyses. If applicable, describe which groupings were chosen and why	4-5
Statistical methods	12	(*a*) Describe all statistical methods, including those used to control for confounding	5
		(*b*) Describe any methods used to examine subgroups and interactions	4-5
		(*c*) Explain how missing data were addressed	4
		(*d*) If applicable, describe analytical methods taking account of sampling strategy	5
		(*e*) Describe any sensitivity analyses	5
**Results**			
Participants	13	(a) Report numbers of individuals at each stage of study—eg numbers potentially eligible, examined for eligibility, confirmed eligible, included in the study, completing follow-up, and analysed	6,7,8
		(b) Give reasons for non-participation at each stage	n/a
		(c) Consider use of a flow diagram	n/a
Descriptive data	14	(a) Give characteristics of study participants (eg demographic, clinical, social) and information on exposures and potential confounders	Table 1 and Fig 1
		(b) Indicate number of participants with missing data for each variable of interest	4
Outcome data	15	Report numbers of outcome events or summary measures	Table 1,2,3&4
Main results	16	(*a*) Give unadjusted estimates and, if applicable, confounder-adjusted estimates and their precision (eg, 95% confidence interval). Make clear which confounders were adjusted for and why they were included	8
		(*b*) Report category boundaries when continuous variables were categorized	n/a
		(*c*) If relevant, consider translating estimates of relative risk into absolute risk for a meaningful time period	n/a

For peer review only

STROBE Checklist for cross-sectional studies

Other analyses	17	Report other analyses done—eg analyses of subgroups and interactions, and sensitivity analyses	9
**Discussion**			
Key results	18	Summarise key results with reference to study objectives	9
Limitations	19	Discuss limitations of the study, taking into account sources of potential bias or imprecision. Discuss both direction and magnitude of any potential bias	13-14
Interpretation	20	Give a cautious overall interpretation of results considering objectives, limitations, multiplicity of analyses, results from similar studies, and other relevant evidence	12-13,15
Generalisability	21	Discuss the generalisability (external validity) of the study results	13
**Other information**			
Funding	22	Give the source of funding and the role of the funders for the present study and, if applicable, for the original study on which the present article is based	15

For peer review only
